# In vitro and in vivo antiplasmodial evaluation of sugar-modified nucleoside analogues

**DOI:** 10.1038/s41598-023-39541-4

**Published:** 2023-07-28

**Authors:** Miklós Bege, Vigyasa Singh, Neha Sharma, Nóra Debreczeni, Ilona Bereczki, Poonam, Pál Herczegh, Brijesh Rathi, Shailja Singh, Anikó Borbás

**Affiliations:** 1 Department of Pharmaceutical Chemistry, University of Debrecen, Egyetem Tér 1, Debrecen, 4032 Hungary; 2 Institute of Healthcare Industry, University of Debrecen, Nagyerdei Körút 98, Debrecen, 4032 Hungary; 3 MTA-DE Molecular Recognition and Interaction Research Group, University of Debrecen, Egyetem Tér 1, Debrecen, 4032 Hungary; 4 Special Centre for Molecular Medicine, Jawaharlal Nehru University, New Delhi, 110067 India; 5 Department of Pharmacology and Toxicology, College of Pharmacy, University of Arizona, Tucson, AZ 85721 USA; 6 Laboratory for Translational Chemistry and Drug Discovery, Department of Chemistry, Hansraj College, University of Delhi, Delhi, India; 7 National Laboratory of Virology, University of Pécs, Ifjúság Útja 20, Pécs, 7624 Hungary; 8 Department of Chemistry, Miranda House, University of Delhi, Delhi, 110007 India; 9 Delhi School of Public Health, Institution of Eminence (IoE), University of Delhi, Delhi, 110007 India

**Keywords:** Drug discovery, Chemistry

## Abstract

Drug-resistant *Plasmodium falciparum* (*Pf*) infections are a major burden on the population and the healthcare system. The establishment of *Pf* resistance to most existing antimalarial therapies has complicated the problem, and the emergence of resistance to artemisinin derivatives is even more concerning. It is increasingly difficult to cure malaria patients due to the limited availability of effective antimalarial drugs, resulting in an urgent need for more efficacious and affordable treatments to eradicate this disease. Herein, new nucleoside analogues including morpholino-nucleoside hybrids and thio-substituted nucleoside derivatives were prepared and evaluated for in vitro and in vivo antiparasitic activity that led a few hits especially nucleoside-thiopyranoside conjugates, which are highly effective against *Pf*3D7 and *Pf*RKL-9 strains in submicromolar concentration. One adenosine derivative and four pyrimidine nucleoside analogues significantly reduced the parasite burden in mouse models infected with *Plasmodium berghei* ANKA. Importantly, no significant hemolysis and cytotoxicity towards human cell line (RAW) was observed for the hits, suggesting their safety profile. Preliminary research suggested that these thiosugar-nucleoside conjugates could be used to accelerate the antimalarial drug development pipeline and thus deserve further investigation.

## Introduction

Malaria continues to be a global health concern, with 247 million infections and 625,000 deaths worldwide in 2021, mainly among children and pregnant women^1^. The WHO African Region is responsible for a disproportionately large amount of the worldwide malaria burden. Human malaria is caused by five species of the mosquito-borne parasitic protozoa of the genus *Plasmodium*, the most prevalent and deadly of which is *Plasmodium falciparum (Pf)*, which is accountable for about 90% of malaria-related deaths^2^. Despite various malaria control and eradication attempts, most countries have not been able to eradicate the disease^3^. Drug resistance, toxicity, the lack of an effective vaccination, and low drug efficiency are all factors contributing to this^4^. The ongoing battle against *Plasmodium* drug resistance involves the exploration and development of wide range of therapeutics^5^. The current vaccine used for prevention of *Pf* malaria, RTS, S/AS01^6^ provides only moderate protection, although a pipeline of new vaccine candidates provides optimism. Further, the effective use of the frontline chemotherapy, Artemisinin Combination Therapy (ACTs), is now threatened by the emergence of resistance^7^.

Due to the lack of a vaccine and the growing resistance to current drugs, it is extremely important to develop new antimalarials and, in particular, to design drug candidates with a different structure and mechanism of action than those of current therapeutics^8^. Because pathogenic protozoa lack the ability to synthesize purines via de novo pathway, they rely on the salvage and reutilization of preformed purines for development and proliferation. They have various enzymes that are not found in mammals and can be used as therapeutic targets^9^. Nucleoside and nucleotide analogues are among the most promising class of potential antimalarial drugs because they can act as inhibitors in both the de novo pathway for pyrimidine nucleotide biosynthesis and the salvage pathway for purine nucleotides of *Pf*^10^. Acyclo nucleosides^11^, as well as 5’-thio nucleosides^12^ have been reported as potent inhibitors of *Pf.* Saturated six-membered nitrogen heterocycles, including morpholine, are common structural elements of antiplasmodial compounds^13,14^.

Recently, we have developed the synthetic methods for the preparation of new types of carbohydrate-modified nucleoside analogues, which can be divided into two major groups. The first group includes morpholino-nucleoside hybrids composed of morpholino unit(s) at the 5’-end and a nucleoside or 2’-deoxyribonucleoside at the 3’-end^15^. The second group consists of configurationally altered, l-*lyxo*, d-*xylo* or d-*arabino* configured nucleoside derivatives bearing a sulfanylmethyl-linked substituent at different positions of the furanose ring, which can be prepared by photoinitiated radical addition of various thiols onto C4’, C3’ or C2’ exomethylene moiety of nucleosides^16–19^. Regarding the great potential of nucleoside analogues^11,12^ as well as morpholine-containing derivatives^13,14^ in antimalarial therapy, we decided to test the antiplasmodial activity of the newly developed types of nucleoside analogues. Nucleoside transporters play a role in the uptake of certain antimalarial compounds, including chloroquine. These transporters facilitate the transport of nucleosides and nucleoside-like compounds across the parasite's plasma membrane. Chloroquine has been suggested to enter *Plasmodium falciparum*-infected erythrocytes via nucleoside transporters, which act as entry points for the compound into the parasite. This mechanism of uptake allows chloroquine to reach its target site and exert its antimalarial effects^20^.

**Figure 1:**
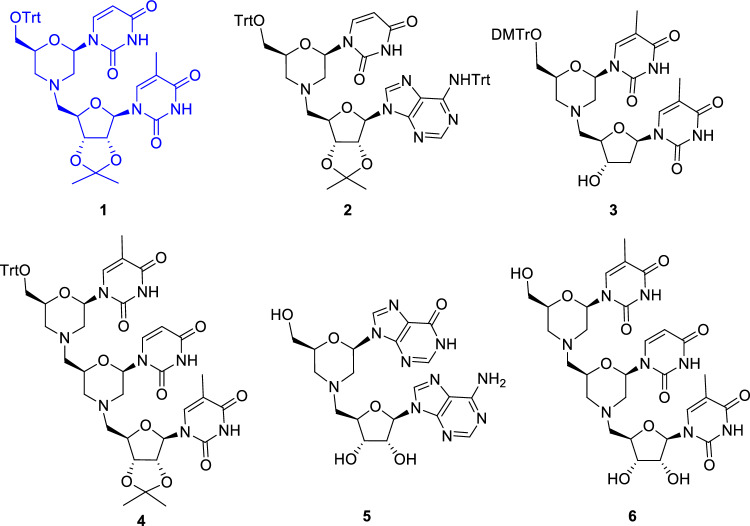
Morpholino-nucleoside hybrids^15^ (hit compound is highlighted in blue).

**Figure 2:**
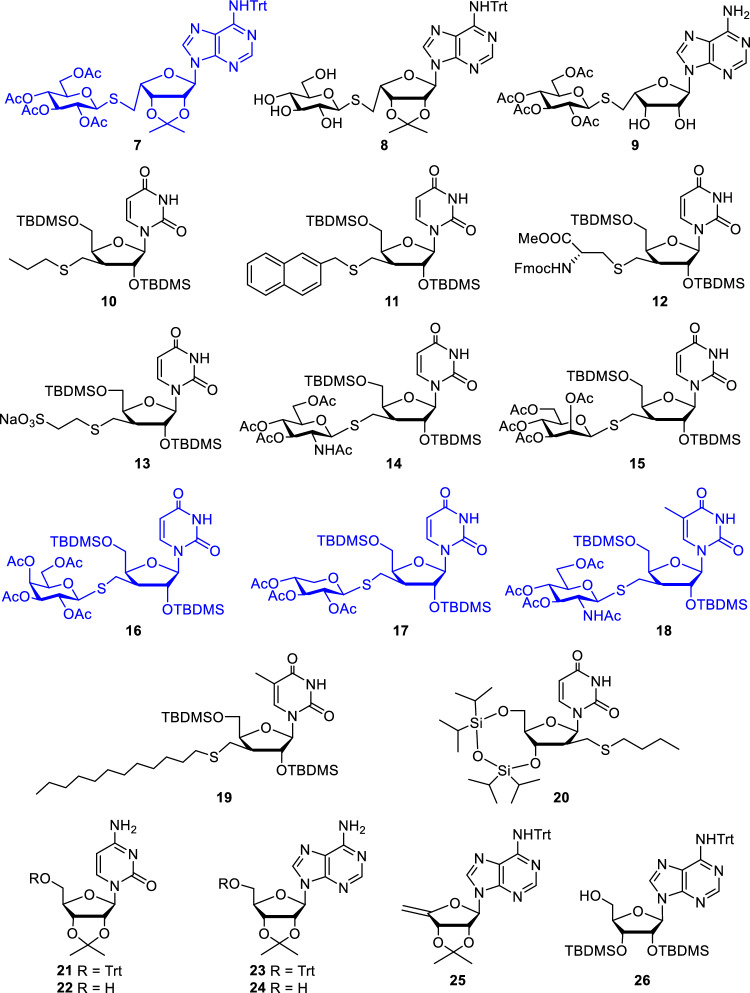
l-*Lyxo*-, d-*xylo* and d-*arabino* configured thiosubstituted nucleoside analogues **7**–**20** and nucleoside reference compounds **21**–**26** (hit compounds are highlighted in blue).

Previously prepared morpholino nucleoside hybrids (Fig. 1)^15^ were included in the biological studies, and a new library of thio-substituted nucleosides (Fig. 2) was also generated to investigate the structural diversity of thiol substituents and to find the group's bioactive components. In this manuscript, we present these two sets of nucleoside-based hybrid molecules that possess antiplasmodial activity based on their evaluation against chloroquine sensitive (*Pf*3D7) and resistant strains (*Pf*RKL-9). Top five analogues showed inhibitory concentrations (IC_50_) in the submicromolar range, which were further tested against the chloroquine resistant strain *Pf*RKL-9 and then in vivo effectiveness in mice model.

## Results and discussion

### Chemistry

**Figure 3:**
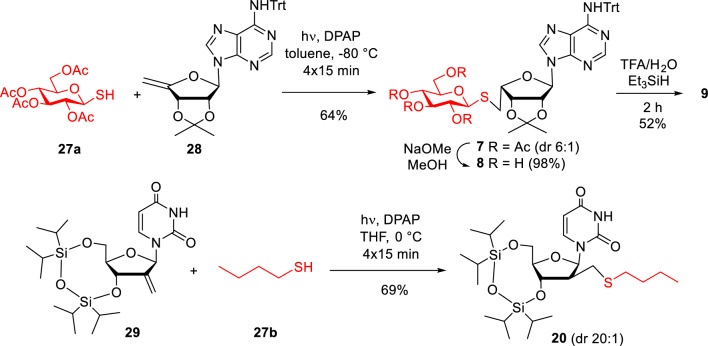
Thio-click-based synthesis of 5’- and 2’-modified nucleoside analogues.

The morpholino-nucleoside hybrids **1–6** involved in antimalarial evaluation are oligonucleotide analogues composed of one or two morpholino units and a natural ribo- (**1**, **2**, **4**–**6**) or 2’-deoxyribonucleoside (**3**) linked through the nitrogen atom of the morpholine ring (Fig. 1). These hybrid derivatives were prepared from nucleoside 2’,3’-dialdehydes in a double reductive amination-cyclization reaction with 5'-aminonucleosides^15^. The compounds were subjected to the antimalarial tests in both protected (**1**–**4**) and free forms (**5** and **6**). The second set of compounds tested includes nucleoside analogues derived from adenosine (**7**–**9**), uridine (**10**–**17** and **20**), and 5-methyluridine (**18** and **19**) bearing a thioether-linked substituent at the 5-', 3’- or 2'-position of the furanose unit (Fig. 2). A specific feature of the thio-substituted nucleoside analogues **7**–**20** is that they contain a configurationally modified furanose unit instead of natural d-ribose. Using our recently established photoinduced thiol-ene coupling method^21^, the l-*lyxo* (**7**–**9**), d-*xylo* (**10**–1**9**), and d-*arabino* (**20**) configured nucleoside analogues could be obtained with high stereoselectivity by radical mediated addition reactions between C4’-, C3’- or C2’-exomethylene nucleosides and various thiols including amino acid derivatives, 1-thiosugars or alkyl mercaptans^16–19^. In addition to the furanose-modified nucleoside analogues **7**–**20**, some simple, protected or partially protected nucleoside derivatives **21**–**26** were included in the antimalarial studies as reference compounds. The preparation of **10,**^16^ **19**^18^ and **21**–**26**^15,17^ has previously been reported, the synthesis of **7**–**9**, **11**–**18** and **20** is shown in Fig. 3 and Table 1.

**Table 1:** Thio-click-based synthesis of 3’-modified nucleoside analogues **11**–**18**.^a^


Alkene	Thiol	Solvent	Product	d-*xylo*: d-*ribo*^b^	Yield (%)^c^
**30**		THF	**11**^d^	10:1	61
**30**		THF	**12**	15:1	64
**30**		DMF	**13**^d^	10:1	80
**30**		Toluene-MeOH	**14**	12:1	85
**30**		Toluene- CH_2_Cl_2_	**15**	50:1	69
**30**		Toluene-CH_2_Cl_2_	**16**	18:1	86
**30**		Toluene-CH_2_Cl_2_	**17**	12:1	93
**31**		Toluene-MeOH	**18**	25:1	77

^a^The reactions were performed in a borosilicate vessel by irradiation with a Hg-lamp (λ_max_ = 365 nm) without any caution to exclude air or moisture.

^b^Diastereomeric ratio of products determined by ^1^H NMR.

^c^Overall yield of products isolated by column chromatography.

^d^The reaction was performed at − 40 °C.

First, the adenosine-derived nucleoside analogue **7** was prepared by reacting 1-thioglucose derivative **27a** with the 4’-exomethylene adenosine derivative **28**^17^ under previously optimized thiol-ene coupling conditions using the photoinitiator 2,2-dimethoxy-2-phenylacetophenone (DPAP) and irradiation with UVA light (λ_max_ = 365 nm) (Fig. 3). The photoinitiated thiol-ene reaction begins with the generation of a thiyl radical from the thiol by light-irradiation in the presence of the initiator, then occurs by a free-radical chain mechanism, including a propagation step and a chain transfer step, to produce an anti-Markovnikov type thioether product with full regioselectivity^21,22^. We used several short irradiation cycles, always adding a new dose of initiator to the reaction mixture, because this has been shown to be more effective than longer-term continuous irradiation^16,22^. Accordingly, alkene **28** and thiol **27a** were reacted by UV irradiation for 4 × 15 min in the presence of 4 × 0.1 equiv. of DPAP. Addition reaction at − 80 °C gave the thioglucose-nucleoside conjugate **7** as a 3:1 mixture of the l-*lyxo* and d-*ribo* diastereoisomers. After chromatographic purification, a 6:1 mixture highly enriched in the l-lyxofuranosyl major product was obtained (isomeric purity of **7** was 86%).

We performed removal of protecting groups of **7** to explore their role in antimalarial ativity. Deacetylation under Zemplén conditions yielded **8**, while simultanenous deprotection of the isopropylidene and triphenylmethyl groups with 90% aqueous TFA in the presence of Et_3_SiH provided compound **9**.

To produce a 2’-modified nucleoside analogue for the antimalarial assay, the 2’-*C*-exomethylene-3',5'-*O*-silylene-acetal derivative of uridine (**29**) was prepared and subjected to a photoinduced hydrothiolation reaction with butyl mercaptane **27b**^19^. The reaction proceeded with good yield and almost complete stereoselectivity providing the expected d-*arabino* configured 2’-*C*-butylsulfanylmethyl nucleoside **20** with 69% yield and an isomeric purity of 91% (20:1 d-*arabino*: d-*ribo* ratio of **20**).

Next, we prepared a small library of C3’-substituted d-*xylo* configured nucleoside analogues by addition of thiols **27c**-**27f** onto 3’-*C*-exomethylene-2’,5’-di-*O*-*tert*-butyldimethylsilyl-uridine **30** and -ribothymidine **31** (Table 1). The applied thiols included 2-mercaptomethyl naphthalene **27c**, l-cysteine derivative **27d**, Mesna **27e**, and *O*-acetylated 1-thiohexopyranoses **27f**-**27i**. The reactions were performed in various solvents selected based on the solubility of the reactants and cooling was used to achieve high diastereoselectivity. Based on our previous results, the thiol-ene couplings were carried out at low temperature (− 40/− 80 °C) in order to achieve high conversion and high stereoselectivity^16–19,21^. Using such conditions, the hydrothiolated products (**11**–**18**) with the expected d-*xylo* configuration were indeed obtained efficiently and with excellent diastereomeric excesses.

### Biological evaluation

#### Antimalarial activity by SYBR green I based fluorescence assay

To begin with, cell-based assay was performed to evaluate the inhibitory activities of all 26 compounds (**1**–**26**) at 1 µM and 10 µM concentrations on the asynchronous culture of *Pf*3D7 chloroquine-sensitive strain. Growth of one intraerythrocytic cycle of *Pf* was monitored after treated with compounds whereas untreated parasites were considered as control. At 10 µM concentration, the majority of the molecules inhibited more than 50% of the parasites (Fig. S1). Five of the twenty-six compounds, morpholino-nucleoside hybrid **1** and thiosubstituted nucleoside analogues **7**, **16**, **17** and **18** were observed to have significant growth inhibition in a dose dependent manner against *Pf*3D7. Morpholino-nucleoside hybrids **3** and **5** also showed remarkable actvity against *Pf*3D7 but they have cytotoxic properties (vide infra). Other compounds, except for the above mentioned seven derivatives showed insignificant percent growth inhibition as compared to untreated control against 3D7 strain of *Pf*.

**Figure 4:**
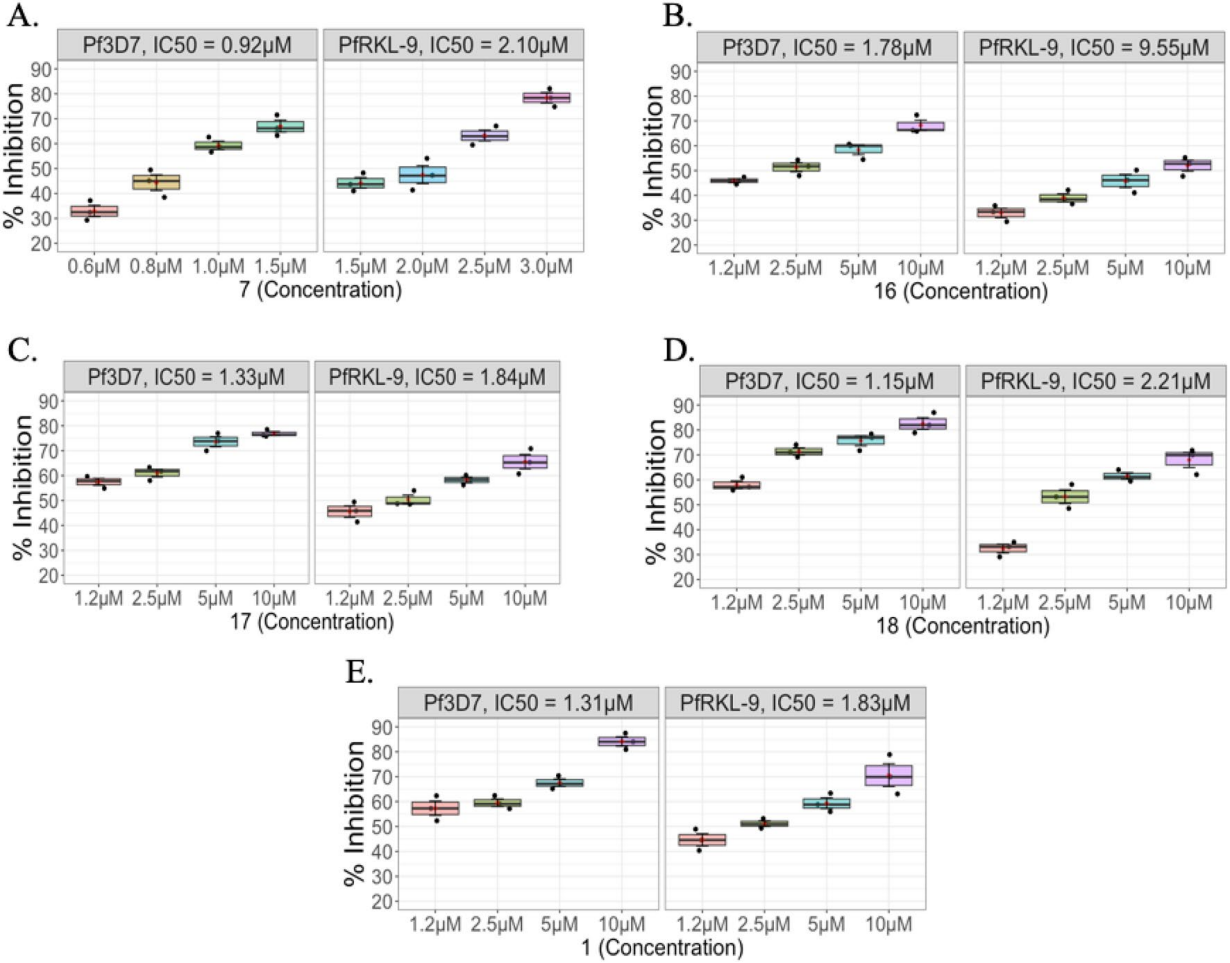
Estimation of in vitro growth inhibition and half maximal inhibition concentration (IC_50_) of hit compounds **7**, **16**, **17**, **18** and **1** against *Pf* 3D7 and *Pf* RKL-9 strains (**A–E**). Graph Pad Prism 9 software was used to calculate IC_50_ values. Graph showing percent inhibition for the same compound. Experiment was done in triplicate and data expressed as mean values ± SD.

Half maximal inhibition concentration (IC_50_) of the five hit molecules (**1**, **7**, **16**–**18**) was measured against *Pf*3D7 and chloroquine resistant strain *Pf*RKL-9 (Fig. 4). In this assay, synchronized late trophozoite parasites were treated with different concentrations of the compounds^23^. The percent growth inhibition of the parasites was evaluated by SYBR Green assay. The IC_50_ values of compounds **7**, **16**, **17**, **18** and** 1** against *Pf*3D7 were found to be 0.92 ± 1.8 µM, 1.78 ± 1.3 µM, 1.33 ± 1.07 µM, 1.15 ± 1.5 µM and 1.31 ± 1.1 µM, respectively. Next, all five compounds **7**, **16**, **17**, **18** and** 1** were evaluated against resistant strain *Pf*RKL-9 and displayed IC_50_ as 2.1 ± 1.3 µM, 9.55 ± 1.7 µM, 1.84 ± 1.06 µM, 2.21 ± 1.3 µM and 1.83 ± 0.7 µM, respectively. The results are depicted in Fig. 4 and Table 2. The IC_50_ values of chloroquine against *Pf*3D7 and *Pf*RKL-9 were found to be 25 nM and 125 nM, respectively. Although, compounds **3** and **5** showed remarkable growth inhibition with IC_50_ values of 1.19 ± 1.1 µM and 1.34 ± 2.1 µM against *Pf*3D7; and 4.5 ± 2.5 µM and 6.23 ± 1.5 µM against *Pf*RKL-9 (Fig. S2) but they showed cytotoxicity towards human RBCs (Fig. S3). Due to their cytotoxic nature, these compounds were not considered for further experiments.

**Table 2:** Calculated logP values and in vitro biological effects of the hit compounds.

Compound	clogP	IC_50_ (µM) against *Pf3D7*	IC_50_ (µM) against *PfRKL-9*	Cell^a^ survival (%) at 500 µM
	3.7706	1.31 ± 1.1	1.83 ± 0.7	45%
	5.6566	0.92 ± 1.8	2.1 ± 1.3	55%
	5.1384	1.78 ± 1.3	9.55 ± 1.7	52%
	4.9244	1.33 ± 1.07	1.84 ± 1.06	51%
	5.0137	1.15 ± 1.5	2.21 ± 1.3	60%
	5.0603	0.025 (25 nM)	0.125 (125 nM)	–

^a^embryonic kidney cell lines, HEK-293.

### Structure–activity analysis

The in vitro evaluation of compounds **1**–**26** against the chloroquine sensitive *Pf*3D7 strain (Fig. S1) allows preliminary analysis of the structure-antimalarial activity relationships of the two new nucleoside chemotypes, morpholino-nucleoside hybrids (**1**–**6**) and thiosubstituted nucleoside analogues (**7**–**20**).

Five of the six morpholino-nucleoside hybrids tested showed a remarkable ~ 50% inhibitory effect against *Pf*3D7 strain at a concentration of 1μM (compounds **1**–**5**, Fig. S1A), which clearly proves the high antimalarial potential of these structures.

Among the configurationally altered, thiosubstituted analogues, the thiosugar-containing derivatives were found to have excellent activity against *Pf* (Fig. S1). The configuration of the thiosugar affected the antimalarial activity to some extent, compounds having *gluco*- and *galacto*-configured substituents (**7**, **16**–**18**) showed excellent, while the mannopyranoside conjugate **15** showed a more moderate growth inhibitory effect. However, it is important to note that even the mannopyranoside-containing nucleoside **15** showed a stronger inhibitory effect (~ 50% inhibition at 1 μM concentration) than the alkylthio-substituted compounds **10**, **11** and **19** (~ 5–25% inhibition at 1 μM), suggesting that the thiopyranoside-nucleoside conjugates can have significant antimalarial potential. We have found that the place of substitution did not affect the antimalarial activity, the introduction of a suitable thiosugar in either the 5'- (**7**) or 3'-position (**16**–**18**) resulted in highly active derivatives.

Our study with compounds **7**–**9** suggests that appropriate lipophilicity is crucial for the activity of thiosubstituted nucleoside analogues. While the deacetylation (**7** → **8**) did not significantly affect the antimalarial activity, the removal of the lipophilic trityl groups (**7** → **9**) resulted in a significant decrease in growth inhibitiory effect (Fig. S1A). For the 1-thiosugar-containing hit compounds, the lipophilicity value is clogP ~ 5 (Table 2).

The antimalarial effect of purine nucleoside analogues based on the inhibition of purine metabolism pathway is well documented^10–12^, but there are hardly any results in the literature for pyrimidine-type antimalarial agents^24^. In this context, it is noteworthy that among the new derivatives tested in this study, several pyrimidine nucleoside analogues (**1**, **3** and **4** from the morpholino-nucleoside hybrids and **12**, **15–18** and **20** from the thiosugar-nucleoside chemotype) showed good/excellent inhibitory effect against *Pf*.

### Effect of compounds on human cell lines

Effect of compounds on human RBCs was checked with the help of spectrophotometry by measuring the lysis of human red blood cells (hRBCs). Compounds were tested at 0.5, 1, 5, 10 and 20 μM concentrations with 10% (v/v) RBCs suspension for 1 h and observed the percentage of RBCs lysis by compounds **7**, **16**, **17**, **18** and **1**.

**Figure 5:**
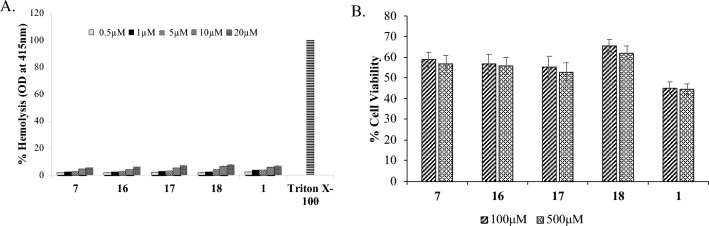
(**A**) Effect of compounds (**7**, **16**, **17**, **18** and **1**) on human RBCs. Effect of compounds on human RBCs was checked at 0.5, 1, 5, 10, 20 µM concentration. Absorbance was taken at 415 nm indicated no significant lysis when treated with compounds upto 20 µM. (**B**) Estimation of in vitro cytotoxicity effect of selected analogues against embryonic kidney cell lines (HEK-293) at 100 µM and 500 µM.

No significant lysis of erythrocytes was observed at concentration of 20 µM (Fig. 5A), while compounds **3** and **5** showed toxicity (Fig. S3). To evaluate the toxic effects of the potent compounds on human RAW cells, MTT assay was carried out and no apparent toxicity effect was observed for compounds **7**, **16**, **17**, **18** and** 1** up to 500 µM concentration (Fig. 5B). Table 2 summarizes the in vitro bioassay results and clogP values of the hit compounds and the reference compound chloroquine.

### Mitochondrial membrane disruption potential (Δψ_m_)

**Figure 6:**
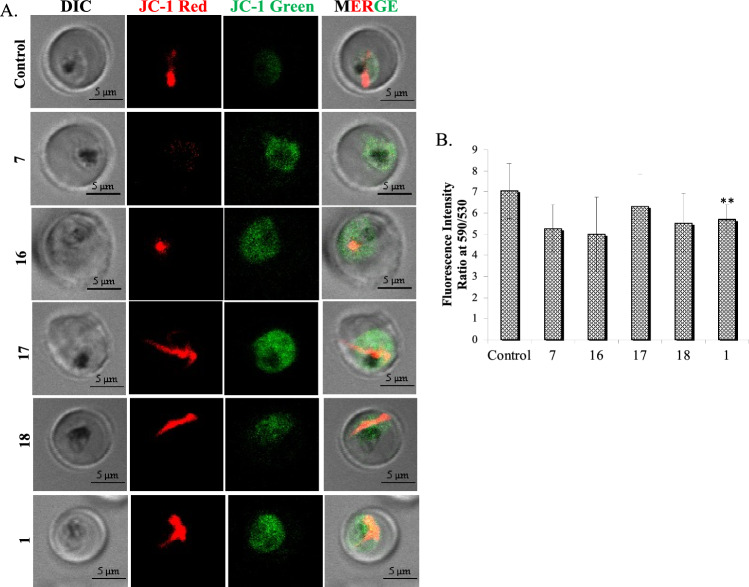
(**A**) Fluorescence images of parasites displaying monomeric JC-1 (green) in the cytoplasm and JC-1 aggregates (red) in mitochondria. The first row (control) exhibits untreated parasites with bright red signal at 590 nm indicating a functional mitochondrion. Subsequent rows exhibit the effects of the **7**, **16, 17, 18** and **1** with bright green and faint red signals. (**B**) The fluorometric ratio of JC-1 (aggregates)/JC-1 (monomeric) in the parasite population after treatment with **7**, **16, 17, 18** and** 1** *vs*. untreated control. Data expressed are mean values ± SD. **P < 0.01 *vs*. control: Dunnett’s test. Experiments were carried out in triplicate.

Mitochondrial membrane potential is a marker of mitochondria’s functional status. Modification in mitochondrial membrane potential leading to mitochondrial dysfunction triggers cell death^25,26^. Mitochondrial dysfunction was detected by using membrane permeant JC-1 dye. JC-1 is a cationic probe, due to electronegative environment inside the functionally active mitochondria with high Δψm, it aggregates in energised mitochondria and gives red color fluorescence at 590 nm, but in case of low Δψm (depolarized state), JC-1 remain in monomeric form and gives green color fluorescence at 530–10 nm. Decreased ratio of red/green fluorescence intensity of compound treated sample as compared to untreated control indicates depolarized mitochondrial membrane^27,28^. The damage to the parasite's mitochondria after treatment with compounds **7**, **16**, **17**, **18** and** 1** was measured according to the method reported previously^28^. Because of lipophilic nature of the JC-1, it is cell permeable and emits a green signal (525 nm) in the cytoplasm, but the high transmembrane potential of functional mitochondria causes it to aggregate and emit a red signal (590 nm). In contrast, as shown in Fig. 6, the parasite treated with compounds showed a considerable decrease in JC-1 red staining and an increase in diffused green mitochondrial fluorescence. The loss of mitochondrial membrane potential was demonstrated by a significant fall in the red/green ratio of the JC-1 stained counts in parasites treated with compounds. The parasites treated with IC_50_ concentrations of compounds **7**, **16, 17, 18** and** 1** exhibited reduced red/green ratio of 5.26 ± 1.13, 4.98 ± 1.77, 6.28 ± 1.55, 5.53 ± 1.40 and 5.69 ± 0.70. The untreated control parasite showed the red/green ratio of 7.04 ± 1.30 (Fig. 6A and B).

### In vivo antimalarial activity of compound 7 against *P. berghei ANKA*

**Figure 7:**
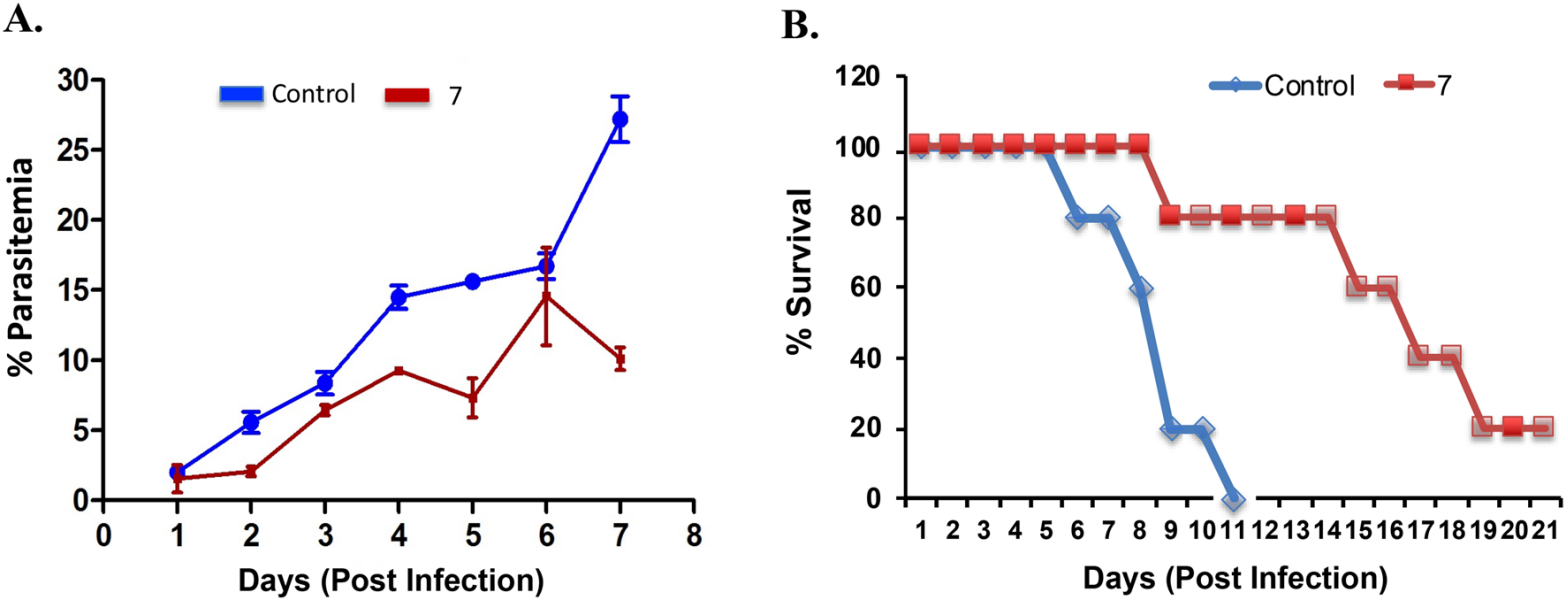
Efficacy of compound **7** on *P. berghei* ANKA infected Balb/c mice. Untreated mice were taken as control. Infected mice was administered intra-peritoneally with **7** (50 mg/kg body weight; n = 5) for seven consecutive days. (**A**) Percentage parasitemia was calculated by Giemsa-stained thin blood smears from day 1 to day 7 post infection; (**B**). Survival of mice was observed for 21 days post-infection using Kaplan–Meier survival analysis and statistical differences in animal survival was analyzed by a log rank test.

The most potent compound, the 1-thioglucose-adenosine conjugate **7** with purity level of > 95% (Fig. S4) was evaluated for antiplasmodial activity in mice model. In vivo antiplasmodial efficacy of **7** was assessed in *P. berghei* ANKA infected BALB/c mice model. Compound dose was given intra-peritoneal injections for seven days in a row. To check the parasitemia, thin blood smears were made from *P. berghei* ANKA infected mice for up to seven consecutive days. At a dose of 50 mg/kg, compound **7** showed 60% inhibition against the rodent-infecting *P. berghei ANKA.* The percentage parasitemia of group of mice treated with **7** was observed to be 10% as compared with the control wherein percentage parasitemia was 25% on the seventh day (Fig. 7A). Compound **7** was able to effectively reduce the parasite load compared to a control group of untreated mice for seven consecutive days post infection.

Groups of mice were observed for mean survival rates for 21 days post-infection. Mean survival time (MST) was calculated, which showed an improvement in mean survival time of mice treated with 50 mg/kg of **7** (MST = 14 days) compared to the control (MST = 10 days). It indicates that compound **7** showed potent antiplasmodial activity against *P. berghei* ANKA in vivo at a dose of 50 mg/kg and significantly prolonged survival of mice compared to the control group (Fig. 7B). To make relevant inferences, statistical analyses were carried out on the in vivo data using Graphpad Prism data sheets.

## Conclusion

Drug-resistant malaria has emerged as a severe threat to worldwide malaria management, necessitating the development of novel antimalarials that are effective against both drug-susceptible and drug-resistant malaria. Hybrid molecules that utilizes new entities with different pharmacophores represent a rational approach in development of novel therapeutics. The present work demonstrates that nucleoside-1-thiosugar hybrids, as synthetic products without cytotoxicity, might be suitable candidates for the development of antimalarial agents against *Pf*. The IC_50_ concentrations of the aforementioned nucleoside analogues were determined on *Pf 3D7* and *PfRKL-9* strains and were found to be between 0.95 µM and 2.21 µM. These analogues, when applied at IC_50_ concentrations, resulted in depolarization of the membrane potential of treated malaria parasites, leading to cell death. A 50 mg/kg dose of hit compound **7** administered to mice infected with the rodent parasite *P. berghei* ANKA showed up to a 60% reduction in parasite load, along with prolonged survival of the mice.

In addition to 1-thiosugar-nucleoside conjugates, our study also identified morpholino-nucleoside hybrids as a new chemotype of antimalarial drug candidates. Unfortunately, in the investigated morpholino-nucleoside hybrids, the strong antimalarial effect was usually accompanied by significant cytotoxicity, therefore further optimization is required for this chemotype in order to develop effective and safe antimalarial lead compounds.

## Materials and methods

### General

Nucleoside derivatives **1–6**^17^, **10**^16^ and **19**^18^, naphtylmethylmercaptane **27c**^29^, Fmoc-cysteine derivative **27d**^17^, 1-thiosugars **27f-i**^30^, adenosine-4’-exomethylene **28**^16^, uridine-2’-exomethylene **29**^31^, uridine-3’-exomethylene **30**^16^ and 5-methyluridine-3’-exomethylene **31**^16^ were prepared according to literature procedures. 2,2-Dimethoxy-2-phenylacetophenone (DPAP), *n*-butyl mercaptane **27b**, and MesNa **27e** were purchased from Sigma-Aldrich Chemical Co. and used without further purification. Optical rotations were measured with a Perkin-Elmer 241 automatic polarimeter at 20 °C. Reactions were monitored by TLC (Kieselgel 60 F_254_, Merck) with detection by UV-light (254 nm) and immersing into sulfuric acidic ammonium molybdate solution or 5% ethanolic sulfuric acid followed by heating. Purifications were performed on silica gel 60 (Merck, 0.040–0.063 mm). The ^1^H NMR (360 and 400 MHz) and ^13^C NMR (90 and 100 MHz) spectra were recorded with Bruker DRX-360 and Bruker DRX-400 spectrometers at 25 °C. Chemical shifts are referenced to Me_4_Si (0.00 ppm for ^1^H) and to the residual solvent signals (CDCl_3_: 77.2, DMSO-d_6_: 39.5, CD_3_OD: 49.0 for ^13^C). Two-dimensional COSY and ^1^H − ^13^C HSQC experiments were used to assist NMR assignments^16^. The diastereomeric ratio of the new compounds were determined on the basis of their ^1^H NMR spectra as described earlier^18,19^. MALDI-TOF MS measurements were carried out with a Bruker Autoflex Speed mass spectrometer equipped with a time-of-flight (TOF) mass analyzer. In all cases 19 kV (ion source voltage 1) and 16.65 kV (ion source voltage 2) were used. For reflectron mode, 21 kV and 9.55 kV were applied as reflector voltage 1 and reflector voltage 2, respectively. A solid phase laser (355 nm, ≥ 100 μJ/pulse) operating at 500 Hz was applied to produce laser desorption and 3000 shots were summed. 2,5-Dihydroxybenzoic acid (DHB) was used as matrix and F_3_CCOONa as cationising agent in DMF. ESI-QTOF MS measurements were carried out on a maXis II UHR ESI-QTOF MS instrument (Bruker), in positive ionization mode^32^. The following parameters were applied for the electrospray ion source: capillary voltage: 3.5 kV; end plate offset: 500 V; nebulizer pressure: 0.8 bar; dry gas temperature: 200 °C and dry gas flow rate: 4.5 L/min. Constant background correction was applied for each spectrum; the background was recorded before each sample by injecting the blank sample matrix (solvent). Na-formate calibrant was injected after each sample, which enabled internal calibration during data evaluation. Mass spectra were recorded by otofControl version 4.1 (build: 3.5, Bruker) and processed by Compass DataAnalysis version 4.4 (build: 200.55.2969).

The photoinitiated reactions were carried out in a borosilicate vessel by irradiation with a low-pressure Hg-lamp (Osram Supratec UV, HTC 150–211, 150 W, 230 V, R7s) giving maximum emission at 365 nm, without any caution to exclude air or moisture^16^. The experimental set-up consists of the reaction vessel and the cooling medium (acetone–liquid nitrogen mixture) in a Dewar flask and an UV-lamp. Before irradiation, the entire set-up is covered by an aluminum foil tent to protect the laboratory personnel against UV light^16,30^. The purity of hit compounds was assessed on HPLC system (Gilson, USA) with an analytical column (C18) and a Thermo Separation Spectra SERIES UV100 detector coupled with software. The mobile phase for the analysis was prepared from acetonitrile and water (v/v) and compounds showed purity > 95%.

The study was carried out accordingly to relevant guidelines. Mice-based in vivo experiments are reported in accordance with ARRIVE guidelines (https://arriveguidelines.org).

### Chemical synthesis

#### General method for photoinitiated free radical thiol-ene reaction^16,30^

The corresponding alkene, thiol and DPAP (0.1 eqiuv/alkene) were dissolved in the given solvent to obtain a solution with a concentration range of 0.2–0.5 M for the alkene (the solution should be as concentrated as possible). The reaction mixture was cooled to the given temperature and irradiated with UV-light for 15 min. After irradiation another 0.1 eqiuv. of DPAP was added and the irradiation continued for another 15 min. The addition of 0.1 equiv. of DPAP and the irradiation was repeated two more times. The solvent was evaporated in *vacuo* and the crude product was purified by flash column chromatography.

### 1-[2’,3’-*O*-isopropylidene-5’-*S*-(2,3,4,6-tetra-*O*-acetyl-β-d-glucopyranosyl)-5’-thio-α-l-lyxofuranosyl]-*N*-trityl-adenine (7)

Compound **28** (200 mg, 0.376 mmol), 1-thioglucose-peracetate **27a** (160 mg, 0.452 mmol, 1.2 equiv.) and DPAP (9.6 mg, 0.0376 mmol, 0.1 equiv.) dissolved in toluene (2 mL) and were reacted at − 80 °C according to the general method. The residue was purified by flash column chromatography (gradient elution hexane/acetone 85/15 → 8/2 → 6/4) to give compound an inseparable 3:1 mixture of 1-[2’,3’-*O*-isopropylidene-5’-*S*-(2,3,4,6-tetra-*O*-acetyl-β-d-glucopyranosyl)-5’-thio-α-l-lyxofuranosyl]-*N*-trityl-adenine (**7**) and its 4’-epimer 2’,3’-*O*-isopropylidene-5’-*S*-(2,3,4,6-tetra-*O*-acetyl-β-d-glucopyranosyl)-5’-thioadenosine (213 mg, 64%, ~ 3:1 mixture of l-*lyxo*: d-*ribo* isomers) as an amorphous solid. After a second chromatographic purification, the diastereomeric purity of **7** was increased to l-*lyxo*: d-*ribo* 6:1. Rf = 0.08 (hexane:acetone 8:2), ^1^H NMR (400 MHz, CDCl_3_) *δ* (ppm) 7.98, 7.83 (2xs, 2 × 1H, H-2, H-8), 7.34 (d, *J* = 7.1 Hz, 8H, aromatic), 7.29–7.19 (m, 13H, aromatic), 6.98 (s, 1H, N*H*), 5.98 (s, 1H, H-1’), 5.48 (d, *J* = 5.9 Hz, 1H), 5.21 (dd, *J* = 6.2, 3.1 Hz, 2H), 5.08 (td, *J* = 9.7, 4.0 Hz, 3H), 4.64–4.56 (m, 2H), 4.21 (m, 1H), 4.14 (d, *J* = 4.5 Hz, 1H), 4.09 (dt, *J* = 12.4, 3.0 Hz, 2H), 3.65 (ddd, *J* = 10.0, 4.3, 2.2 Hz, 2H), 3.15 (dd, *J* = 13.7, 7.6 Hz, 1H), 2.88 (dd, *J* = 13.7, 6.1 Hz, 1H), 2.05 (s, 3H), 2.03 (s, 3H), 2.02 (s, 3H), 2.00 (s, 3H), 1.99 (s, 3H),1.57 (s, 3H, *i*-propylidene C*H*_3_), 1.41 (s, 3H, *i*-propylidene C*H*_3_). ^13^C NMR (100 MHz, CDCl_3_) *δ* (ppm) 170.5, 170.2, 169.4, 169.3 (4C, 4 × Ac*C*O), 154.2 (1C, *C*_q_ adenine), 152.4 (2C, 2 × adenine *C*H) 148.3, 144.9 (3C, 3 × aromatic *C*_q_), 129.0, 127.9, 127.0 (15C, 15 × aromatic *C*H), 121.3 (1C, *C*_q_ adenine), 113.4 (1C, *i*-propylidene *C*_q_), 90.2, 85.0, 84.1, 83.6, 81.3, 76.0, 74.0, 69.9, 68.2 (9C, skeletal carbons), 71.4 (1C, N-Trt*C*_q_), 61.9 (1C, C-6’’), 28.3 (1C, C-5’), 26.4, 25.1 (2C, 2 × *i*-propylidene *C*H_3_), 20.7, 20.6 (4C, 4 × Ac*C*H_3_). MALDI-ToF MS: *m/z* calcd for C_46_H_49_N_5_NaO_12_S^+^ [M + Na]^+^ 918.299, found 918.297.

### 1-[2’,3’-*O*- Isopropylidene-5’-*S*-(β-d-glucopyranosyl)-5’-thio-α-l-lyxofuranosyl]-*N*-trityl-adenine (8)

Compound** 7** (150 mg, 0.166 mmol) was dissolved in dry MeOH (2.5 mL), then the pH of the solution was adjusted to approximately 10 and stirred overnight. Next day, the rection mixture was neutralized with Amberlite IR-120 (H^+^), filtered and evaporated under reduced pressure. The crude product was purified by flash chromatography (CHCl_3_/MeOH 95/5 → 9/1) to give compound **8** (119 mg, 98%, L-*lyxo* D-*ribo* ratio ~ 3:1) as a white solid. Rf = 0.36 (CH_2_Cl_2_/MeOH 9/1). ^1^H NMR (400 MHz, CDCl_3_) *δ* (ppm) 7.96 (s, 2H, H-2, H-8), 7.32 (d, *J* = 7.6 Hz, 8H, aromatic), 7.16 (dt, *J* = 22.4, 7.2 Hz, 14H, aromatic), 5.99 (s, 1H, H-1’), 5.55 (br. s, 1H, O*H*), 5.37 (d, *J* = 5.6 Hz, 1H, H-2’), 5.11–5.08 (m, 1H, H-3’), 4.52 (s, 1H), 4.33 (d, *J* = 9.3 Hz, 1H), 3.69–3.54 (m, 3H), 3.53–3.47 (m, 1H), 3.44–3.34 (m, 1H), 3.23 (s, 2H), 3.20–3.03 (m, 2H), 3.02–2.84 (m, 2H), 1.26 (s, 6H, 2 × *i*-propylidene C*H*_3_). ^13^C NMR (100 MHz, CDCl_3_) *δ* (ppm) 154.1, 148.4 (2C, 2 × adenine *C*_q_), 144.9 (3C, 3 × aromatic *C*_q_), 129.1, 128.0, 127.0 (15C, aromatic), 120.8 (1C, adenine C_q_), 113.4 (1C, *i*-propylidene *C*_q_), 90.0, 86.6, 85.0, 83.6, 81.1, 79.8, 77.9, 72.7, 69.3 (9C, skeletal carbons), 71.5 (1C, Trt-*C*_q_), 61.4 (1C, C-6’’), 29.2 (1C, C-5’), 26.3, 25.0 (2C, 2 × *i*-propylidene *C*H_3_). MALDI-ToF MS: *m/z* calcd for C_38_H_41_N_5_NaO_8_S [M + Na]^+^ 750.2574, found 750.2568.

### 1-[5’-*S*-(2,3,4,6-Tetra-*O*-acetyl-β-d-glucopyranosyl)-5’-thio-α-l-lyxofuranosyl]-adenine (9)

Compound **7** (258 mg, 0.288 mmol) was dissolved in 90% aq. TFA solution (5 mL), then Et_3_SiH (149 µl, 0.864 mmol, 3.0 equiv.) was added and stirred for 2 h. the reaction mixture was diluted with toluene and evaporated under educed pressure. The crude product was purified by flash chromatography (CHCl_3_/MeOH 97/3 → 95/5 → 9/1) to give compound **9** (92 mg, 52%, D-*ribo*: L-*lyxo* ratio ~ 1:3) as a white solid. Rf = 0.74 (CH_2_Cl_2_/MeOH 85/15), MALDI-ToF MS: *m/z* calcs for C_24_H_31_N_5_NaO_12_S [M + Na]^+^ 636.159, found 636.160.

### 1-[3’-Deoxy-3’-*C*-(2-naphtyl)methylsulfanylmethyl-2’,5’-di-*O*-(*tert*-butyldimethylsilyl)-β-D-xylofuranosyl]-uracil (11)

Compound **30** (200 mg, 0.426 mmol), naphthalen-2-ylmethanethiol **27c** (148 mg, 0.853 mmol, 2.0 equiv.) and DPAP (10.6 mg, 0.043 mmol, 0.1 equiv.) were dissolved in THF (1.5 mL) and irradiated for 4 × 15 min at − 40 °C. The solvent was evaporated under reduced pressure and the crude product was purified by flash column chromatography (hexane/acetone 9/1) to give compound **11** (165 mg, 61%, with ~ 10:1 D-*xylo*:D-*ribo* ratio) as a colorless syrup. [α]_d_ =  + 39.2 (c = 0.12, CHCl_3_), Rf = 0.20 (hexane/acetone 8/2), ^1^H NMR (400 MHz, CDCl_3_) *δ* (ppm) 7.94 (d, *J* = 8.2 Hz, 1H, H-6), 7.87–7.74 (m, 4H, aromatic), 7.68 (s, 1H, aromatic), 7.50–7.45 (m, 2H, aromatic), 5.93 (d, *J* = 6.7 Hz, 1H, H-1’), 5.68 (dd, *J* = 8.1, 2.0 Hz, 1H, H-5), 4.29 (d, *J* = 7.4 Hz, 1H, H-4’), 4.10–4.05 (m, 1H, H-2’), 3.97 (dd, *J* = 11.8, 1.0 Hz, 1H, H-5’a), 3.89 (d, *J* = 3.0 Hz, 2H, naphthylmethylene C*H*_2_), 3.85 (dd, *J* = 11.8, 2.2 Hz, 1H, H-5’b), 2.72–2.59 (m, 3H, H-3’ & SC*H*_2_), 0.89 (s, 9H, *t*-Bu), 0.69 (s, 9H, *t*-Bu), 0.08 (s, 3H, SiC*H*_3_), 0.05 (s, 3H, SiC*H*_3_), − 0.19 (s, 3H, SiC*H*_3_), − 0.22 (s, 3H, SiC*H*_3_). ^13^C NMR (100 MHz, CDCl_3_) *δ* (ppm) 163.4, 150.8 (2C, 2x*C*O), 140.5 (1C, C-6), 135.0, 133.3, 132.8 (3C, naphthalene C-4a, C-8a & C-7), 128.8, 127.7, 127.7, 127.4, 126.7, 126.3, 126.0 (7C, 7 × aromatic *C*H), 102.8 (1C, C-5), 87.6 (1C, C-1’), 79.3 (1C, C-4’), 77.4 (1C, C-2’), 63.7 (1C, C-5’), 46.2 (1C, C-3’), 36.9 (1C, naphthylmethylene *C*H_2_), 28.2 (1C, SCH_2_), 26.0, 25.4 (6C, 2 × SiC(*C*H_3_)_3_), 18.2, 17.6 (2C, 2 × *t*-Bu*C*_q_), − 4.8, − 5.5, − 5.7 (4C, 4 × Si*C*H_3_). MALDI-ToF MS: *m/z* calcd for C_33_H_50_N_2_NaO_5_SSi_2_ [M + Na]^+^ 665.2877, found 665.2872.

### 1-[3’-Deoxy-3’-*C*-(*N*-Fmoc-cysteine methyl ester)thiylmethyl-2’,5’-di-*O*-(*tert*-butyldimethylsilyl)-β-D-xylofuranosyl]-uracil (12)

Compound **30** (194 mg, 0.414 mmol), *N*-Fmoc-cysteine-methyl-esther **27d** (221 mg, 0.621 mmol, 1.5 equiv.) and DPAP (10.6 mg, 0.041 mmol, 0.1 equiv.) were dissolved in THF (2 mL) and irradiated for 4 × 15 min at − 80 °C. The solvent was evaporated under reduced pressure and the crude product was purified by flash column chromatography (hexane/acetone 9/1 → 85/15) to give compound **12** (219 mg, 64%, ~ 15:1 D-*xylo*:D-*ribo* ratio) as a colorless syrup. [α]_d_ =  + 43.8 (c = 0.42, CHCl_3_), Rf = 0.19 (hexane/acetone 8/2), ^1^H NMR (400 MHz, CDCl_3_) *δ* (ppm) 9.73 (s, 1H, uracil N*H*), 7.97 (d, *J* = 8.1 Hz, 1H, H-6), 7.77 (d, *J* = 7.5 Hz, 2H, 2xFmoc ArC*H*), 7.63 (d, *J* = 7.4 Hz, 2H, 2xFmoc ArC*H*), 7.41 (t, *J* = 7.4 Hz, 2H, 2 × Fmoc ArC*H*), 7.33 (t, *J* = 7.4 Hz, 2H, 2 × Fmoc ArC*H*), 5.98 (d, *J* = 6.6 Hz, 1H, H-1’), 5.80 (d, *J* = 8.0 Hz, 1H, CysN*H*), 5.75 (d, *J* = 8.1 Hz, 1H, H-5), 4.65 (dd, *J* = 13.1, 5.5 Hz, 1H, H-α), 4.45 (dt, *J* = 13.2, 6.7 Hz, 1H, Fmoc C*H*_2a_), 4.38 (dd, *J* = 10.5, 7.1 Hz, 1H, Fmoc C*H*_2b_), 4.32–4.26 (m, 1H, H-4’), 4.24 (d, *J* = 7.0 Hz, 1H, fluorene H-9), 4.15 (dd, *J* = 9.1, 6.7 Hz, 1H, H-2’), 3.94 (d, *J* = 11.7 Hz, 1H, H-5’a), 3.87 (dd, *J* = 11.8, 1.6 Hz, 1H, H-5’b), 3.81 (s, 3H, COOC*H*_3_), 3.10 (dd, *J* = 13.4, 4.9 Hz, 1H, H-βa), 2.99 (dd, *J* = 13.3, 5.7 Hz, 1H, H-βb), 2.80 (dd, *J* = 14.7, 7.7 Hz, 2H, SC*H*_2_), 2.68 (dt, *J* = 15.2, 9.1 Hz, 1H, H-3’), 0.96 (s, 9H, *t*-Bu), 0.87 (s, 9H, *t*-Bu), 0.15 (s, 6H, 2xSiC*H*_3_), 0.02 (s, 3H, SiC*H*_3_), − 0.10 (s, 3H, SiC*H*_3_).^13^C NMR (100 MHz, CDCl_3_) *δ* (ppm) 171.1 (1C, *C*OOCH_3_), 163.5 (1C, C-4), 155.7 (1C, Fmoc *C*O), 150.8 (1C, C-2), 143.7, 141.3 (4C, 4 × Fmoc *C*_q_), 140.5 (1C, C-6), 127.8, 127.1, 125.1, 120.0 (8C, 8 × Fmoc aromatic *C*H), 102.8 (1C, C-5), 87.7 (1C, C-1’), 79.1 (1C, C-4’), 77.2 (1C, C-2’), 67.3 (1C, Fmoc *C*H_2_), 63.6 (1C, C-5’), 53.6, 52.9 (2C, COO*C*H_3_ & C-α), 47.1, 46.7 (2C, fluorene C-9 & C-3’), 35.4 (1C, C-β), 30.2 (1C, S*C*H_2_), 26.0, 25.5 (6C, 2 × SiC(*C*H_3_)_3_), 18.2, 17.7 (2C, 2 × *t*-Bu*C*_q_), − 4.6, − 4.73, − 5.5, − 5.7 (4C, 4xSi*C*H_3_). MALDI-ToF MS: *m/z* calcd for C_41_H_59_N_3_NaO_9_SSi_2_ [M + Na]^+^ 848.341, found 848.358.

### 1-[3’-Deoxy-3’-*C*-(2-sodium-sulfonatoethyl-sulfanylmethyl)-2’,5’-di-*O*-(*tert*-butyldimethylsilyl)-β-D-xylofuranosyl]-uracil (13)

Compound **30** (200 mg, 0.42 mmol), MesNa **27e** (91 mg, 0.56 mmol, 1.3 equiv.) and DPAP (10.6 mg, 0.042 mmol, 0.1 equiv.) were dissolved in a mixture of THF (1.7 mL) and MeOH (0.3 mL) and irradiated for 4 × 15 min at − 80 °C. The solvent was evaporated under reduced pressure and the crude product was purified by flash column chromatography (CHCl_3_/MeOH 95/5 → 9/1 → 8/2) to give compound **13** (253 mg, 80%, ~ 10:1 D-*xylo*:D-*ribo* ratio) as a white solid. [α]_d_ =  + 45.5 (c = 0.20, CHCl_3_), Rf = 0.58 (CH_2_Cl_2_/MeOH 8/2), ^1^H NMR (400 MHz, DMSO) *δ* (ppm) 7.80 (d, *J* = 8.1 Hz, 1H, H-6), 5.78 (d, *J* = 6.7 Hz, 1H, H-1’), 5.70 (d, *J* = 8.1 Hz, 1H, H-5), 4.24 (d, *J* = 7.6 Hz, 1H, H-4’), 4.09 (t, *J* = 7.6 Hz, 1H, H-2’), 3.87 (dd, *J* = 11.8, 1.7 Hz, 1H, H-5’a), 3.82 (dd, *J* = 11.8, 1.9 Hz, 1H, H-5’b), 2.81–2.73 (m, 2H, SC*H*_2_), 2.72–2.61 (m, 5H, H-3’, C*H*_2_SO_3_Na, SC*H*_2_), 0.91 (s, 9H, *t*-Bu), 0.82 (s, 9H, *t*-Bu), 0.11 (s, 6H, 2 × SiC*H*_3_), 0.01 (s, 3H, SiC*H*_3_), -0.14 (s, 3H, SiC*H*_3_). ^13^C NMR (100 MHz, DMSO) *δ* (ppm) 162.8, 150.7 (2C, 2 × *C*O), 139.7 (1C, C-6), 102.4 (1C, C-5), 86.8 (1C, C-1’), 78.6 (1C, C-4’), 77.0 (1C, C-2’), 63.2 (1C, C-5’), 51.5 (1C, CH_2_SO_3_Na), 45.6 (1C, C-3’), 28.4, 27.3 (2C, 2 × SCH_2_), 25.8, 25.5 (6C, 2xSiC(CH_3_)_3_), 17.9, 17.4 (2C, 2 × *t*-Bu*C*_q_), − 4.7, − 5.1, − 5.7, − 5.8 (4C, 4xSi*C*H_3_). MALDI-ToF MS: *m/z* calcd for C_24_H_45_N_2_Na_2_O_8_SSi_2_ [M + Na]^+^ 655.1951, found 655.1946.

### 1-[3’-Deoxy-3’-*C*-((2-acetamido-3,4,6-tri-*O*-acetyl-β-d-glucopyranosylthio)methyl)-2’,5’-di-*O*-(*tert*-butyldimethylsilyl)-β-d-xylofuranosyl]-5-uracil (14)

Compound **30** (100 mg, 0.21 mmol), *N*-acetyl-2-amino-3,4-di-*O*-acetyl-2-deoxy-1-thio-β-D-glucopyranose **27f** (90 mg, 0.25 mmol, 1.2 equiv.) and DPAP (5.3 mg, 0.021 mmol, 0.1 equiv.) were dissolved in a mixture of toluene (1 mL) and MeOH (0.5 mL) and irradiated for 4 × 15 min at − 80 °C. The solvent was evaporated under reduced pressure and the crude product was purified by flash column chromatography (CH_2_Cl_2_/acetone 8/2) to give compound **14** (148 mg, 85%, ~ 12:1 D-*xylo*:D-*ribo* ratio) as a white solid. [α]_d_ =  + 17.9 (c = 1.09, CHCl_3_), Rf = 0.25 (hexane/acetone 7/3) ^1^H NMR (400 MHz, CDCl_3_) *δ* (ppm) 7.92 (d, *J* = 8.1 Hz, 1H, H-6), 6.41 (d, *J* = 9.3 Hz, 1H, N*H*Ac), 5.87 (d, *J* = 6.7 Hz, 1H, H-1’), 5.66 (d, *J* = 8.0 Hz, 1H, H-5), 5.15 (t, *J* = 9.7 Hz, 1H, H-3’’), 4.94 (t, *J* = 9.7 Hz, 1H, H-4’’), 4.59 (d, *J* = 10.3 Hz, 1H, H-1’’), 4.29 (d, *J* = 5.8 Hz, 1H, H-4’), 4.13–3.97 (m, 4H, H-6’’ab, H-2’ and H-2’’), 3.83 (dd, *J* = 21.4, 11.4 Hz, 2H, H-5’ab), 3.67 (dt, *J* = 8.5, 4.7 Hz, 1H, H-5’’), 2.89 (d, *J* = 7.4 Hz, 1H, SCH_2_a), 2.85–2.74 (m, 2H, SCH_2_b & H-3’), 2.07 (s, 3H, AcC*H*_3_), 1.97 (s, 6H, 2 × AcC*H*_3_), 1.87 (s, 3H, AcC*H*_3_), 0.88 (s, 9H, *t*-Bu), 0.79 (s, 9H, *t*-Bu), 0.07 (s, 6H, 2 × SiC*H*_3_), − 0.04 (s, 3H, SiC*H*_3_), − 0.17 (s, 3H, SiC*H*_3_). ^13^C NMR (100 MHz, CDCl_3_) *δ* (ppm) 171.1, 170.7, 170.2, 169.3 (4C, 4xAc*C*O), 163.5, 150.8 (2C, 2 × uracil *C*O), 140.6 (1C, C-6), 102.7 (1C, C-5), 87.3 (1C, C-1’), 86.4 (1C, C-1″), 79.1 (1C, C-4’), 77.4 (1C, C-2’), 76.0 (1C, C-5″), 73.6 (1C, C-3″), 68.4 (1C, C-4″), 63.8 (1C, C-5’), 62.6 (1C, C-6″), 53.0 (1C, C-2″), 47.4 (1C, C-3’), 29.0 (1C, S*C*H_2_), 25.9, 25.5 (6C, 2 × SiC(*C*H_3_)_3_), 23.1, 20.7, 20.6, 20.5 (4C, 4xAc*C*H_3_), 18.1, 17.6 (2C, 2 × *t*-Bu*C*_q_), -4.6, -4.8, -5.5, -5.8 (4C, 4xSi*C*H_3_). MALDI-ToF MS: *m/z* calcd for C_36_H_61_N_3_NaO_13_SSi_2_ [M + Na]^+^ 854.3361, found 854.3356.

### 1-[3’-Deoxy-3’-*C*-(-2,3,4,6-tetra-*O*-acetyl-β-D-mannopyranosyl-sulfanylmethyl)-2’,5’-di-*O*-(*tert*-butyldimethylsilyl)-β-D-xylofuranosyl]-uracil (15)

Compound **30** (200 mg, 0.42 mmol), 2,3,4-tri-*O*-acetyl-1-thio-β-D-mannopyranose **27 g** (186 mg, 0.51 mmol, 1.2 equiv.) and DPAP (10.6 mg, 0.042 mmol, 0.1 equiv.) were dissolved in a mixture of toluene (1 mL) CH_2_Cl_2_ (1 mL) and MeOH (1 mL) and irradiated for 4 × 15 min at − 80 °C. The solvent was evaporated under reduced pressure and the crude product was purified by flash column chromatography (hexane/acetone 8/2 → 7/3) to give compound **15** (246 mg, 69%, ~ 50:1 D-*xylo*:D-*ribo* ratio) as a white solid. [α]_D_ =  + 2.73 (c = 0.11, CHCl_3_), Rf = 0.32 (hexane/acetone 7/3), ^1^H NMR (400 MHz, CDCl_3_) *δ* (ppm) 9.83 (s, 1H, N*H*), 7.89 (d, *J* = 8.1 Hz, 1H, H-6), 5.91 (d, *J* = 7.0 Hz, 1H, H-1’), 5.67 (dd, *J* = 8.1, 1.6 Hz, 1H, H-5), 5.52 (d, *J* = 3.4 Hz, 1H), 5.27 (s, 1H), 5.13 (d, *J* = 9.9 Hz, 1H, H-4’’), 5.05 (dd, *J* = 10.0, 3.4 Hz, 1H), 4.69 (s, 1H, H-1″), 4.38 (d, *J* = 7.4 Hz, 1H, H-4’), 4.16–4.11 (m, 2H, H-6″ab), 4.06 (dd, *J* = 8.9, 7.4 Hz, 1H, H-2’), 3.86 (q, *J* = 11.6 Hz, 2H, H-5’ab), 3.76–3.67 (m, 1H, H-5’’), 3.00 (d, *J* = 10.0 Hz, 1H), 2.82 (d, *J* = 12.1 Hz, 1H, SC*H*_2_a), 2.79–2.70 (m, 2H, SC*H*_2_b & H-3’), 2.15 (s, 3H, AcC*H*_3_), 2.11 (s, 3H, AcC*H*_3_), 2.01 (s, 3H, AcC*H*_3_), 1.93 (s, 3H, AcC*H*_3_), 0.89 (s, 9H, *t*-Bu), 0.82 (s, 9H, *t*-Bu), 0.08 (s, 6H, 2xSiC*H*_3_), − 0.05 (s, 3H, SiC*H*_3_), − 0.17 (s, 3H, SiC*H*_3_). ^13^C NMR (100 MHz, CDCl_3_) *δ* (ppm) 170.8, 170.0, 170.0, 169.6 (4C, 4 × Ac*C*O), 163.3, 150.9 (2C, 2 × uracil *C*O), 140.2 (1C, C-6), 103.0 (1C, C-5), 87.0 (1C, C-1’), 84.6 (1C, C-1’’), 78.7 (1C, C-4’), 77.1 (1C, C-2’), 76.7 (1C, C-5’’), 71.6, 70.5, 65.5 (1C, C-4’’), 63.9 (1C, C-5’), 62.9 (1C, C-6’’), 53.5, 47.3 (1C, C-3’), 30.4 (1C, S*C*H_2_), 25.9, 25.5 (6C, 2 × SiC(*C*H_3_)_3_), 20.6, 20.6, 20.6, 20.5 (4C, 4 × Ac*C*H_3_), 18.1, 17.7 (2C, 2 × *t*-BuC_q_), − 4.6, − 4.8, − 5.5, − 5.7 (4C, 4xSi*C*H_3_). MALDI-ToF MS: *m/z* calcd for C_36_H_60_N_2_NaO_14_SSi_2_ [M + Na]^+^ 855.3201, found 855.3194.

### 1-[3’-Deoxy-3’-*C*-(-2,3,4,6-tetra-*O*-acetyl-β-D-galactopyranosyl-sulfanylmethyl)-2’,5’-di-*O*-(*tert*-butyldimethylsilyl)-β-D-xylofuranosyl]-uracil (16)

Compound **30** (200 mg, 0.42 mmol), 2,3,4-tri-*O*-acetyl-1-thio-β-D-galactopyranose **27 h** (186 mg, 0.51 mmol, 1.2 equiv.) and DPAP (10.6 mg, 0.042 mmol, 0.1 equiv.) were dissolved in a mixture of toluene (1 mL) CH_2_Cl_2_ (1 mL) and MeOH (1 mL) and irradiated for 4 × 15 min at − 80 °C. The solvent was evaporated under reduced pressure and the crude product was purified by flash column chromatography (hexane/acetone 8/2 → 7/3) to give compound **16** (305 mg, 86%, ~ 18:1 D-*xylo*:D-*ribo* ratio) as a white foam. [α]_d_ =  + 32.9 (c = 0.17, CHCl_3_), Rf = 0.32 (hexane/acetone 7/3) ^1^H NMR (400 MHz, CDCl_3_) *δ* (ppm) 7.89 (d, *J* = 8.2 Hz, 1H, H-6), 5.89 (d, *J* = 6.8 Hz, 1H, H-1’), 5.66 (dd, *J* = 8.1, 1.9 Hz, 1H, H-5), 5.38 (d, *J* = 3.2 Hz, 1H, H-4’’), 5.13 (t, *J* = 9.9 Hz, 1H, H-2’’), 5.02 (dd, *J* = 10.0, 3.3 Hz, 1H, H-3’’), 4.44 (d, *J* = 9.9 Hz, 1H, H-1’’), 4.28 (d, *J* = 8.0 Hz, 1H, H-4’), 4.15–3.98 (m, 3H, H-2’ & H-6’’ab), 3.87 (ddd, *J* = 29.2, 12.3, 3.9 Hz, 3H, H-5’ab & H-5’’), 2.93 (dd, *J* = 12.4, 4.1 Hz, 1H, SC*H*_2_a), 2.82 (t, *J* = 11.9 Hz, 1H, SC*H*_2_b), 2.72 (ddd, *J* = 21.3, 11.1, 4.5 Hz, 1H, H-3’), 2.08 (s, 3H, AcC*H*_3_), 2.05 (s, 3H, AcC*H*_3_), 2.01 (s, 3H, AcC*H*_3_), 1.92 (s, 3H, AcC*H*_3_), 0.89 (s, 9H, *t-*Bu), 0.81 (s, 9H, *t-*Bu), 0.07 (s, 6H, 2xSiC*H*_3_), − 0.03 (s, 3H, SiC*H*_3_), − 0.17 (s, 3H, SiC*H*_3_). ^13^C NMR (100 MHz, CDCl_3_) *δ* (ppm)170.5, 170.0, 169.9, 169.4 (4C, 4 × Ac*C*O), 163.3, 150.8 (2C, 2 × uracil *C*O), 140.3 (1C, C-6), 102.8 (1C, C-5), 87.2 (1C, C-1’), 85.3 (1C, C-1’’), 78.9 (1C, C-4’), 77.3 (1C, C-2’), 74.8 (1C, C-5’’), 71.6 (1C, C-3’’), 67.4 (1C, C-4’’), 66.8 (1C, C-2’’), 63.7 (1C, C-5’), 62.0 (1C, C-6’’), 47.1 (1C, C-3’), 28.5 (1C, S*C*H_2_), 25.9, 25.5 (6C, 2 × SiC(*C*H_3_)_3_), 20.7, 20.6, 20.5, 20.5 (4C, 4 × Ac*C*H_3_), 18.1, 17.6 (2C, 2 × *t*-Bu*C*_q_), − 4.6, − 4.8, − 5.5, − 5.8 (4C, 4 × Si*C*H_3_). ESI-ToF–MS: *m/z* calcd for C_36_H_60_N_2_NaO_14_SSi_2_ [M + Na]^+^ 855.3201, found 855.3196.

### 1-[3’-Deoxy-3’-*C*-(-2,3,4-tri-*O*-acetyl-β-D-xylopyranosyl-sulfanylmethyl)-2’,5’-di-*O*-(*tert*-butyldimethylsilyl)-β-D-xylofuranosyl]-uracil (17)

Compound **30** (200 mg, 0.42 mmol), 2,3,4-tri-*O*-acetyl-1-thio-β-D-xylopyranose **27i** (151 mg, 0.51 mmol, 1.2 equiv.) and DPAP (10.6 mg, 0.042 mmol, 0.1 equiv.) were dissolved in a mixture of toluene (1 mL) and CH_2_Cl_2_ (1 mL) and irradiated for 4 × 15 min at − 80 °C. The solvent was evaporated under reduced pressure and the crude product was purified by flash column chromatography (hexane/acetone 9/1 → 8/2) to give compound **17** (286 mg, 93%, ~ 12:1 D-*xylo*:D-*ribo* ratio) as an amorphous solid. [α]_D_ =  + 4.4 (c = 0.25 CHCl_3_), Rf = 0.29 (hexane/acetone 8/2), ^1^H NMR (400 MHz, CDCl_3_) *δ* (ppm) 7.86 (d, *J* = 8.1 Hz, 1H, H-6), 5.84 (d, *J* = 6.2 Hz, 1H, H-1’), 5.64 (d, *J* = 8.1 Hz, 1H, H-5), 5.13 (t, *J* = 8.6 Hz, 1H, H-3’), 4.90–4.80 (m, 2H, H-2’’ & H-4’’), 4.43 (d, *J* = 8.8 Hz, 1H, H-1’’), 4.23 (d, *J* = 7.9 Hz, 1H, H-4’), 4.13 (dd, *J* = 11.5, 5.0 Hz, 1H, H-5’’a), 4.05 (dd, *J* = 8.3, 6.8 Hz, 1H, H-2’), 3.82 (s, 2H, H-5’ab), 3.38–3.28 (m, 1H, H-5’’b), 2.82 (s, 1H, SC*H*_2_a), 2.81 (s, 1H, SC*H*_2_b), 2.63–2.54 (m, 1H, H-3’), 1.97 (s, 9H, 3 × AcC*H*_3_), 0.88 (s, 9H, *t-*Bu), 0.78 (s, 9H, *t-*Bu), 0.06 (s, 6H, 2 × SiC*H*_3_), − 0.04 (s, 3H, SiC*H*_3_), − 0.17 (s, 3H, SiC*H*_3_). ^13^C NMR (100 MHz, CDCl_3_) *δ* (ppm) 169.8, 169.6, 169.1 (3C, 3 × Ac*C*O), 163.4, 150.8 (2C, 2 × uracil *C*O), 140.2 (1C, C-6), 102.6 (1C, C-5), 87.7 (1C, C-1’), 84.2 (1C, C-1’’), 79.1 (1C, C-4’), 77.4 (1C, C-2’), 72.0 (1C, C-3’’), 69.4, 68.4 (2C, C-2’’ & C-4’’), 65.5 (1C, C-5’’), 63.3 (1C, C-5’), 47.2 (1C, C-3’), 27.2 (1C, S*C*H_2_), 25.8, 25.4 (6C, 2 × SiC(*C*H_3_)_3_), 20.5 (3C, 3xAc*C*H_3_), 18.1, 17.5 (2C, 2 × *t*-Bu*C*_q_), − 4.8, − 4.9, − 5.7, − 5.8 (4C, 4 × Si*C*H_3_). MALDI-ToF MS: *m/z* calcd for C_33_H_56_N_2_NaO_12_SSi_2_ [M + Na]^+^ 783.299, found 783.296.

### 1-[3’-Deoxy-3’-*C*-((2-acetamido-3,4,6-tri-*O*-acetyl-β-d-glucopyranosylthio)methyl)-2’,5’-di-*O*-(*tert*-butyldimethylsilyl)-β-d-xylofuranosyl]-5-methyluracil (18)

Compound **31** (100 mg, 0.21 mmol), *N*-acetyl-2-amino-3,4-di-O-acetyl-2-deoxy-1-thio-β-D-glucopyranose **27f** (90 mg, 0.25 mmol, 1.2 equiv.) and DPAP (5.3 mg, 0.021 mmol, 0.1 equiv.) were dissolved in a mixture of toluene (1 mL) and MeOH (0.5 mL) and irradiated for 3 × 15 min at − 80 °C. The solvent was evaporated under reduced pressure and the crude product was purified by flash column chromatography (CH_2_Cl_2_/acetone 8/2) to give compound **18** (134 mg, 77%, ~ 25:1 D-xylo:D-ribo ratio) as a white solid. [α]_D_ =  + 14.0 (c = 0.10, CHCl_3_), Rf = 0.28 (CH_2_Cl_2_/acetone 9/1), ^1^H NMR (400 MHz, CDCl_3_) *δ* (ppm) 7.53 (d, *J* = 0.9 Hz, 1H, H-6), 5.95 (d, *J* = 9.3 Hz, 1H, N*H*Ac), 5.89 (d, *J* = 7.0 Hz, 1H, H-1’), 5.19 (t, *J* = 9.8 Hz, 1H, H-3’’), 5.01 (t, *J* = 9.7 Hz, 1H, H-4’’), 4.61 (d, *J* = 10.4 Hz, 1H, H-1’’), 4.33 (d, *J* = 8.2 Hz, 1H, H-4’), 4.16 (d, *J* = 2.5 Hz, 1HH-6’’a), 4.13 (d, *J* = 3.8 Hz, 1H, H-6’’b), 4.12–4.08 (m, 1H, H-2’), 4.05 (d, *J* = 9.6 Hz, 1H, H-2’’), 3.92 (dd, *J* = 11.9, 1.1 Hz, 1H, H-5’a), 3.86 (dd, *J* = 11.8, 1.8 Hz, 1H, H-5’b), 3.71 (ddd, *J* = 9.8, 5.5, 2.7 Hz, 1H, H-5’’), 2.94 (d, *J* = 1.6 Hz, 1H, SC*H*_2_a), 2.92 (s, 1H, SC*H*_2_b), 2.82 (dt, *J* = 17.7, 9.0 Hz, 1H, H-3’), 2.14 (s, 3H, OAcC*H*_3_), 2.04 (s, 6H, 2 × OAcC*H*_3_), 1.95, 1.94 (2 s, 2 × 3H, NHAcC*H*_3_ & thymine C*H*_3_), 0.96 (s, 9H, *t*-Bu), 0.85 (s, 9H, *t*-Bu), 0.15 (s, 3H, SiC*H*_3_), 0.14 (s, 3H, SiC*H*_3_), 0.01 (s, 3H, SiC*H*_3_), − 0.13 (s, 3H, SiC*H*_3_). ^13^C NMR (100 MHz, CDCl_3_) *δ* (ppm) 171.3, 171.0, 170.2, 169.5 (4C, 4 × Ac*C*O), 163.9, 151.0 (2C, C-2, C-4), 135.7 (1C, C-6), 111.4 (1C, C-5), 87.2 (1C, C-1’), 86.7 (1C, C-1’’), 78.7 (1C, C-4’), 76.8 (1C, C-2’), 76.2 (1C, C-5’’), 73.6 (1C, C-3’’), 68.4 (1C, C-4’’), 63.8 (1C, C5’), 62.7 (1C, C-6’’), 53.3 (1C, C-2’’), 47.4 (1C, C-3’), 29.3 (1C, S*C*H_2_), 26.2, 25.7 (6C, 2xSiC(*C*H_3_)_3_), 23.3 (1C, NH Ac*C*H_3_), 20.9, 20.8, 20.7 (3C, 3xOAc*C*H_3_), 18.4, 17.8 (2C, 2 × *t*-Bu*C*_q_), 12.4 (1C, thymine *C*H_3_), − 4.4, − 4.6, − 5.2, − 5.3 (4C, 4 × Si*C*H_3_), MALDI-ToF MS: *m/z* calcd for C_37_H_63_N_3_NaO_13_SSi_2_ [M + Na]^+^ 868.3518, found 868.3525.

### 2’-Deoxy-2’-C-butylsulfanylmethyl-3’,5’-*O*-(1,1,3,3-tetraisopropyldisiloxane-1,3-diyl)-β-D-arabinofuranosyl-uracil (20)

Compound **29** (200 mg, 0.41 mmol), *n*-BuSH **27b** (360 µL, 3.3 mmol, 8.0 equiv.) and DPAP (10.6 mg, 0.041 mmol, 0.1 equiv.) were dissolved in THF (1 mL) and irradiated for 4 × 15 min at 0 °C. The reaction mixture was concentrated under reduced pressure. The crude product was purified by flash column chromatography (hexane/acetone 9/1) to give compound **20**^19^ (164 mg, 69% with ~ 20:1 D-*arabino:*D*-ribo* ratio) as a colourless syrup. [α]_d_ =  + 16.2 (c = 0.26, CHCl_3_), Rf = 0.32 (hexane/acetone 8/2), ^1^H NMR (400 MHz, CDCl_3_) *δ* (ppm) 9.77 (s, 1H, N*H*), 7.62 (d, *J* = 8.1 Hz, 1H, H-6), 6.23 (d, *J* = 4.4 Hz, 1H, H-1’), 5.69 (dd, *J* = 8.1, 1.6 Hz, 1H, H-5), 4.35 (t, *J* = 8.8 Hz, 1H, H-3’), 4.11 (dd, *J* = 13.3, 1.8 Hz, 1H, H-5’a), 4.01 (dd, *J* = 13.1, 2.7 Hz, 1H, H-5’b), 3.75 (d, *J* = 8.1 Hz, 1H, H-4’), 2.89–2.81 (m, 2H, H-2’ & SC*H*_2a_), 2.57–2.50 (m, 1H, SC*H*_2b_), 2.41 (td, *J* = 7.2, 2.0 Hz, 2H, SC*H*_2_CH_2_CH_2_CH_3_), 1.47 (dt, *J* = 14.6, 7.4 Hz, 2H, CH_2_C*H*_2_CH_2_CH_3_), 1.35–1.29 (m, 2H, CH_2_CH_2_C*H*_2_CH_3_), 1.06 (dt, *J* = 11.3, 4.8 Hz, 28H, 8 × *i*-PrC*H*_3_ & 4 × *i*-PrC*H*), 0.86 (t, *J* = 7.3 Hz, 3H, CH_2_CH_2_CH_2_C*H*_3_). ^13^C NMR (100 MHz, CDCl_3_) *δ* (ppm) 163.9, 150.8 (2C, 2x*C*O), 102.0 (1C, C-5), 84.2 (2C, C-1’, C-4’), 60.4 (1C, C-5’), 49.3 (1C, C-2’), 33.2 (1C, S*C*H_2_CH_2_CH_2_CH_3_), 31.4 (1C, SCH_2_*C*H_2_CH_2_CH_3_), 29.0 (1C, S*C*H_2_), 21.9 (1C, CH_2_CH_2_*C*H_2_CH_3_), 17.5, 17.4, 17.3, 17.2, 17.1, 17.1 (8C, 8 × *i*-Pr*C*H_3_), 14.1, 13.7, 13.0, 12.8, 12.6 (5C, 4 × *i*-PrCH & CH_2_CH_2_CH_2_*C*H_3_). MALDI-ToF MS: *m/z* calcd for C_26_H_48_KN_2_O_6_SSi_2_ [M + K]^+^ 611.262, found 611.317.

### Parasite culture

*Pf*3D7 and *Pf*RKL-9 were cultured by following procedure reported by Trager and Jensen^33^. In brief, culture media used for parasite culture supplemented with the complete RPMI 1640, 0.2% NaHCO_3_, 0.5% AlbuMax II, 0.1 mM hypoxanthine, 25 mg/mL gentamicin at 37 °C in human O^+^ RBCs and maintained in mixed gas environment (5% O_2_, 5% CO_2_ and 90% N_2_). In order to examine the morphological changes in the parasite, a blood film must be prepared. A thin blood film was air-dried, fixed with methanol, and allowed to dry before staining. Blood films were stained with a 7.5% Giemsa solution (pH of 7.2) for 15 min. Light microscopy analysis was carried out to evaluate the inhibitory effect of compounds at different concentrations against *Pf*3D7 and *Pf*RKL-9.

### SYBR green I based fluorescence assay

SYBR green I based fluorescence assay was performed to measure the growth inhibition effect of the 26 compounds and further dose dependent assay against *Pf*3D7, and *Pf*RKL-9 (Indian field strain, source- NIMR, New Delhi, India) was carried out at different concentrations^34^. In brief, *Pf*3D7 and *Pf*RKL-9 parasitized erythrocytes (trophozoite stage with 1% parasitemia) were diluted with noninfected erythrocytes at a constant 2% hematocrit in culture medium. Parasitized erythrocytes were treated with different concentrations of compounds ranging from 1 µM to 10 µM, whereas DMSO worked as a control. Triplicate wells of parasitized erythrocytes treated with compounds were seeded in 96 well microtiter plate following incubation at 37 °C for one cycle of parasite growth. Growth inhibition of parasites by compounds was calculated by fluorescence intensity taken at 485 nm excitation and 530 nm emission spectrum. Growth inhibition (%) was calculated as follows: % Inhibition = [1 − % Parasitemia (Treatment)/% Parasitemia (Control)] *100. Data were expressed as mean ± SD.

### Cytotoxicity effect of compounds on normal human cells

Cytotoxicity against human embryonic kidney cell line (ATCC) was determined using MTT dye^35^. In order to measuring the cytotoxicity of the compounds we used MTT dye to detect live/dead cells. RAW cells were stored at 37 °C, 5% CO_2_ in 75 cm^3^ sterile culture flasks (Corning) in complete Dulbecco's Modified Eagle Medium (DMEM) culture medium (a) supplemented with 5% Fetal Bovine Serum** (**FBS), gentamicin (40 mg/mL), with changing the medium thrice a week. The monolayers were washed, counted, diluted in complete medium, distributed in 96-well microtiter plates (5000 cells/well), followed by incubation for another 24 h at 37 °C. The test compounds and DMSO were added in triplicate. After a 48 h incubation at 37 °C, the supernatant was removed and 100 µL of MTT solution in complete DMEM was added to each well, followed by 2 h incubation at 37 °C. The culture plates were read in a spectrophotometer (Tecan infinite M200, nanoquant, UK) with a 490 nm filter, and the cytotoxic concentrations were determined based on a dose response curve.

### Hemolytic activity

Human RBCs were from Rotary Blood Bank, Tughlakabad Institutional Area, New Delhi, India. Effect of compounds on RBCs was checked by hemolytic assay^36^. In brief, RBCs suspension with 10% (v/v) washed with 1 × Phosphate-buffered saline (PBS) (pH 7.4) and again resuspended in 1 × PBS. RBCs suspension was incubated with compounds **7**, **16, 17, 18** and **1** at different concentrations (0.5, 1, 5, 10 and 20 μM) for 1 h at 37 °C. Samples were centrifuged and supernatant was taken for absorbance at 415 nm. Triton X-100 with 1% (v/v) was used as a positive control. Experiment was done in triplicate.

### Mitochondrial membrane potential

Mitochondrial membrane potential of the malaria parasites was determined by using MitoProbe™ JC-1 assay as described previously^24^. In brief, trophozoite stage parasites (10–12% parasitemia) were treated with IC_50_ concentration of compounds **7**, **16, 17, 18** and **1** for 3 h at 37 °C in the CO_2_ incubator. Samples were washed with 1 × PBS by centrifugation at 500 × *g* for 2 min at RT. Freshly prepared JC-1 dye was added to each sample with working concentration of 5 µg/mL. Incubation was done for 30 min at 37 °C in a CO_2_ incubator. Cells were washed twice with 1 × PBS. Spectrofluorometer was used to observe mitochondrial membrane potential by measuring fluoroscence intensities from the parasite lysate at wavelength of excitation (520 nm) and emission (590 nm) respectively. In vivo cell imaging was done with the help of Olympus fluoview 3000 confocal microscopy. Live cell imaging was observed under a 100X objective (oil immersion) and band pass filter at 355–460 nm. Experiments were repeated thrice and only representative figures are shown (Fig. 6).

### In vivo antiplasmodial activity assay

Antiplasmodial activity of hit compound **7** was investigated in mice model with the help of protocol described previously^37^. Briefly, intra-peritoneal injection of 1 × 10^7^ *Plasmodium berghei* ANKA infected erythrocytes diluted with sterile 1  × PBS was injected into mice. In each group five mice were taken. Thin smears were made from the tail blood of mice to check the infection. Post infection, *P. berghei* infected mice administered intra-peritoneally with compound **7** with the dose of 50 mg/kg body weight, daily for seven consecutive days post infection. Mice treated with 1xPBS were kept as vehicle control. Thin blood smears were made from day 1 to day 7 to count the parasitemia and mice were observed for 21 days for calculating mean survival time. Data were calculated and presented as percentage increase in the parasitemia. All animal experiments were carried out in accordance with the standard approved procedures^36^. The study was approved by the Institutional Animal Ethics Committee (IAEC) of Jawaharlal Nehru University, New Delhi, India; and the Committee for the Purpose of Control and Supervision of Experiments on Animals (CPCSEA), Government of India.

### Statistical analysis

The data was analyzed as one-way analysis of variance (ANOVA) to determine significance between the mean values obtained for control and after treatment with compounds. Dunnett’s test was used to compare the treatment and control and statistical significance was set at P ≤ 0.01.

## Supplementary Information

Supplementary Information.

## Data Availability

The datasets used and/or analysed during the current study available from the corresponding author (A.B.) on reasonable request.
