# Supplementary material for: In vitro and in vivo antiplasmodial evaluation of sugar-modified nucleoside analogues
Source: Sci Rep. 2023 Jul 28;13:12228. doi: 10.1038/s41598-023-39541-4 (PMC10382589; doi:10.1038/s41598-023-39541-4)

**Supplementary information**

***In vitro* and *in vivo* antiplasmodial evaluation of sugar-modified nucleoside analogues**

Miklós Bege^1,2,3#^, Vigyasa Singh^4,5#^, Neha Sharma^6#^, Nóra Debreczeni^1^, Ilona Bereczki^1,7^, Poonam^8,9^, Pál, Herczegh^1^, Brijesh Rathi^6,9^*, Shailja Singh^4,^*, Anikó Borbás^1,7^*

^1^Department of Pharmaceutical Chemistry, University of Debrecen, 4032 Debrecen, Egyetem tér 1, Hungary

^2^Institute of Healthcare Industry, University of Debrecen, 4032, Debrecen, Nagyerdei körút 98, Hungary

^3^MTA-DE Molecular Recognition and Interaction Research Group, University of Debrecen, 4032 Debrecen, Egyetem tér 1, Hungary

^4^Special Centre for Molecular Medicine, Jawaharlal Nehru University, New Delhi, 110067, India

^5^Department of Pharmacology & Toxicology, College of Pharmacy, University of Arizona, Tucson, AZ 85721, USA

^6^Laboratory for Translational Chemistry and Drug Discovery, Department of Chemistry, Hansraj College, University of Delhi, India

^7^National Laboratory of Virology, University of Pécs, Ifjúság útja 20, H-7624 Pécs, Hungary

^8^Department of Chemistry, Miranda House, University of Delhi, Delhi-110007 India

^9^Delhi School of Public Health, Institution of Eminence (IoE), University of Delhi, Delhi-110007 India

#Authors contributed equally

**Table of contents**

[In vitro assays S2](#_Toc118987301)

[HPLC-MS of compounds 1, 7, 16, 17 and 18 S4](#_Toc118987302)

[NMR spectra of the compounds S10](#_Toc118987303)

# In vitro assays

**Figure S1**: **(A-B).** Estimation of *in vitro* growth inhibition of 26 compounds against *Pf* 3D7 strain. Graph showing percent inhibition for the 26 compounds.

**Figure S2**: Estimation of *in vitro* half maximal inhibition concentration (IC_50_) of hit compounds **3** and **5** against *Pf* 3D7 and *Pf* RKL-9 strains **(A-D).**

**Figure S3.** Effect of compounds **3** and **5** on human red blood cells (RBCs). The hemolytic activity of the compounds on human RBCs was tested at 0.5, 1, 5, 10, 20µM concentration; absorbance was measured at 415 nm.

# HPLC-MS of compounds 1, 7, 16, 17 and 18

**
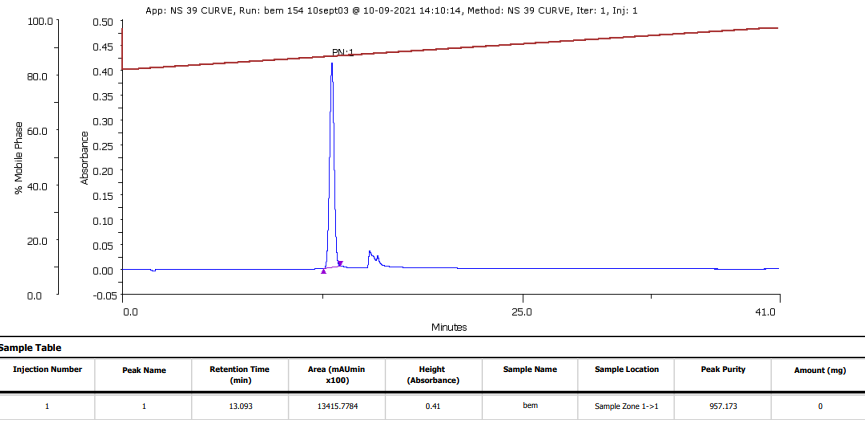
**

**Figure S4**: HPLC profile of compound **7** at 230 nm showing 95.7 % purity.

For analytical RP-HPLC a Waters 2695 Separations Module (Waters Corp., Milford, USA) was used. The separations were carried out on a VDSphere PUR 100 C18-M-SE, 5 μm, 150 x 4.6 mm column at an injection volume of 10 μL, using a flow rate of 1.0 mL/min with a Waters 2996 DAD set at 225 nm and a Bruker MicroTOF-Q type Qq-TOF MS instrument (Bruker Daltonik, Bremen, Germany) as detectors. The following system was used for the elution: Solvent A: methanol and Solvent B: H_2_O.

min A (%) B (%)

0.00 60.0 40.0

15.00 80.0 20.0

25.00 80.0 20.0

25.10 60.0 40.0

30.00 60.0 40.0

The MicroTOF-Q mass spectrometer was equipped with an electrospray ion source. The mass spectrometer was operated in positive ion mode with a capillary voltage of 3.5 kV, an endplate offset of −500 V, nebulizer pressure of 1.8 bar, and N_2_ as drying gas with a flow rate of 9.0 l/min at 200 °C. The mass spectra were recorded by means of a digitizer at a sampling rate of 2 GHz. The mass spectra were calibrated externally using the exact masses of clusters [(NaTFA)_n_+TFA]^+^ from the solution of sodium trifluoroacetate (NaTFA). The spectra were evaluated with the DataAnalysis 3.4 software from Bruker.


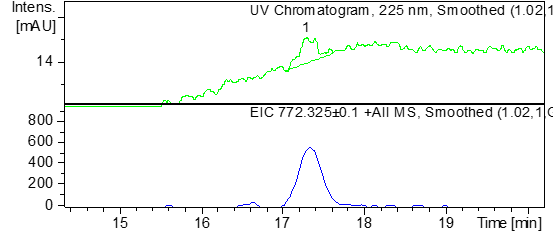

**Figure S5**: UV chromatogram, extracted ion chromatogram and MS spectrum of compound **1**


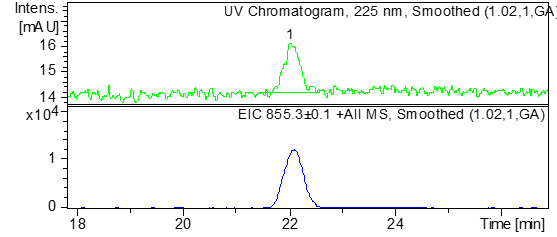

**Figure S6**: UV chromatogram, extracted ion chromatogram and MS spectrum of compound **16**


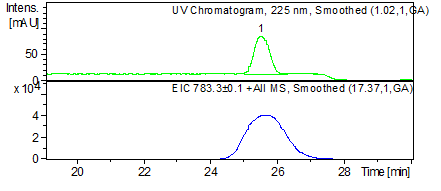

**Figure S7**: UV chromatogram, extracted ion chromatogram and MS spectrum of compound **17**


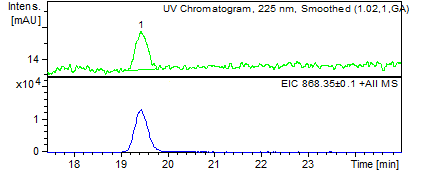

**Figure S8**: UV chromatogram, extracted ion chromatogram and MS spectrum of compound **18**

# NMR spectra of the compounds

Compound **7** (after single chromatographic purification)


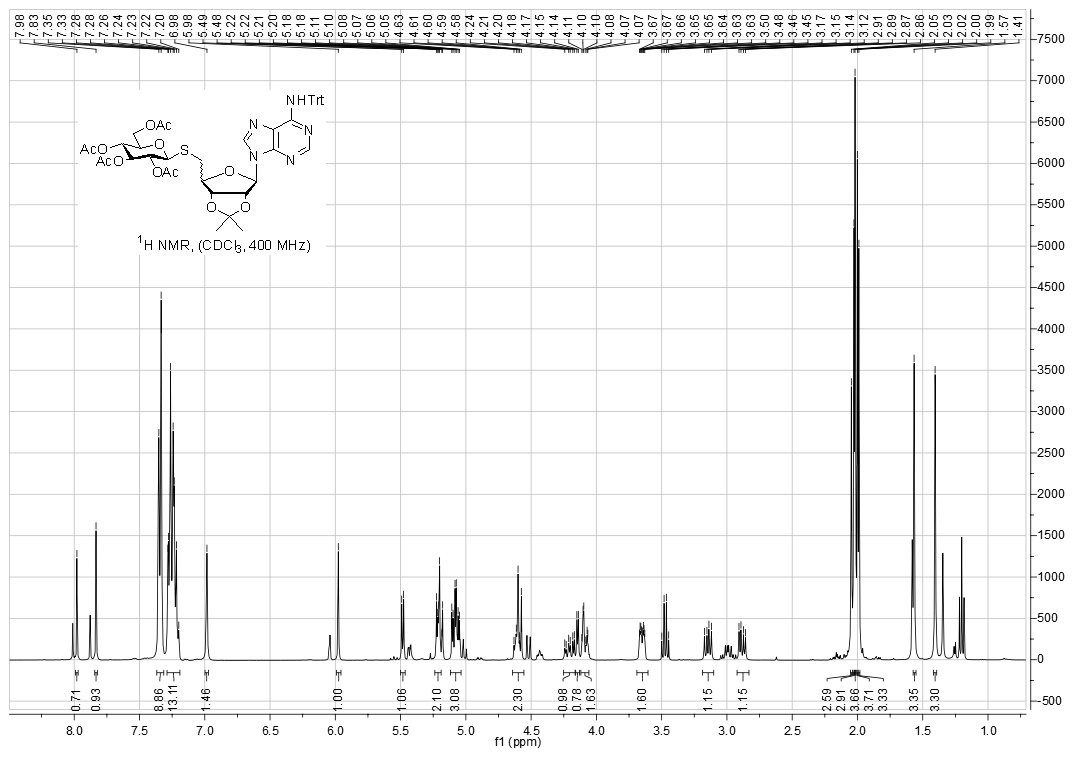


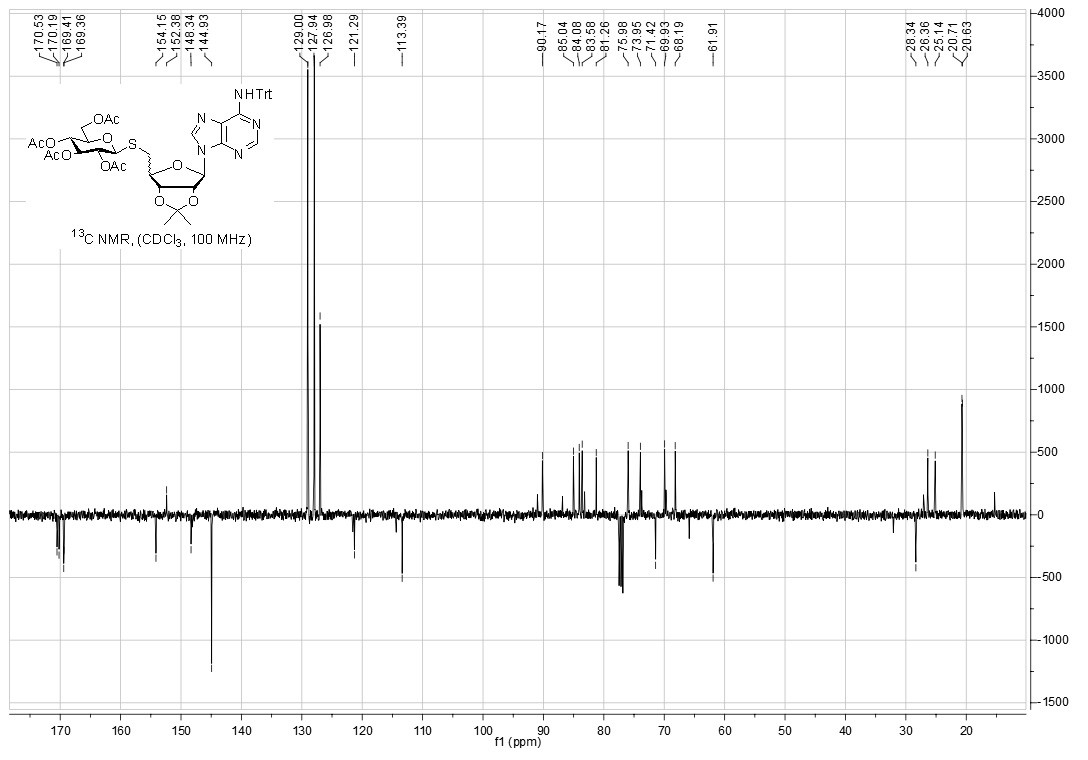


Determination of the diastereomeric ratio of compound **7** on the basis of the H-1’ signals of the D-*ribo* and L-l*yxo* isomers (L-*lyxo*. D *ribo* 3:1 after single chromatographic purification)


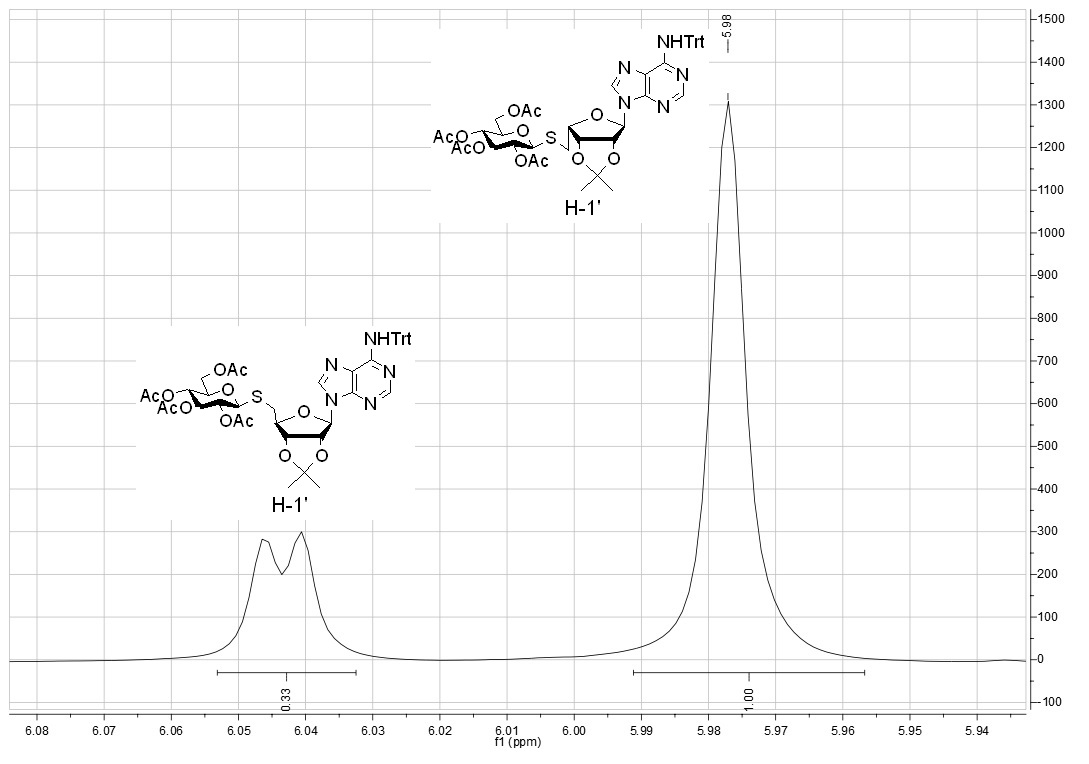


Compound **7** after second chromatographic purification


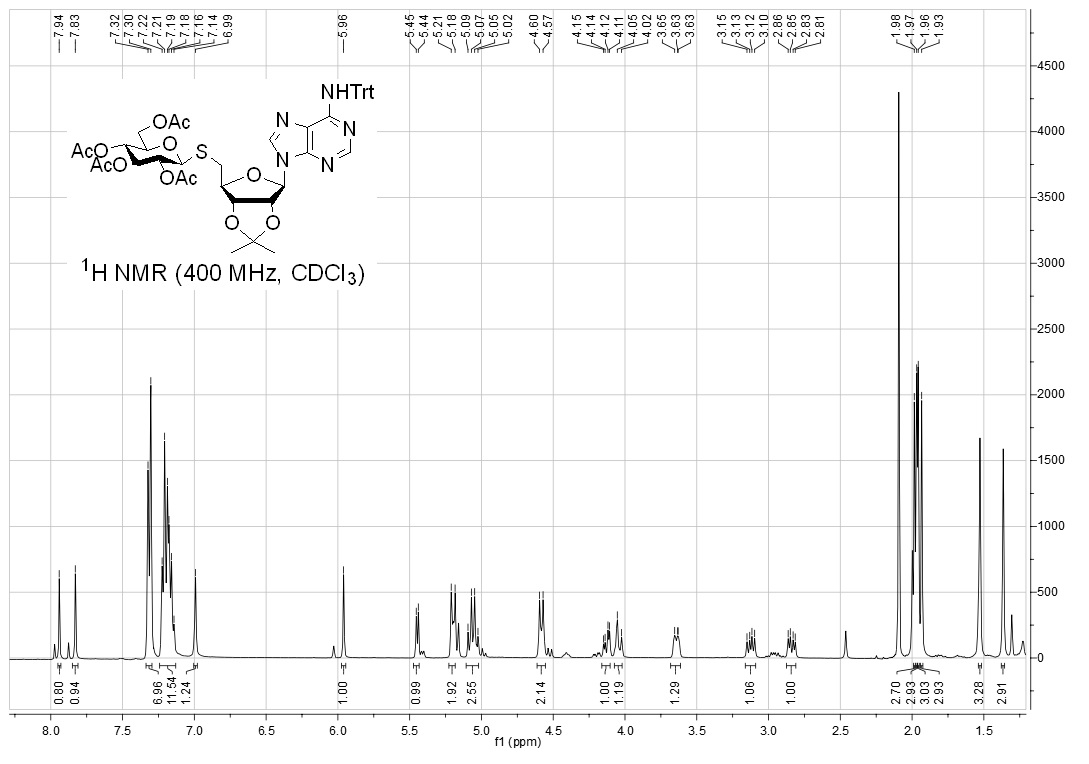


Determination of the diastereomeric ratio of compound **7** (L-*lyxo*. D *ribo* 6:1 after second chromatographic purification)


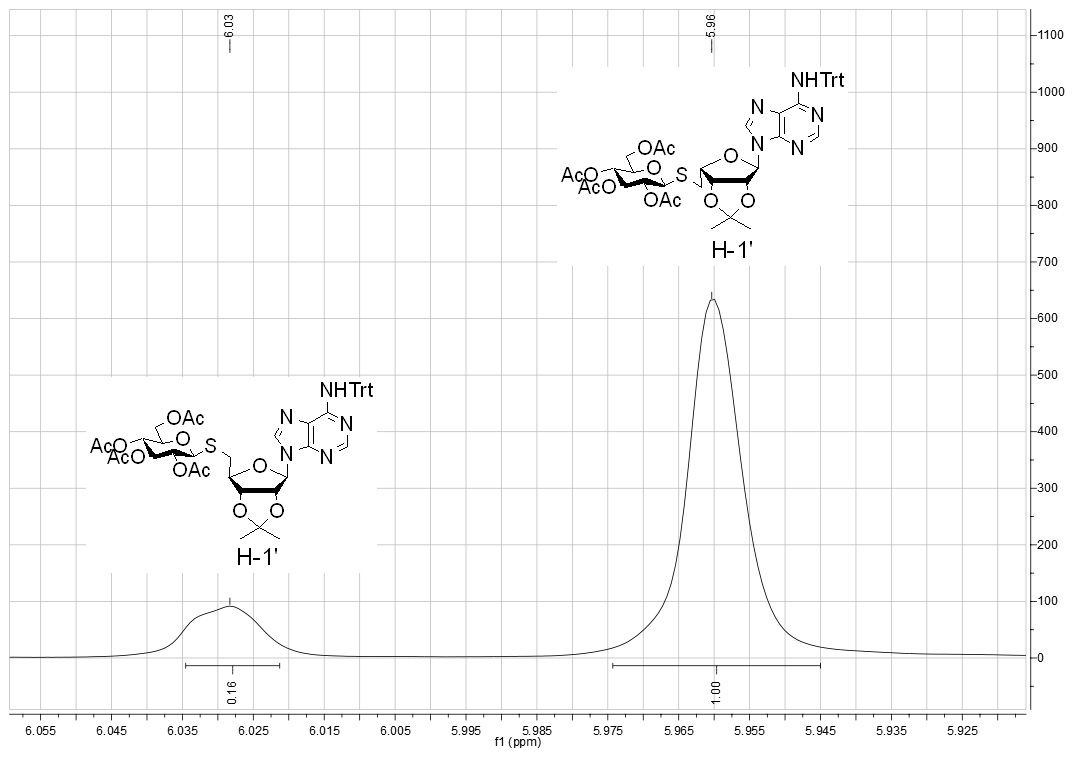


Compound **8**


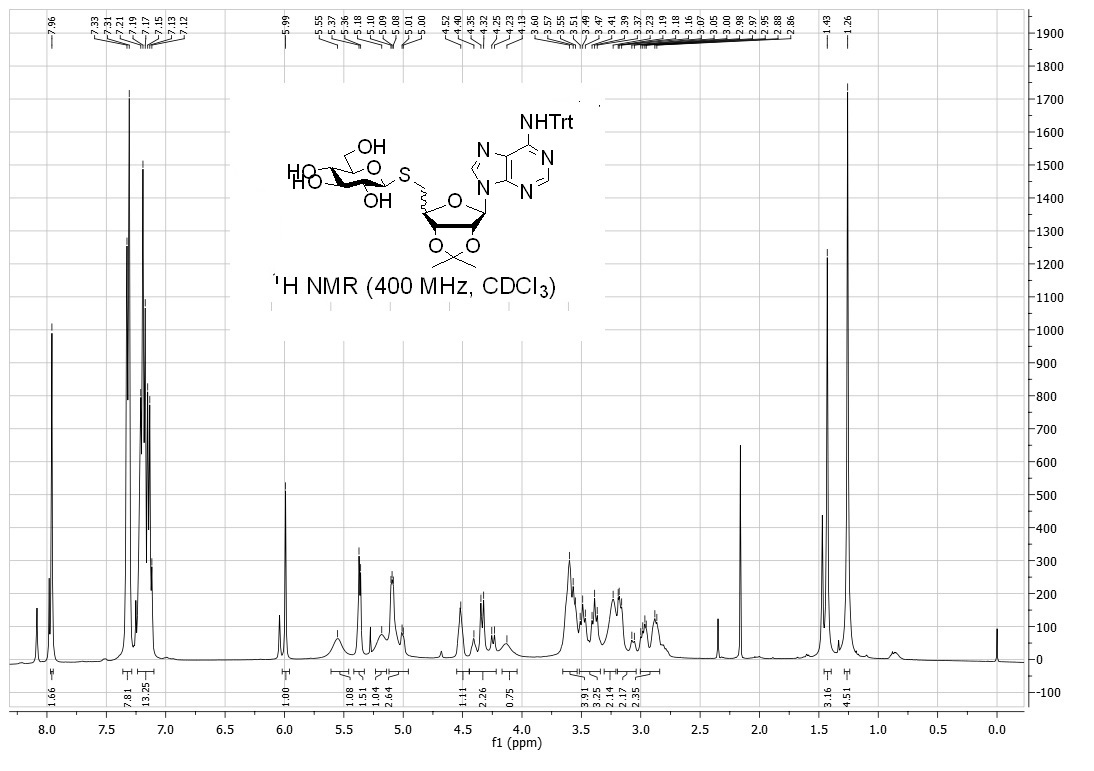


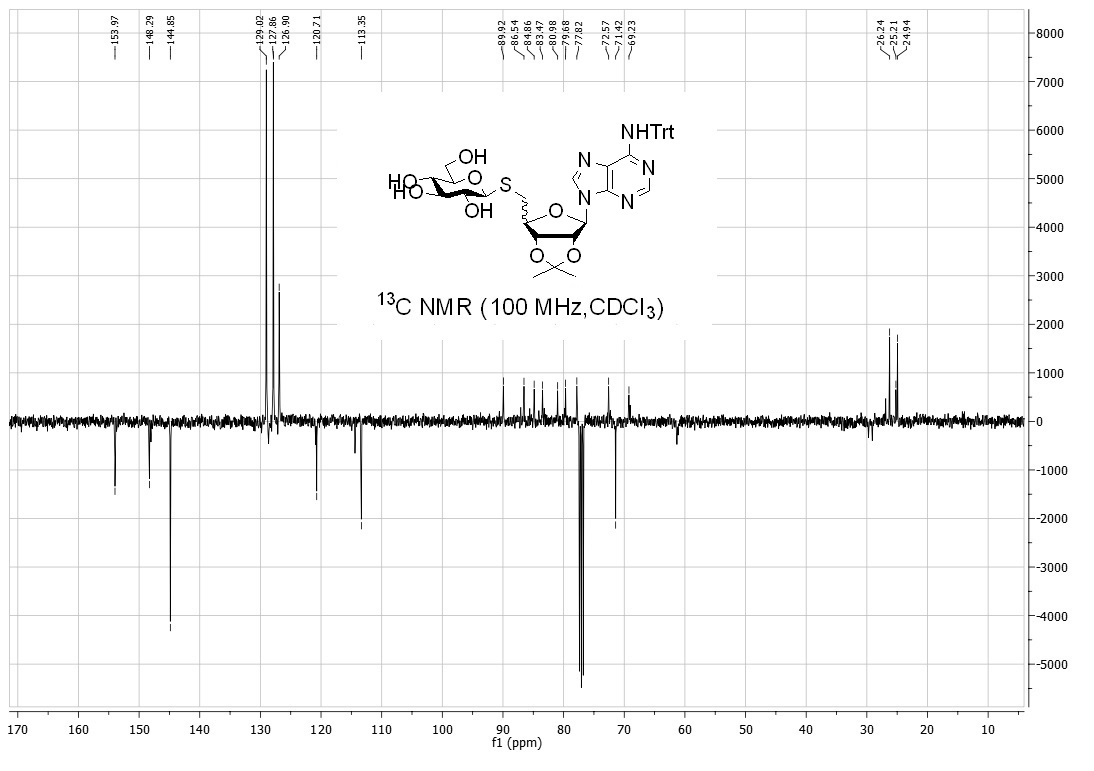


Compound **9**


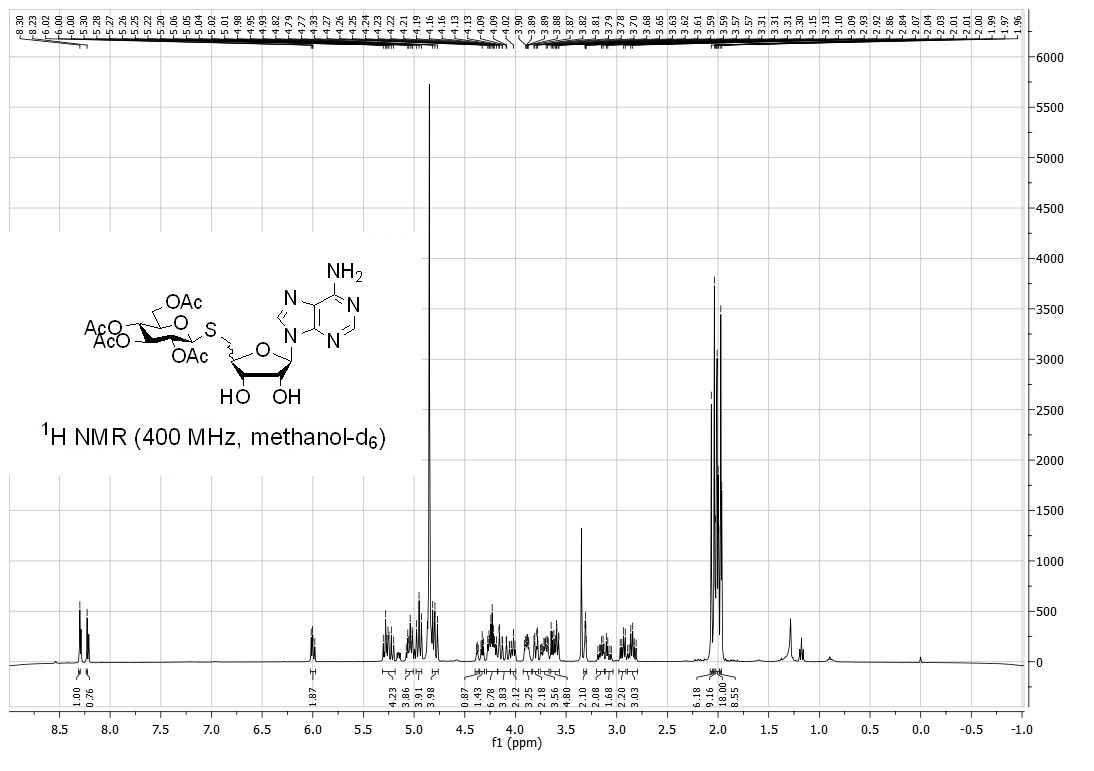


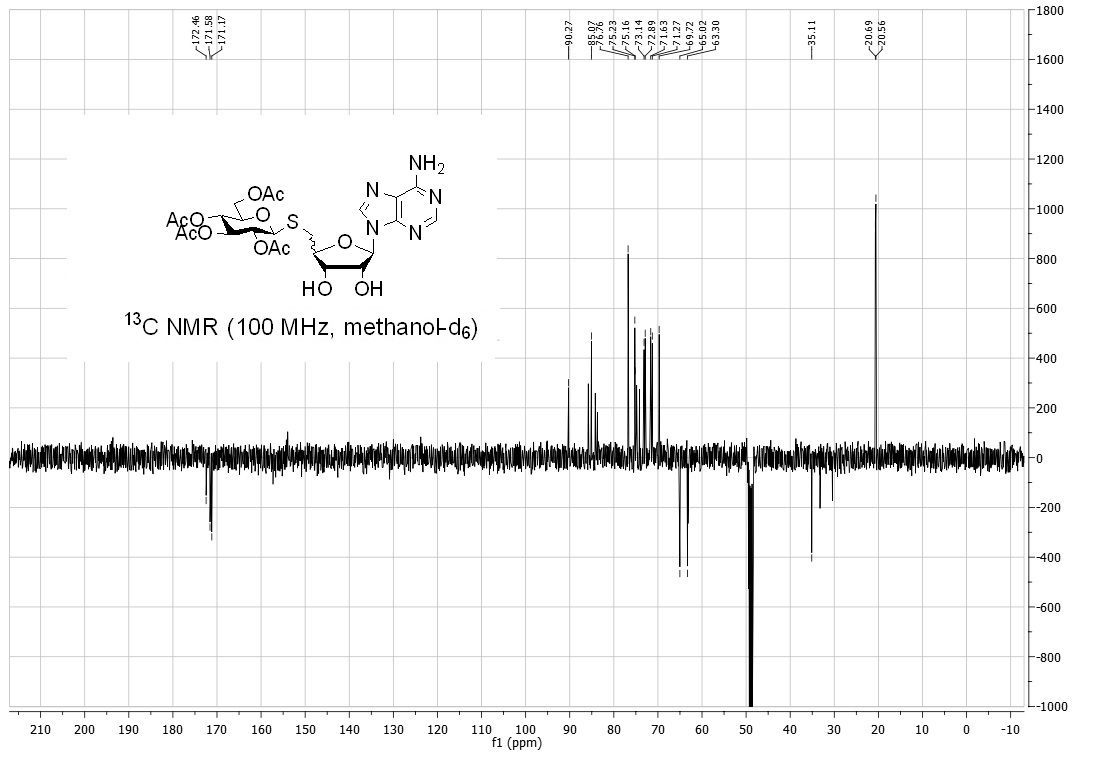


Compound **11**


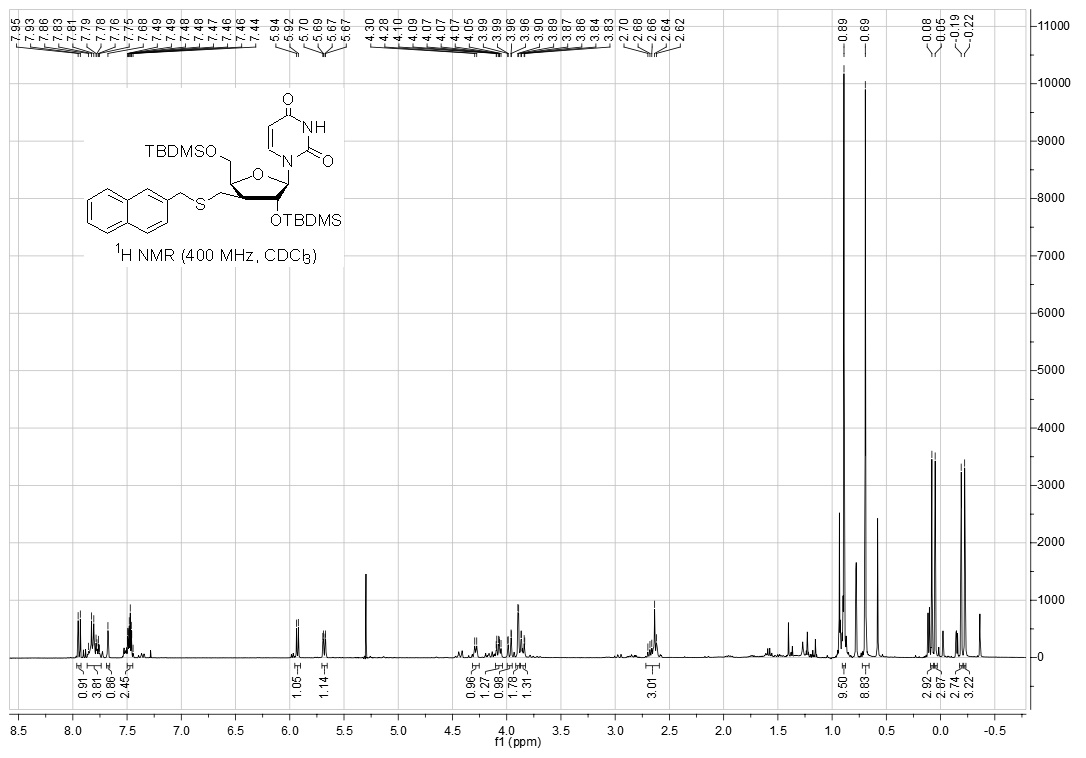


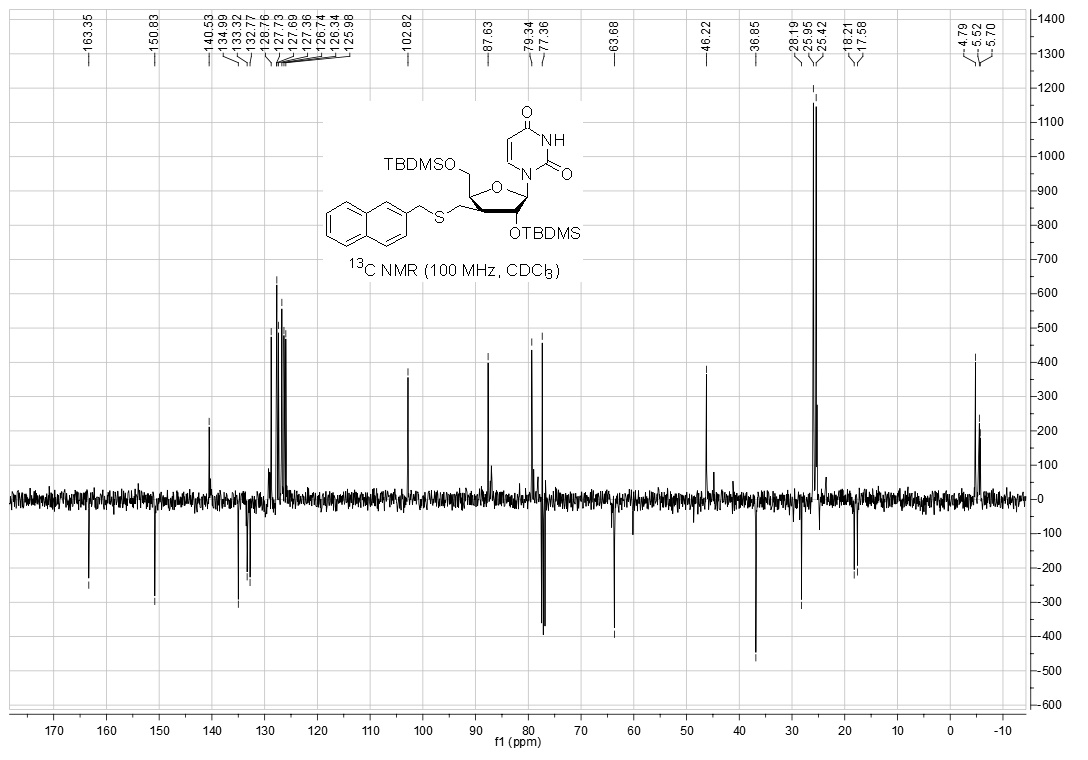


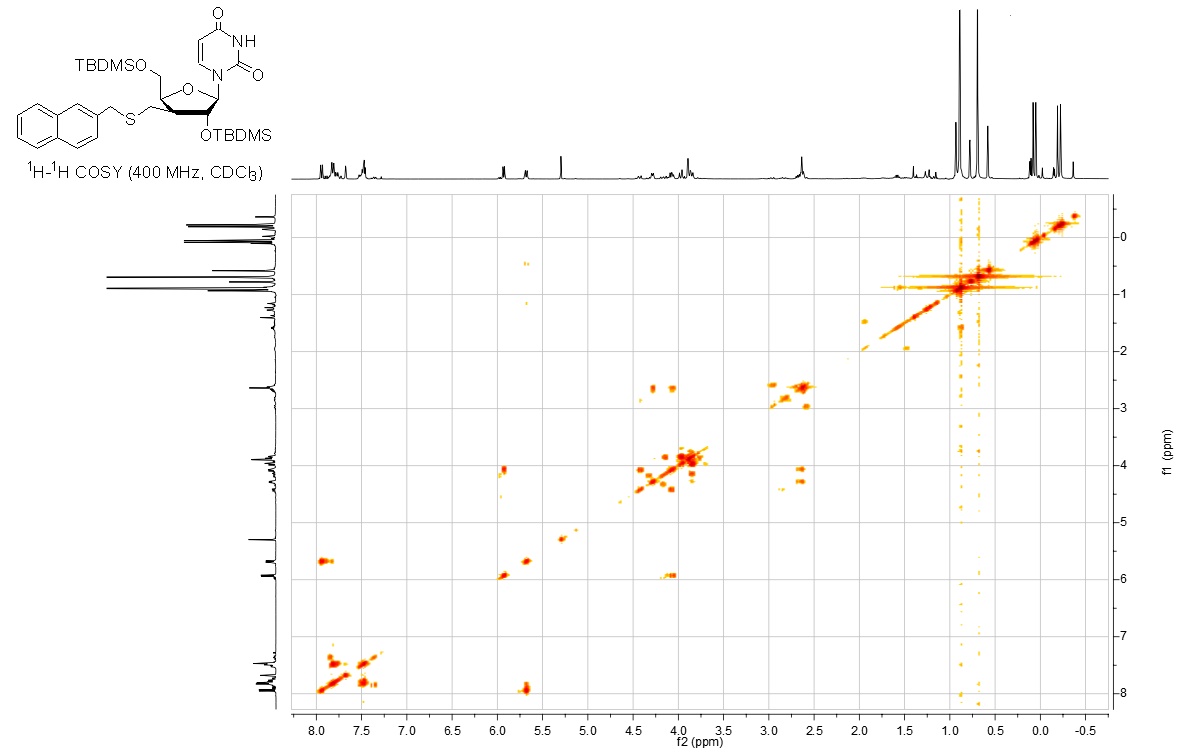


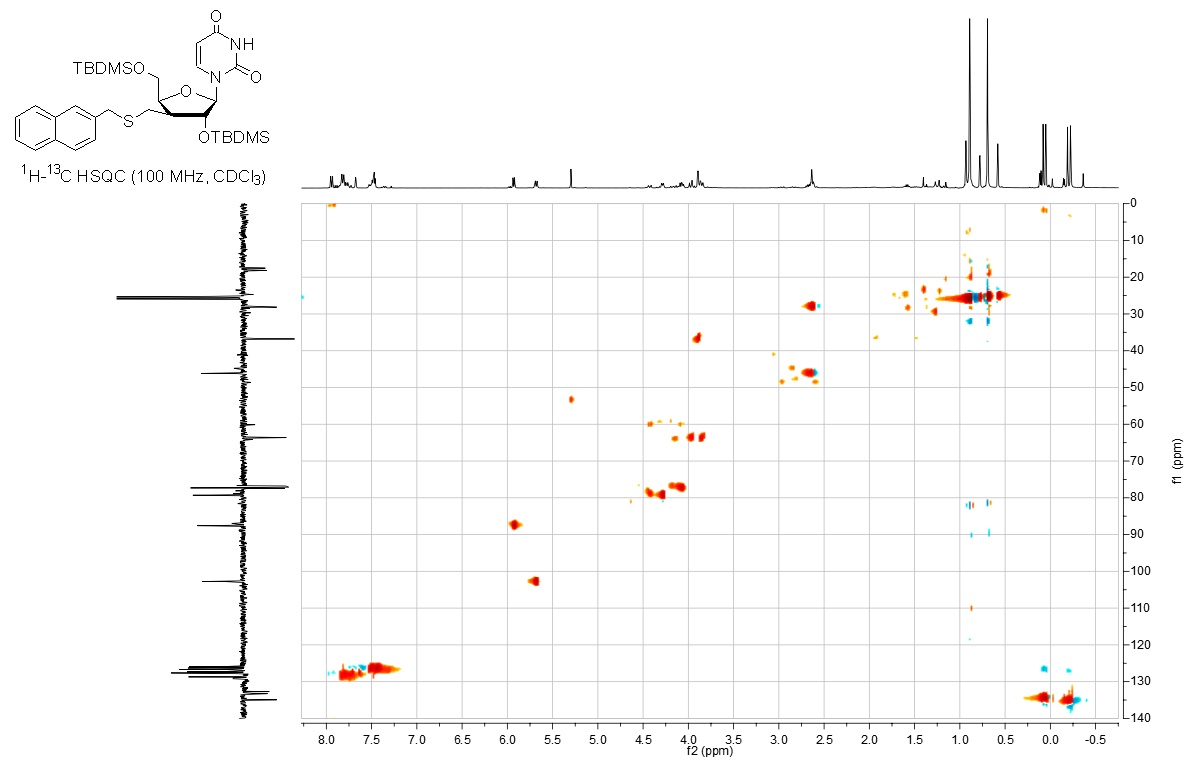


Compound **12**


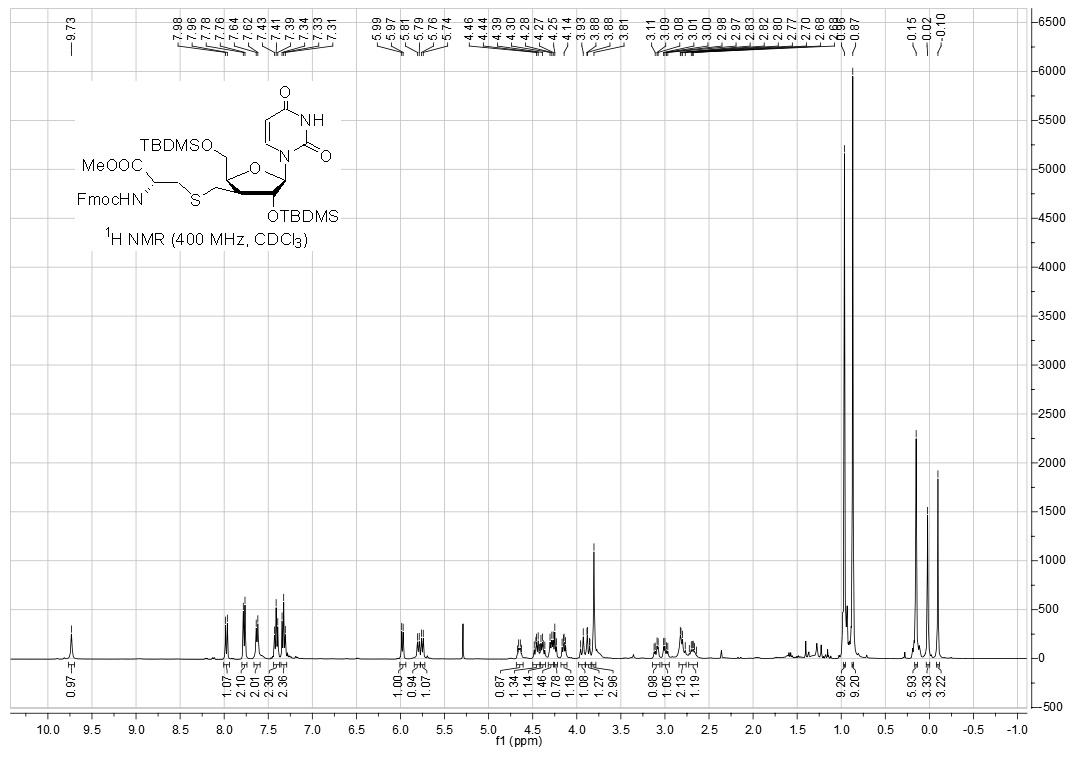


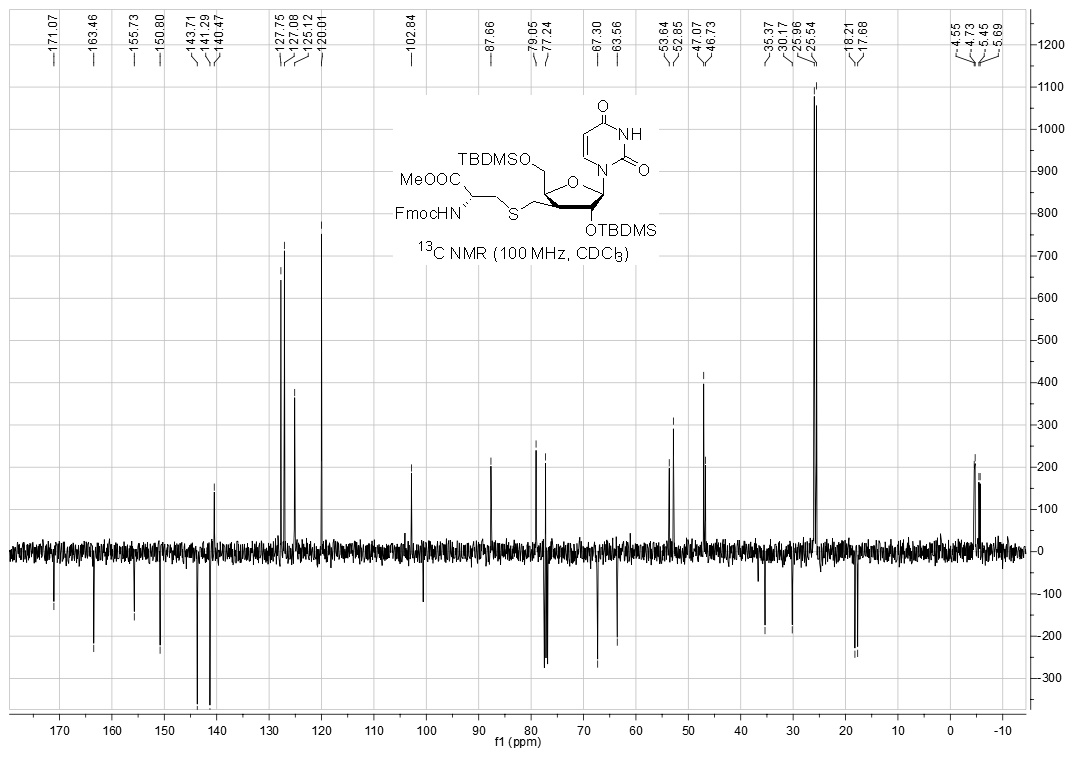


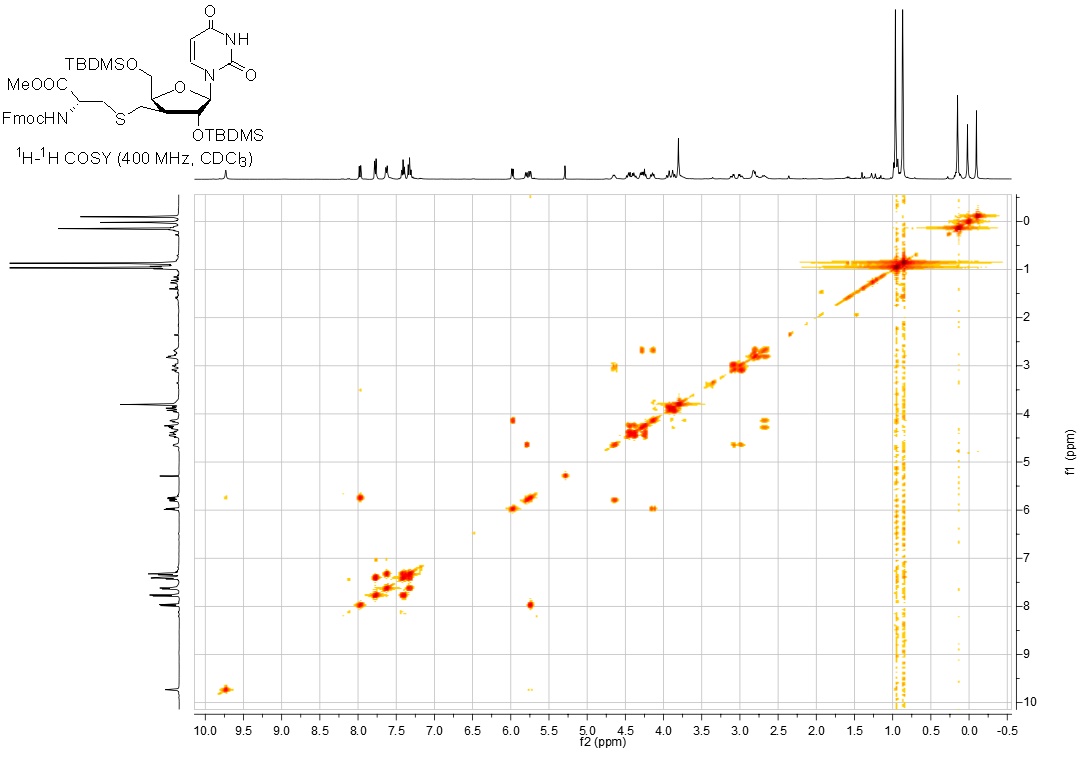


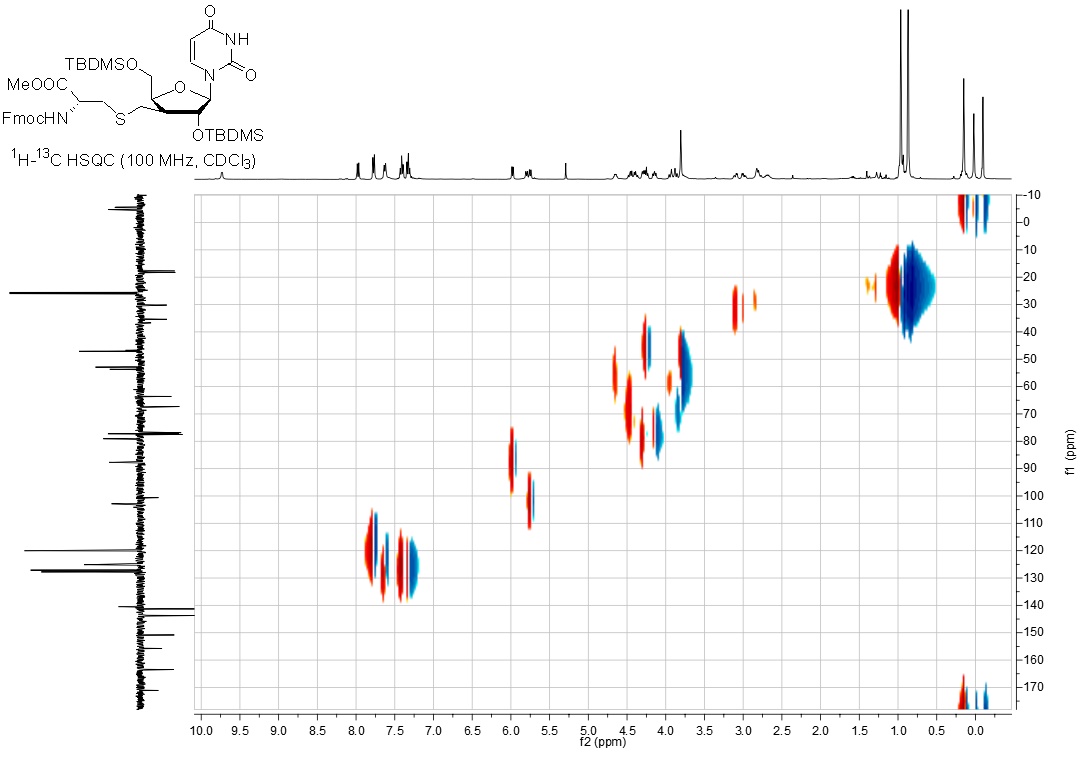


Compound **13**


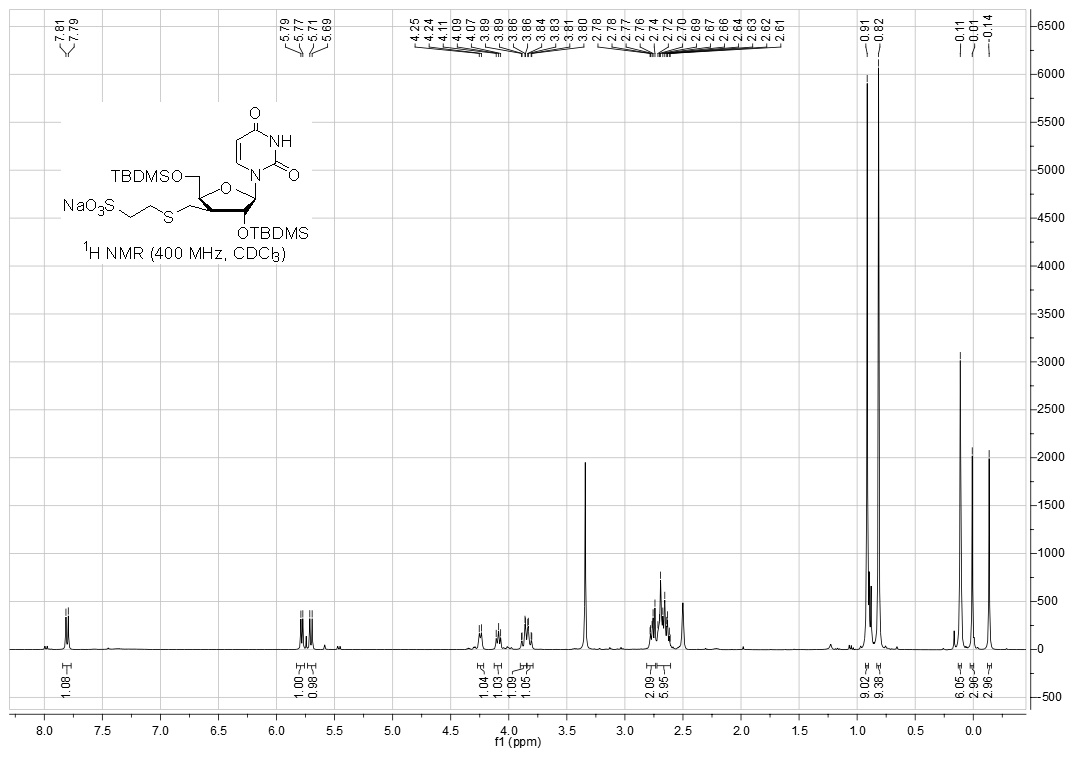


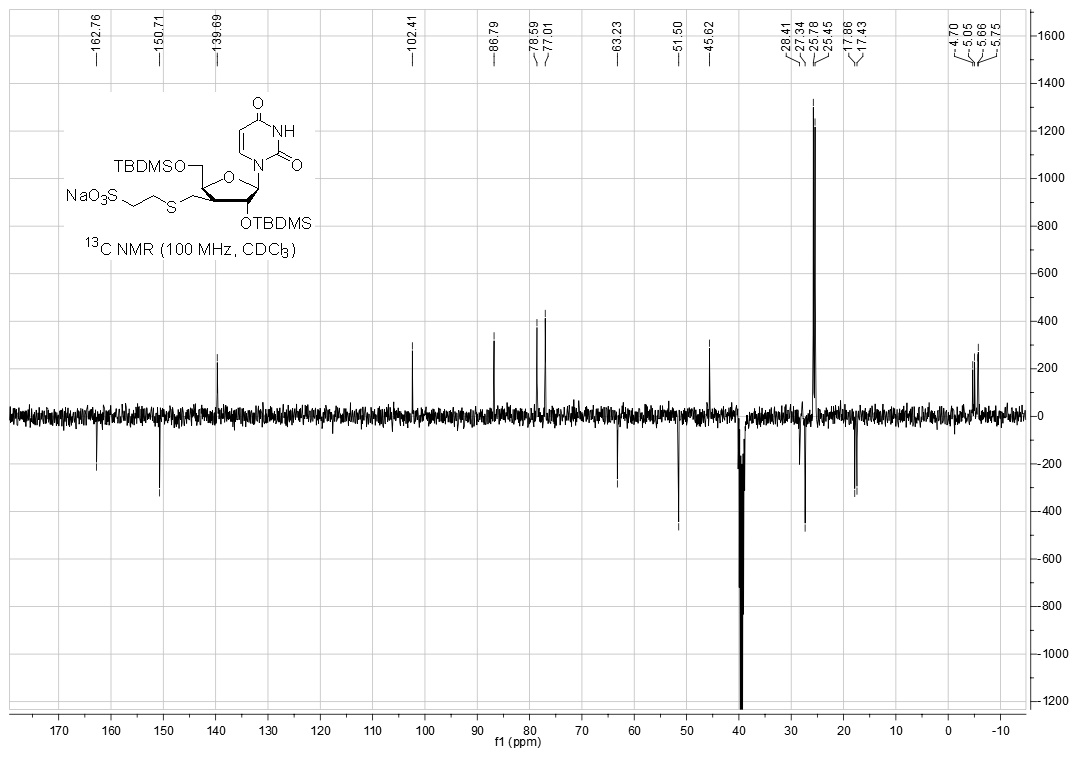


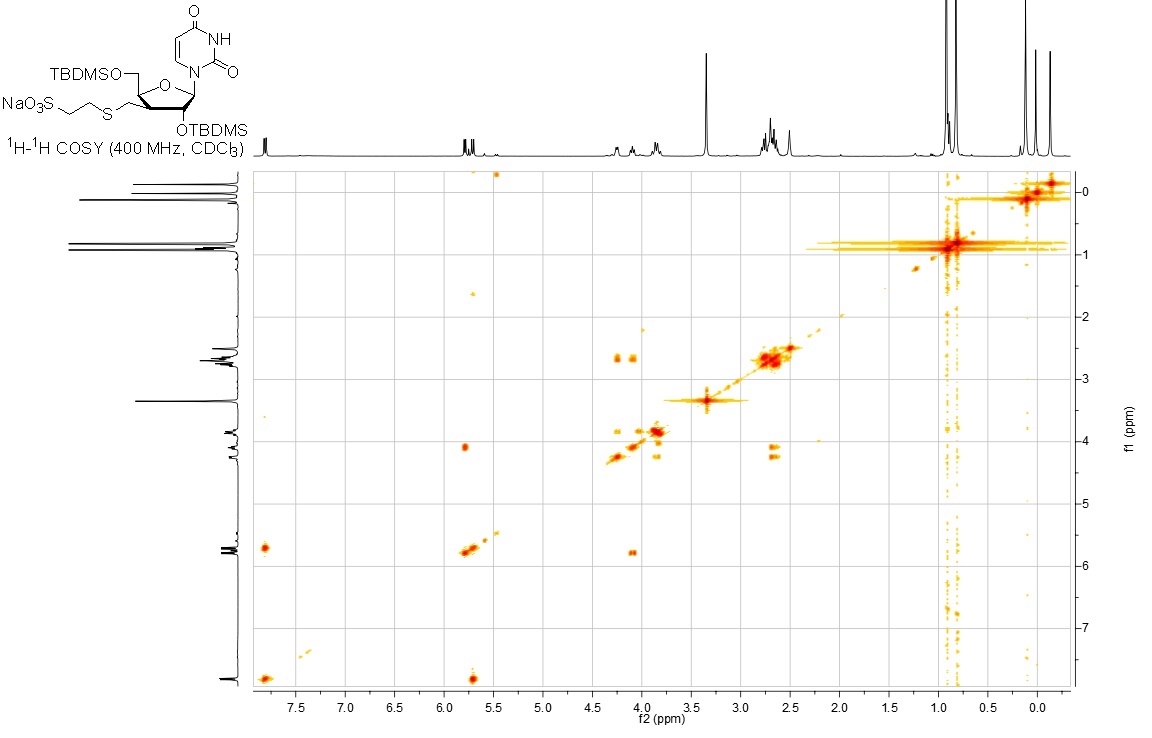


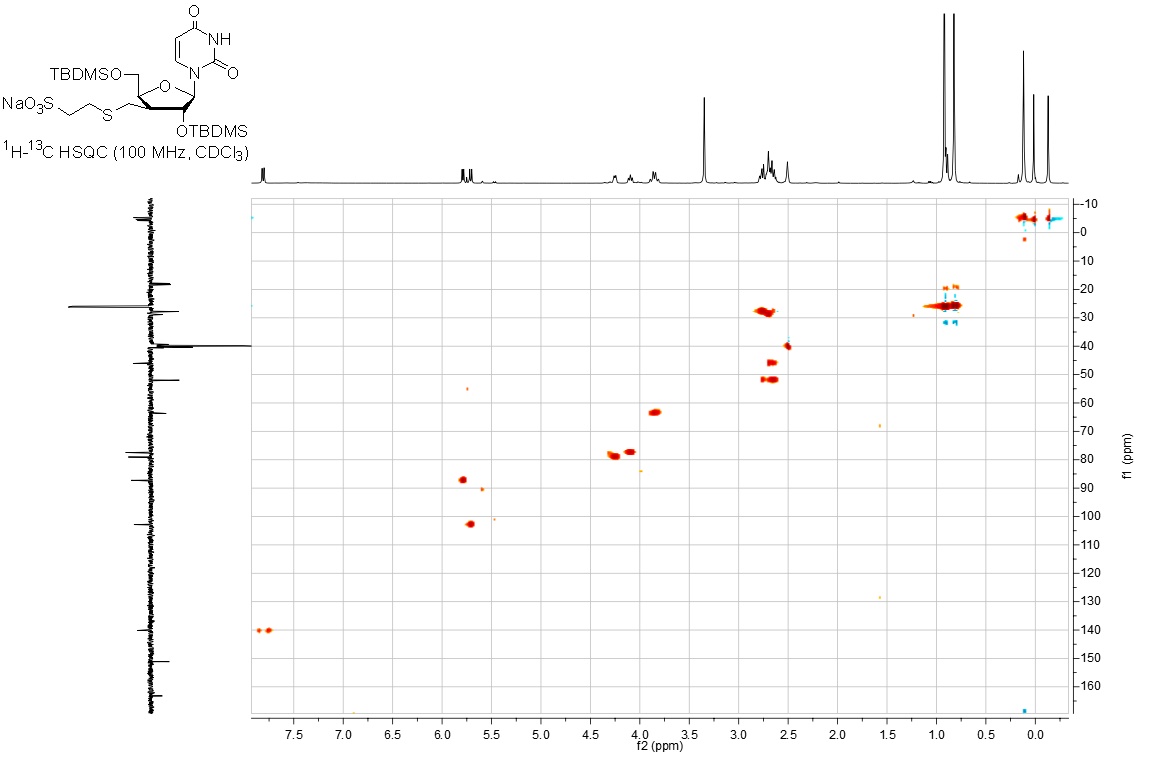


Compound **14**


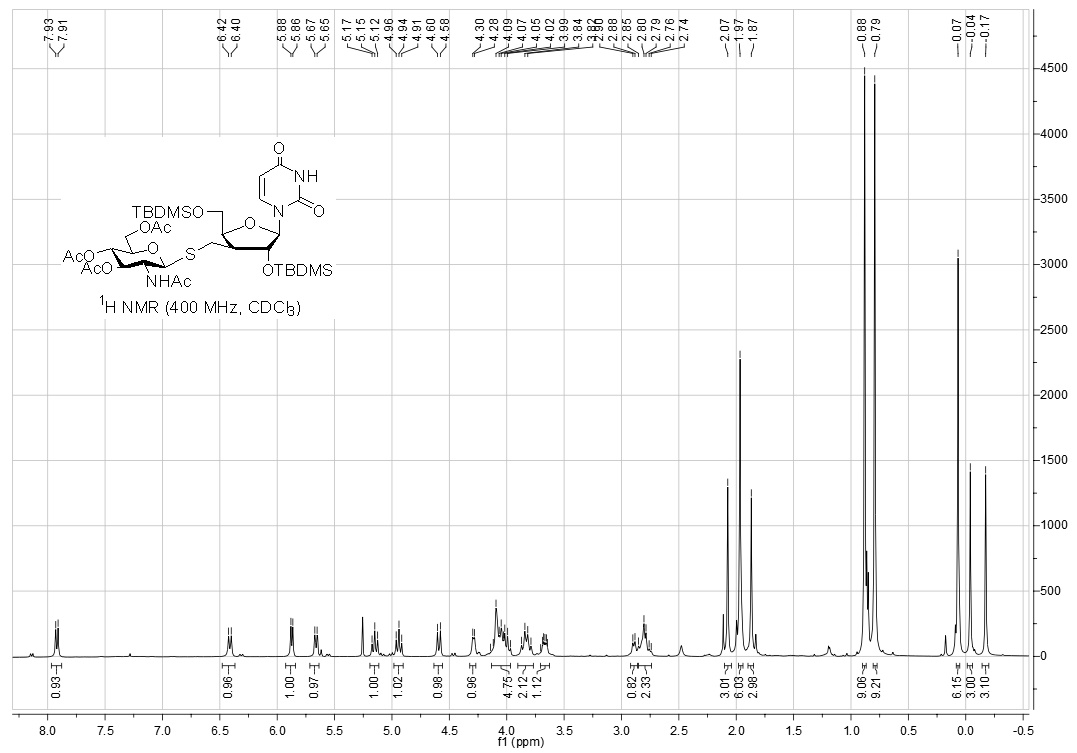


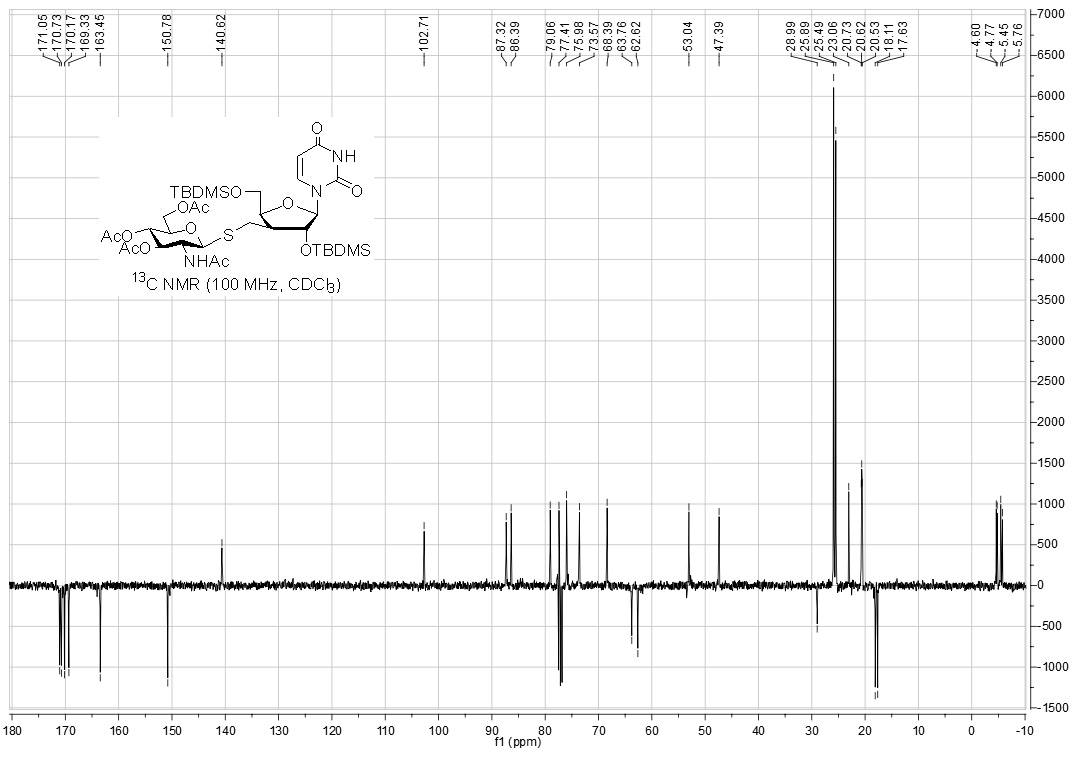


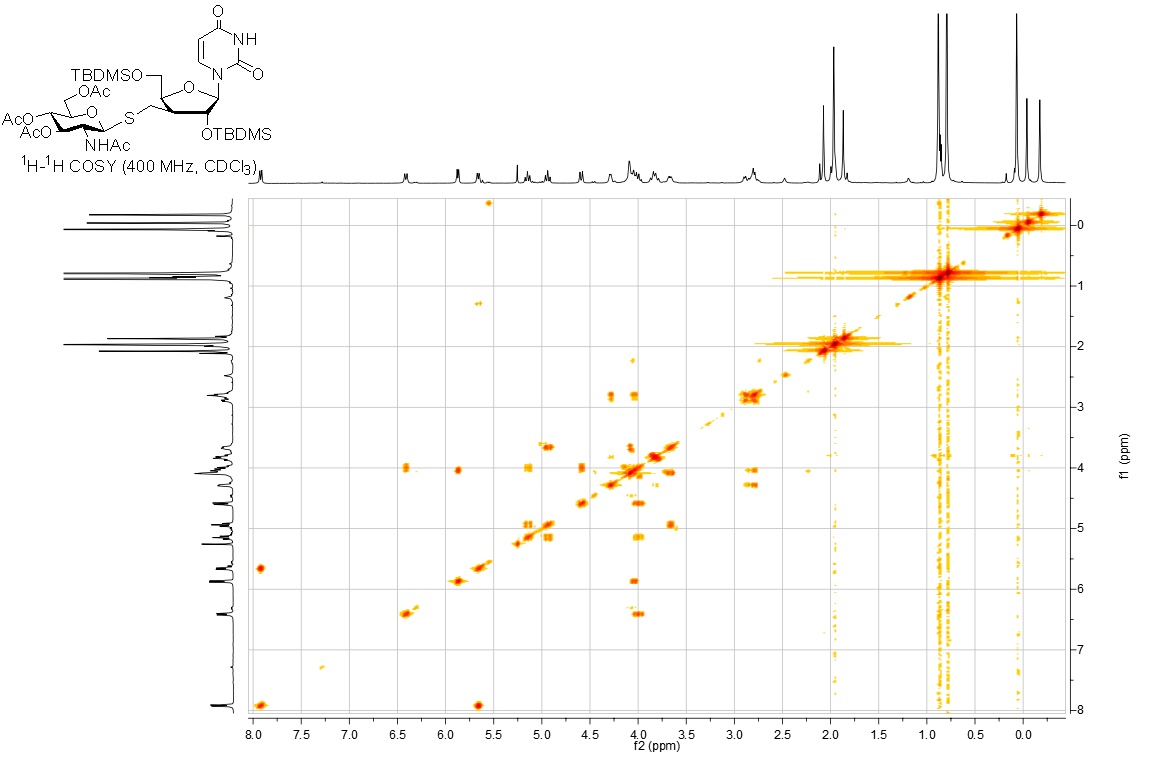


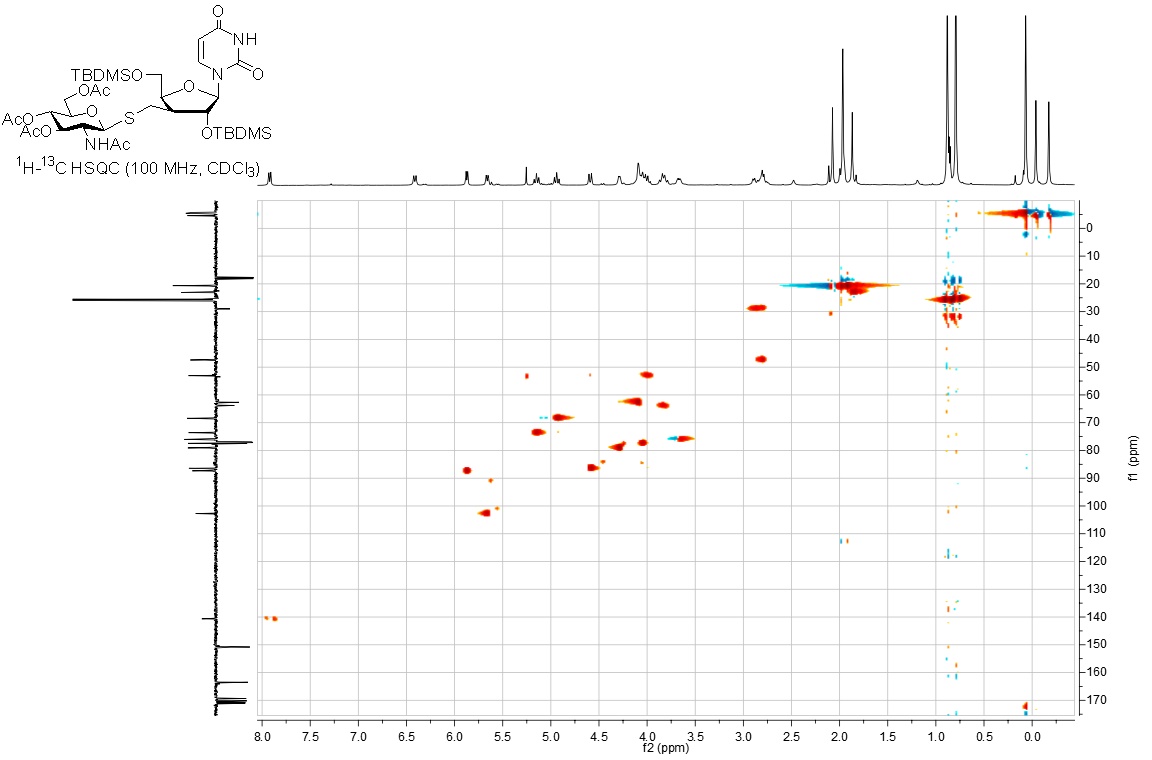


Compound **15**


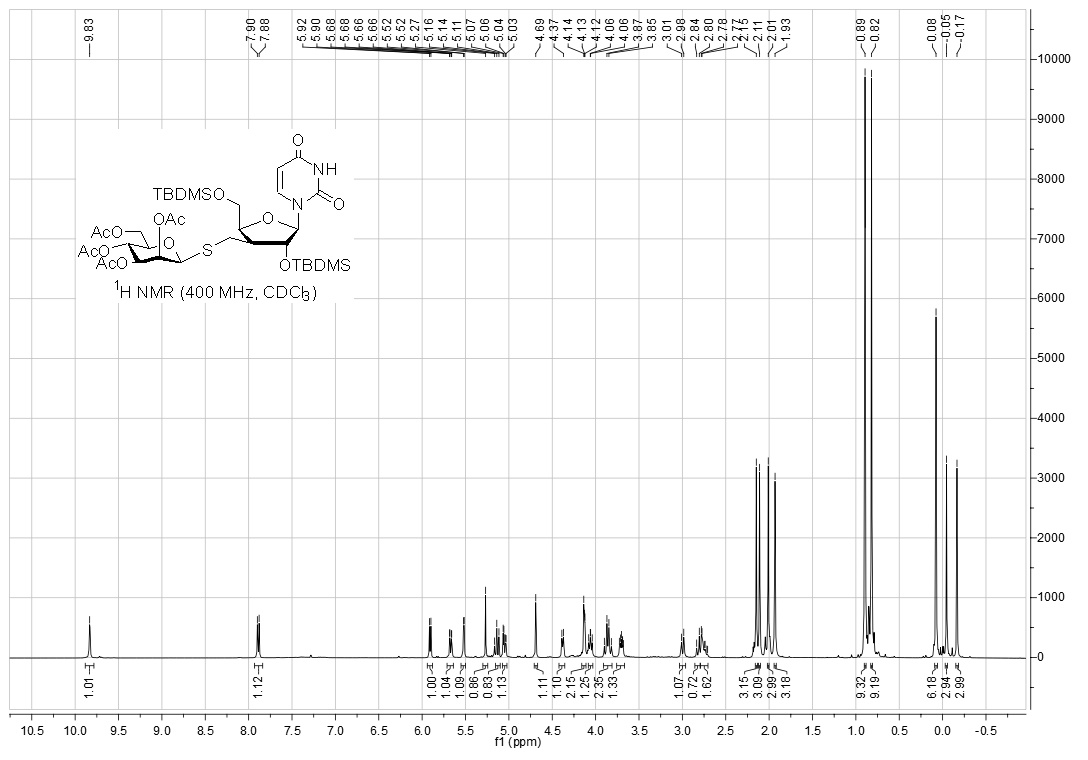


**
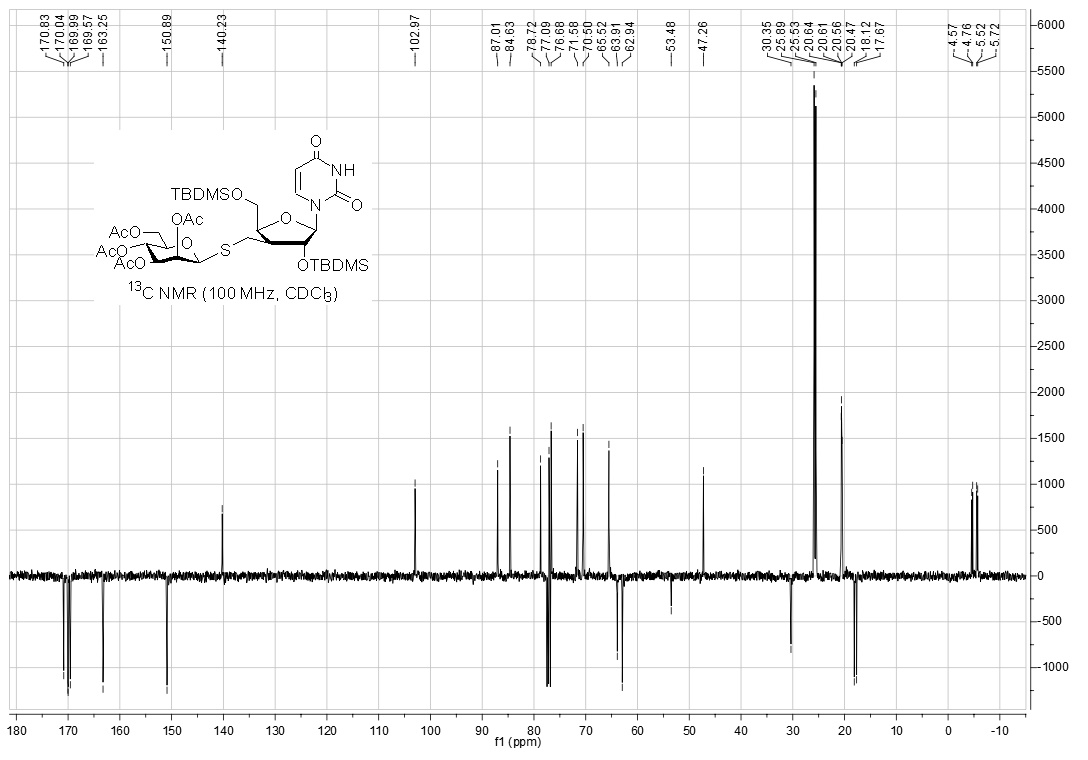
**


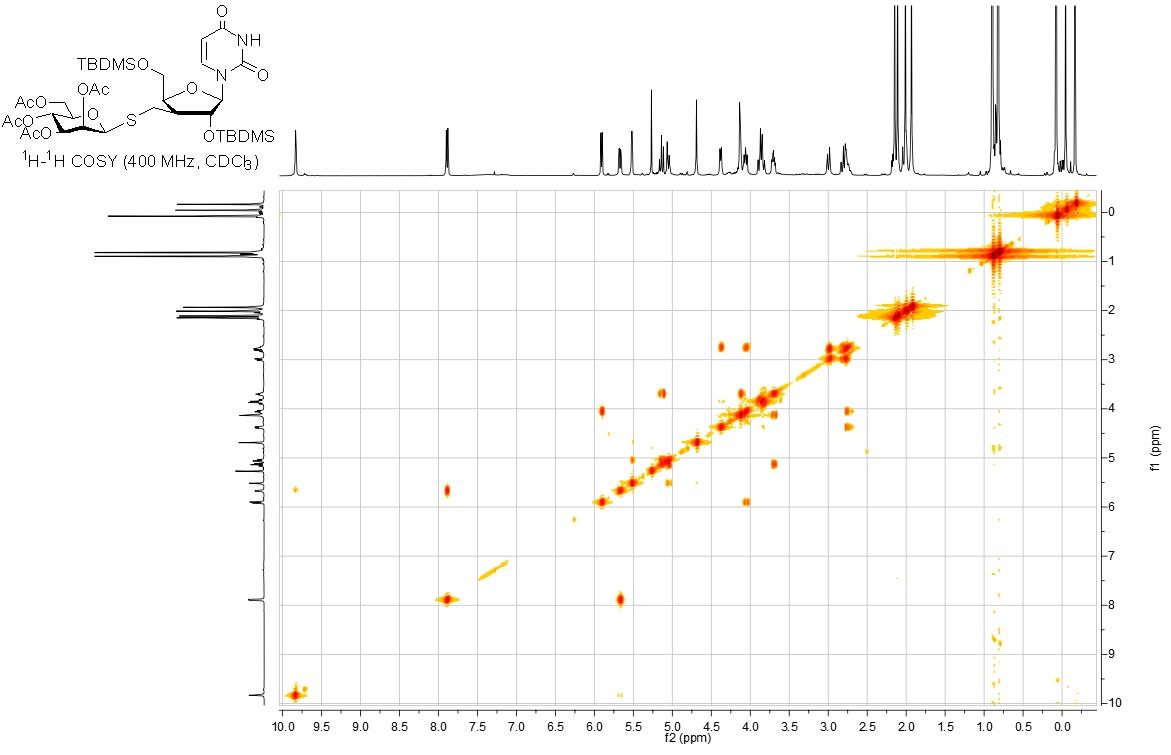


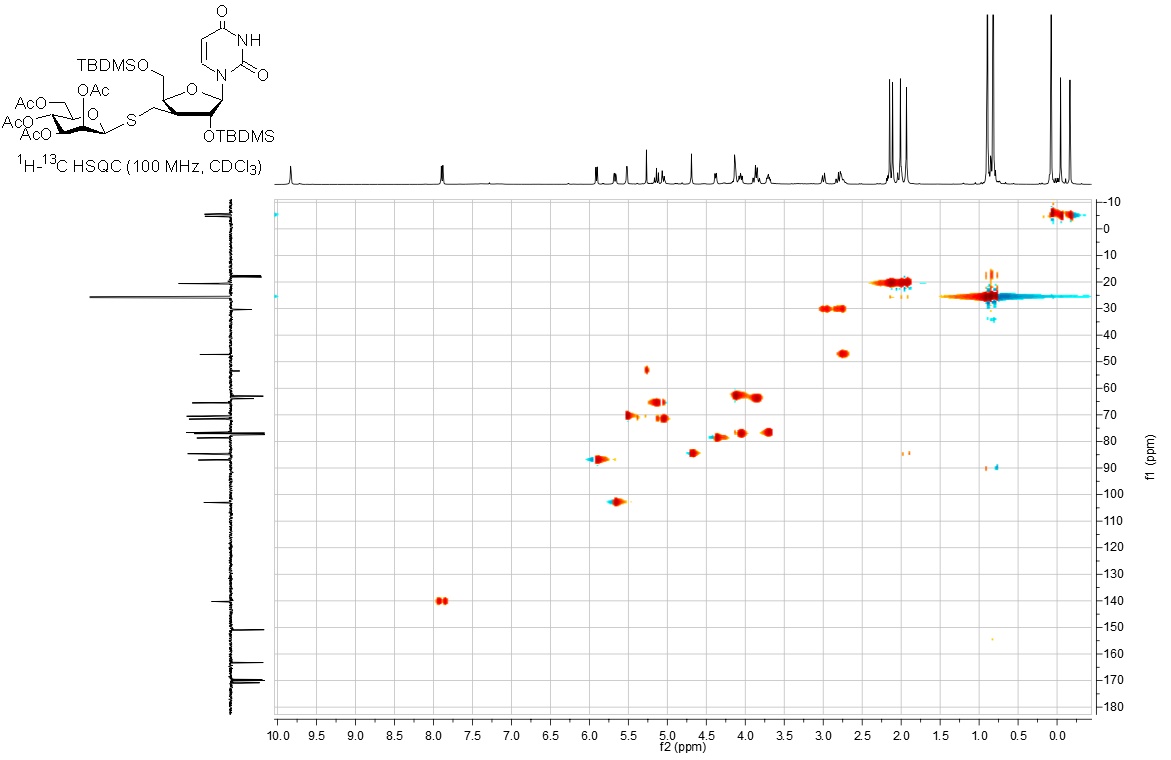


Compound **16**


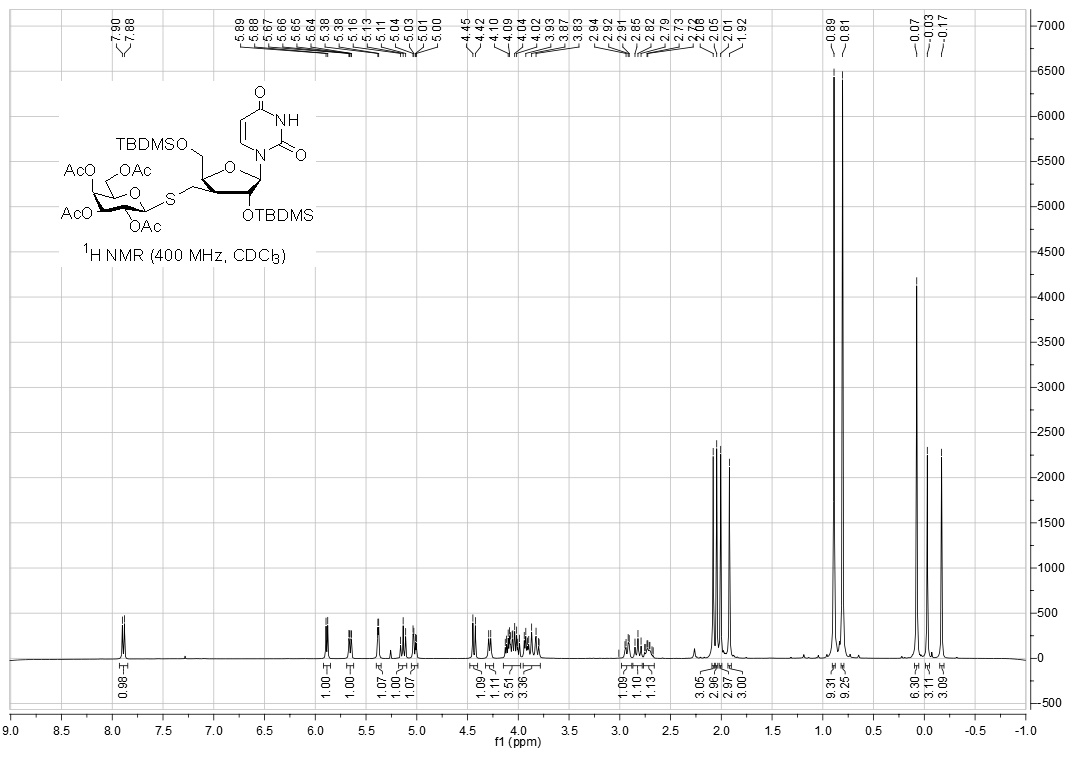


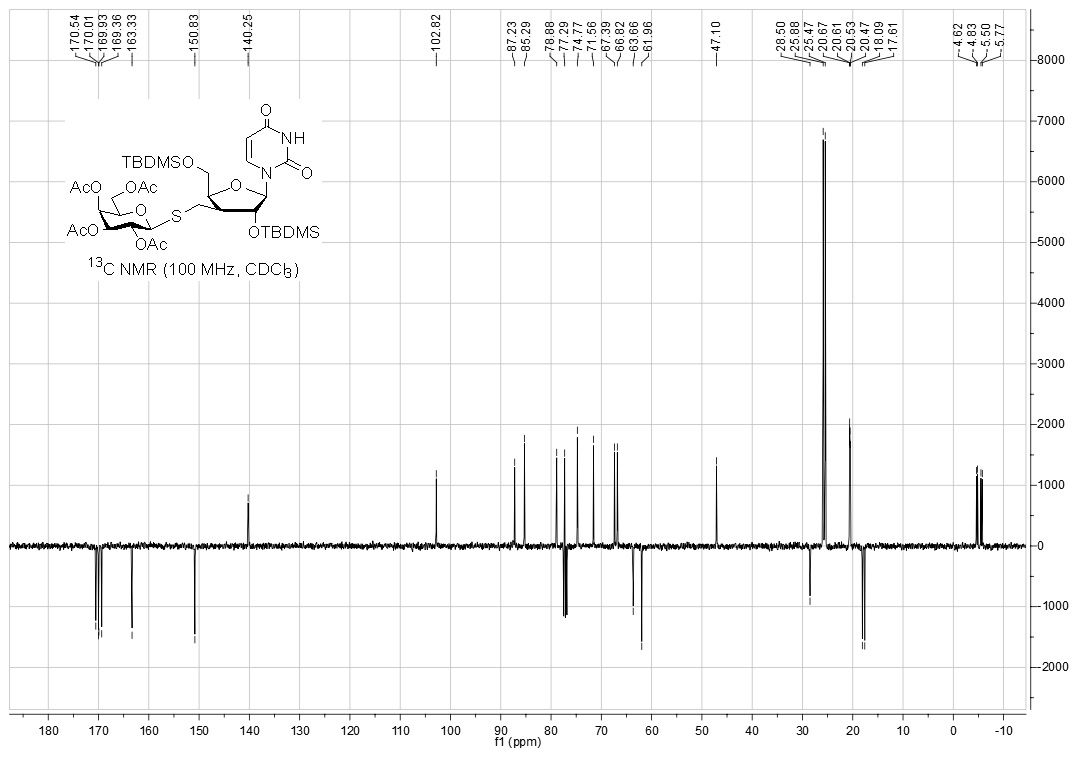


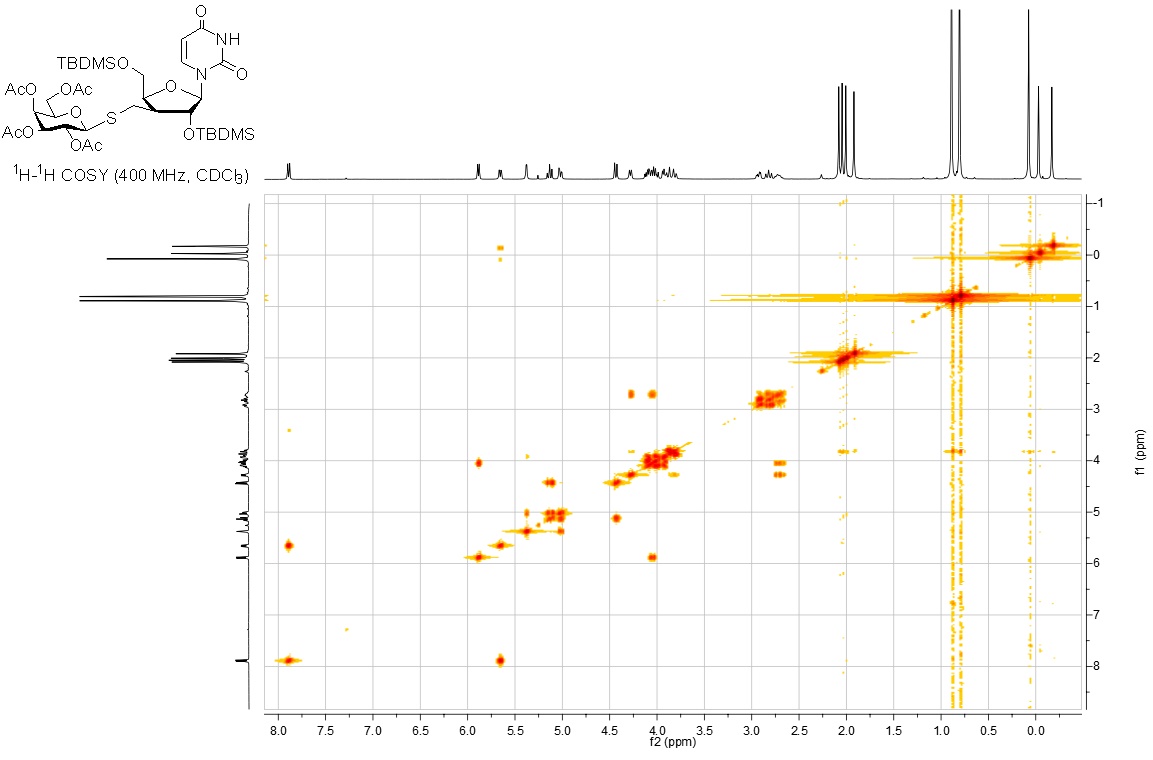


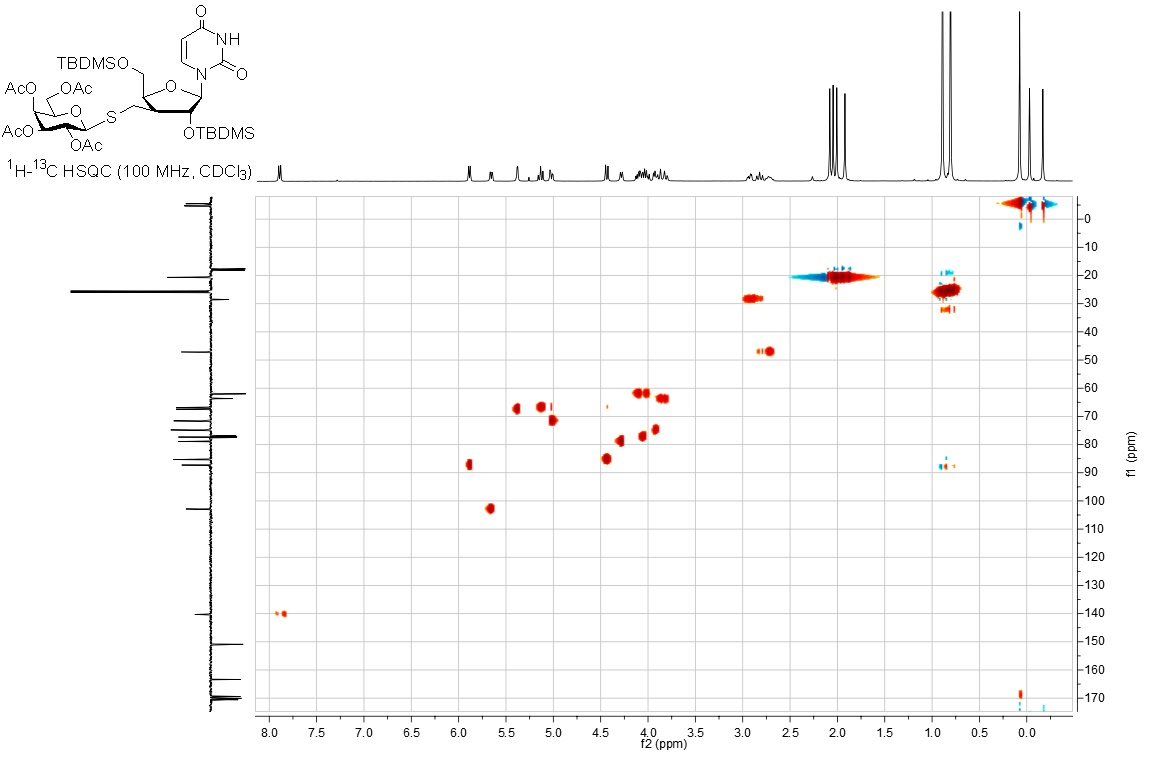


Compound **17**

**
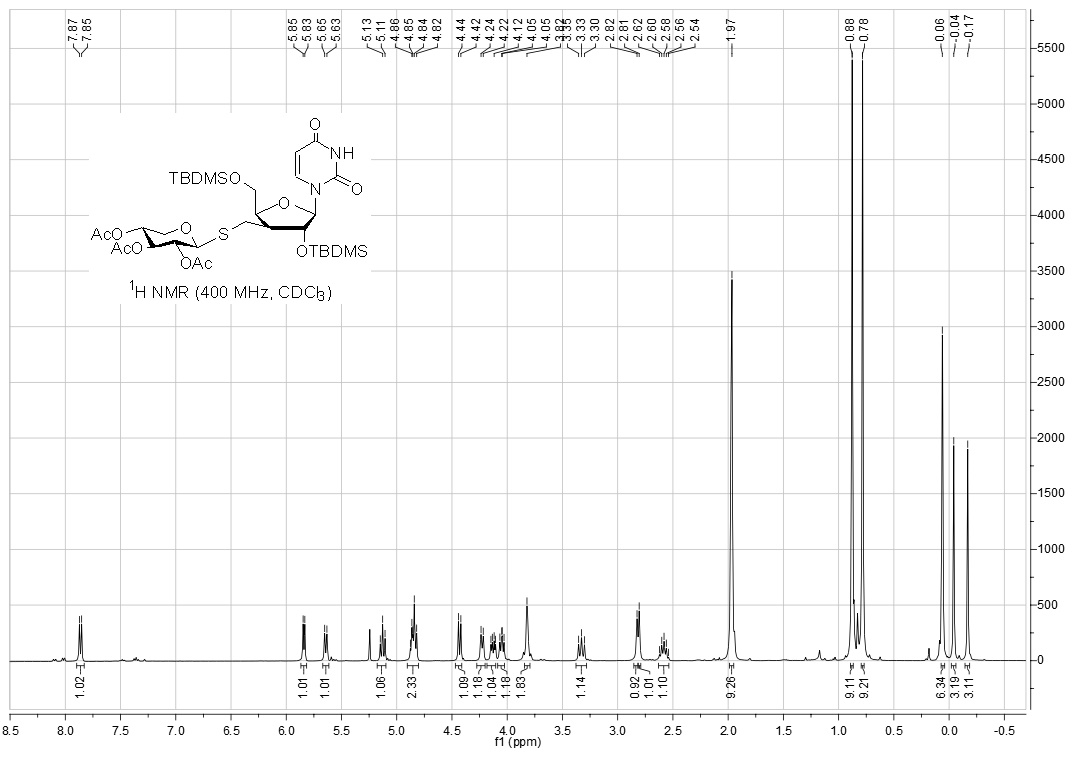
**


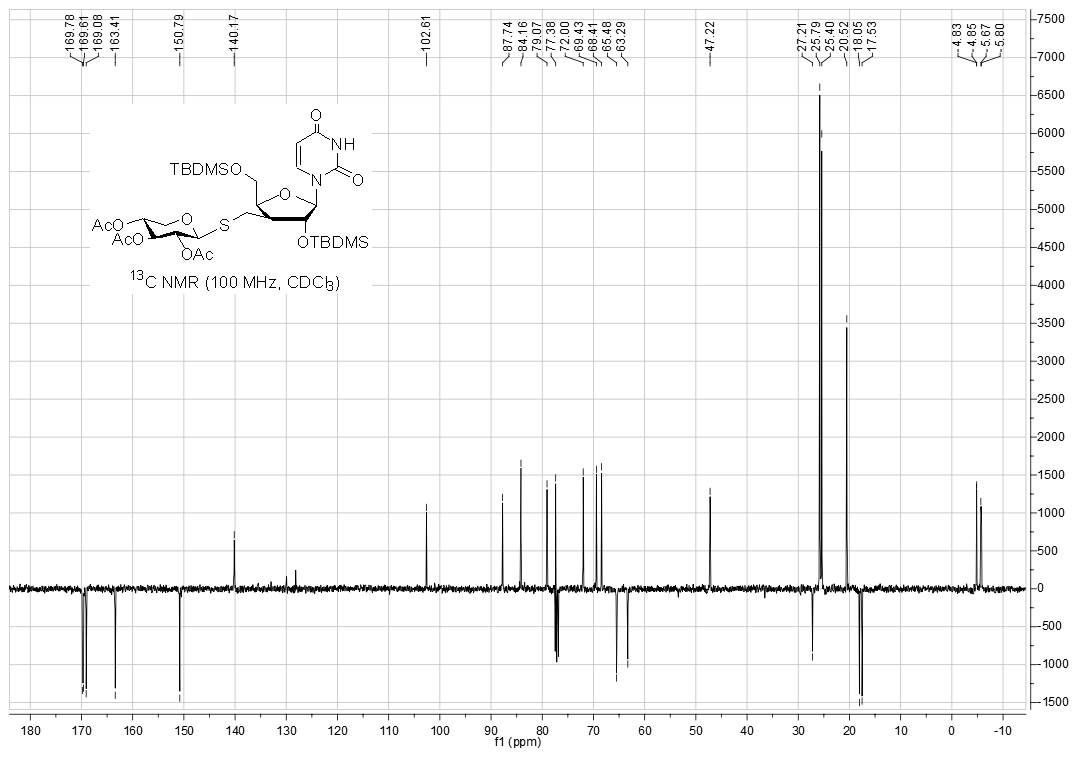


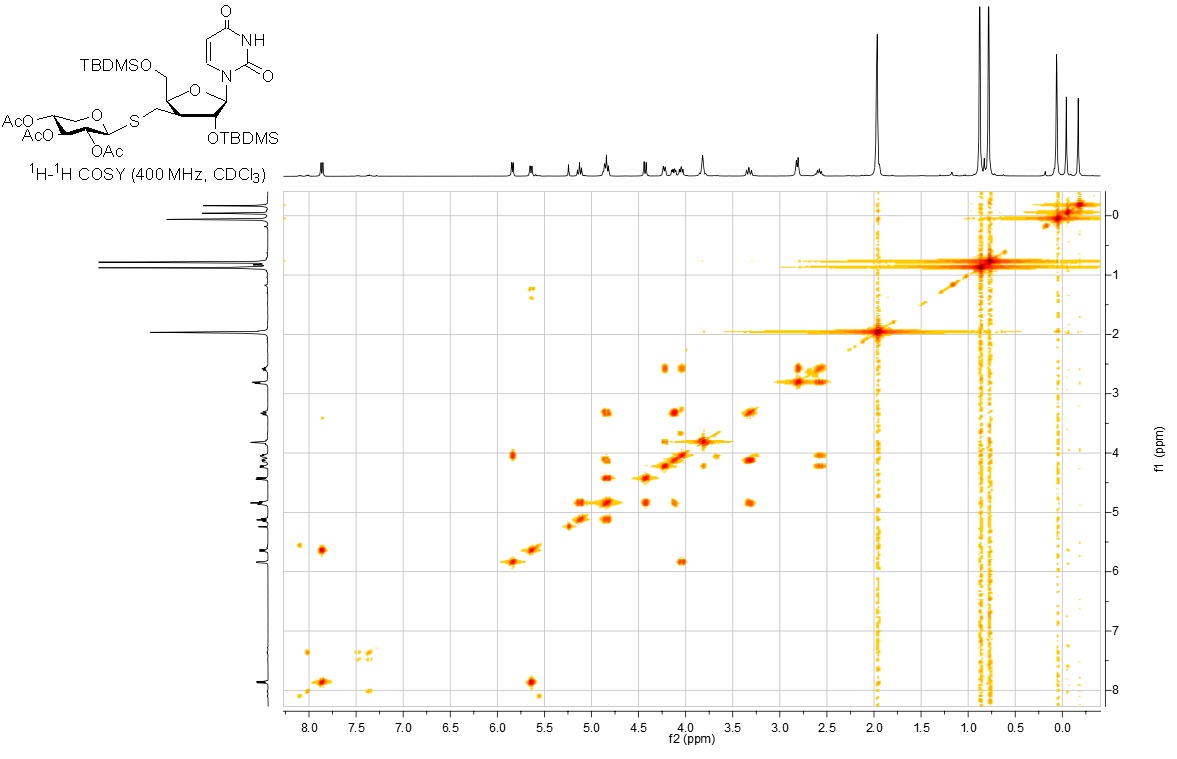


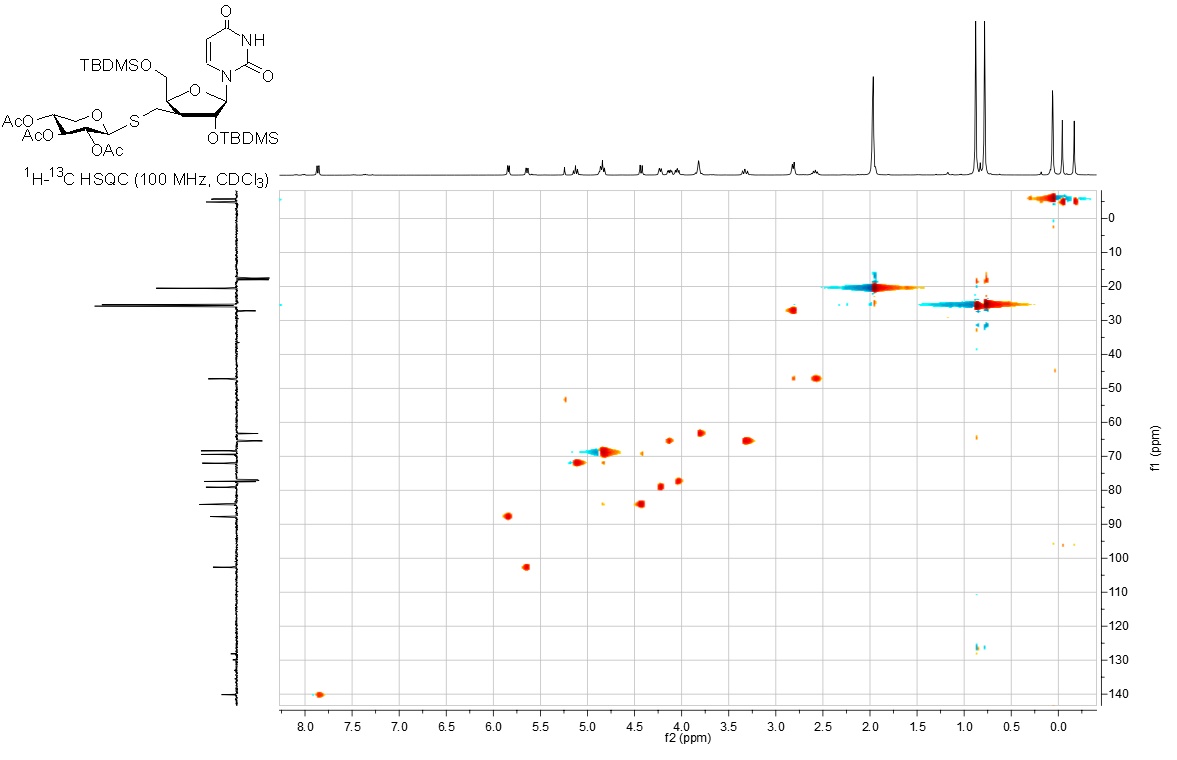


Determination of the diastereomeric ratio of compound **17** on the basis of the H-6 signals of the D-*ribo* and D-*xylo* isomers


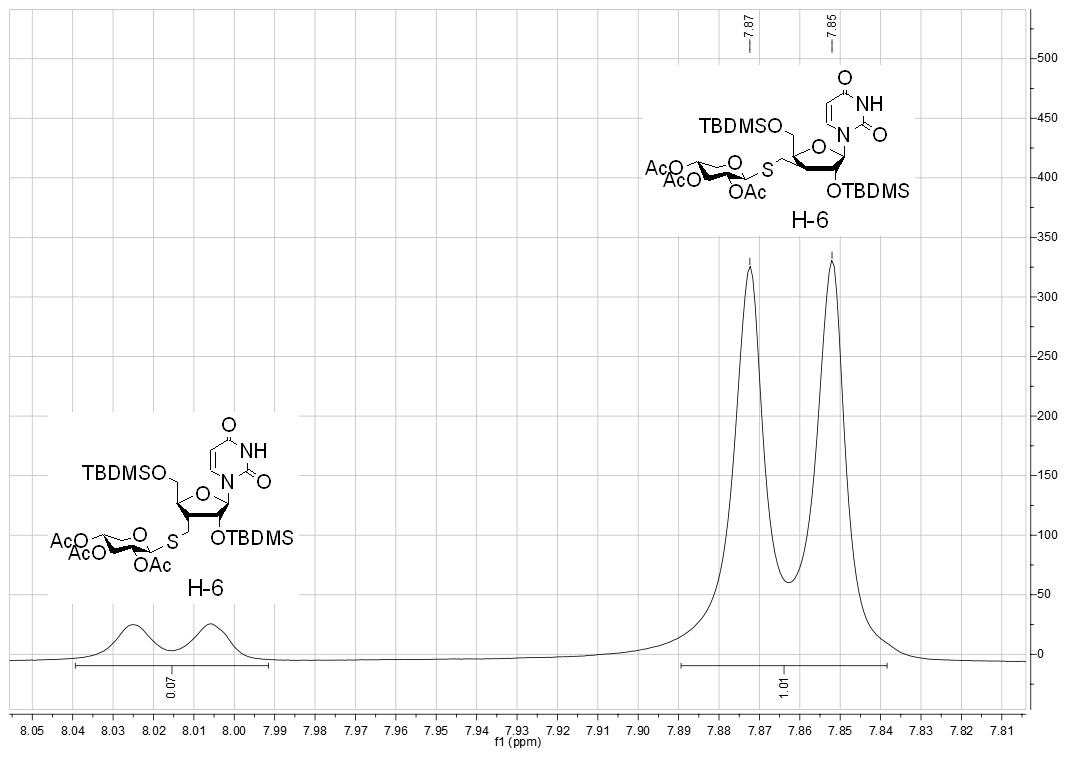


Compound **18**


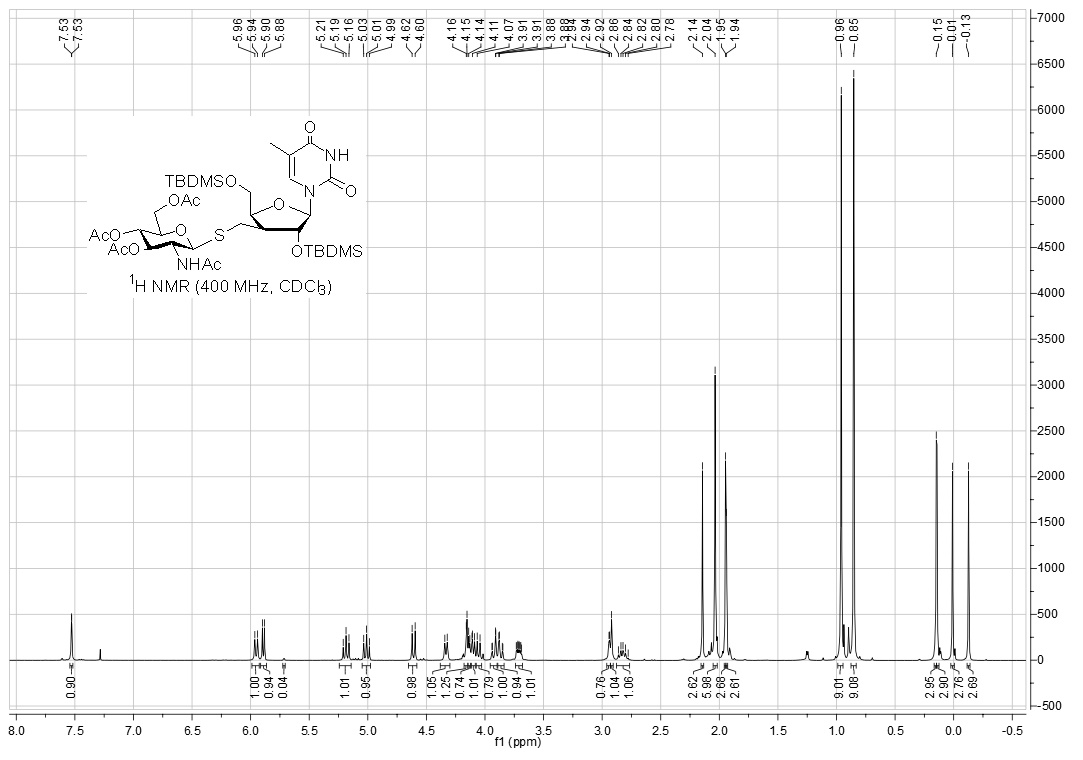


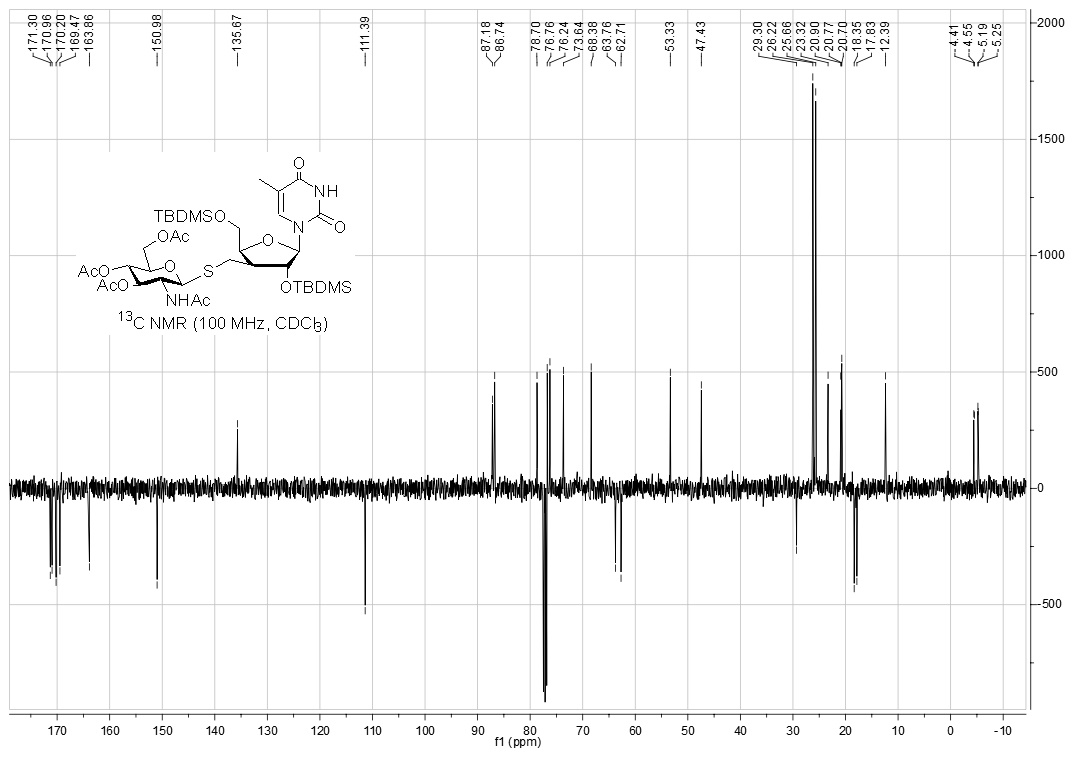


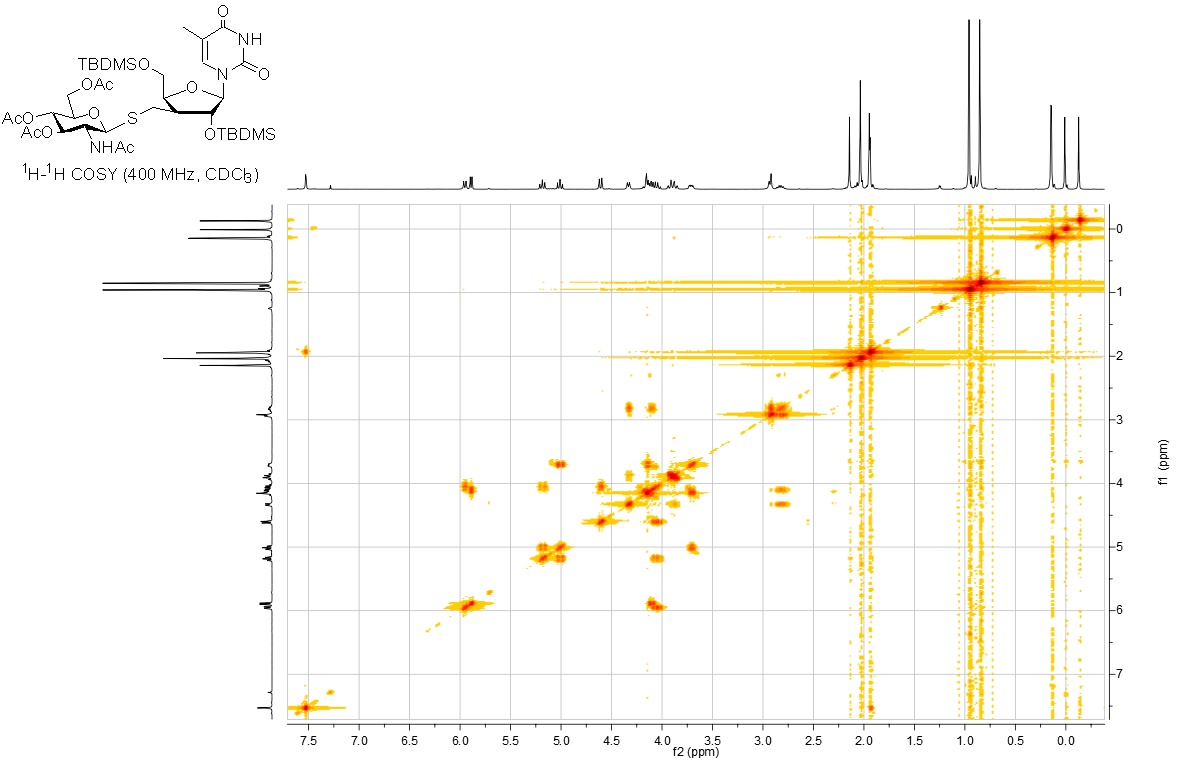


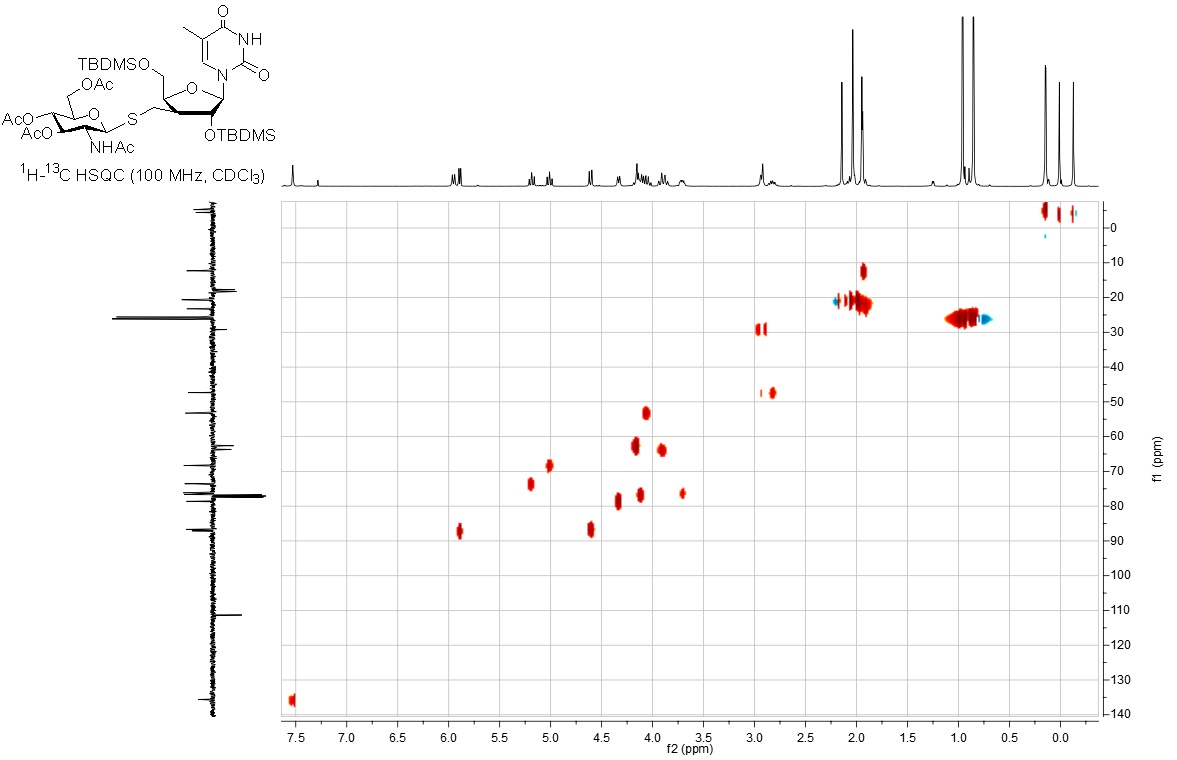


Determination of the diastereomeric ratio of compound **18** on the basis of the H-1’ signals of the D-*ribo* and D-*xylo* isomers


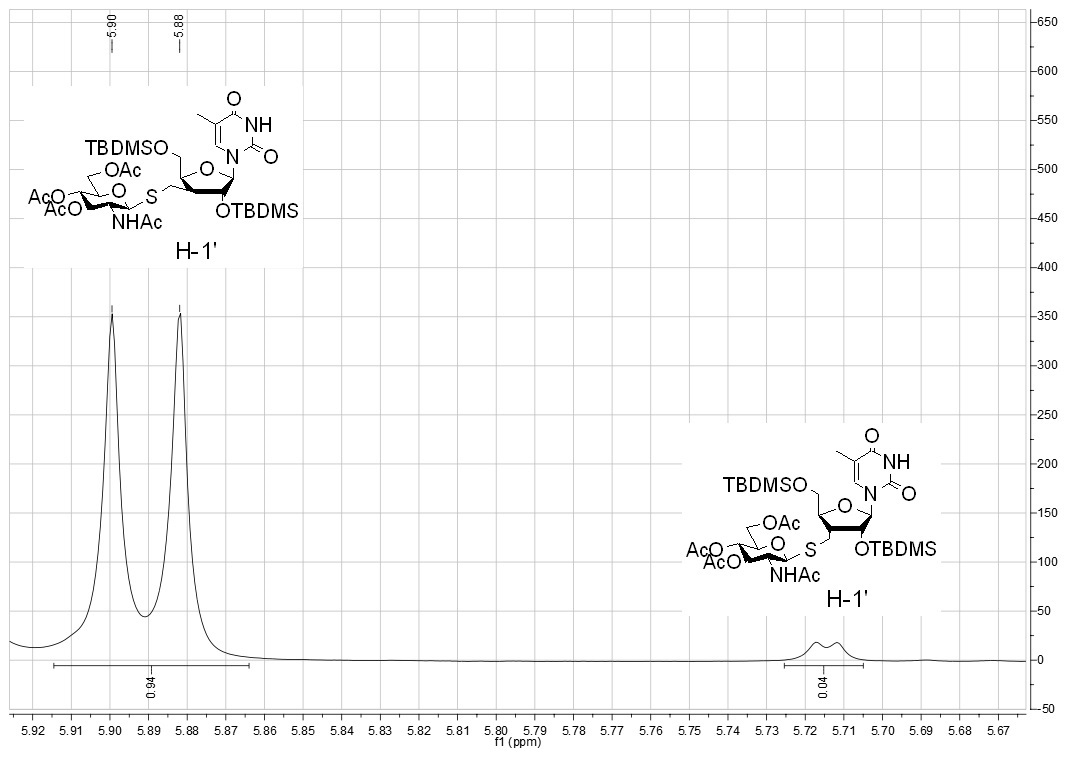


Compound **20**


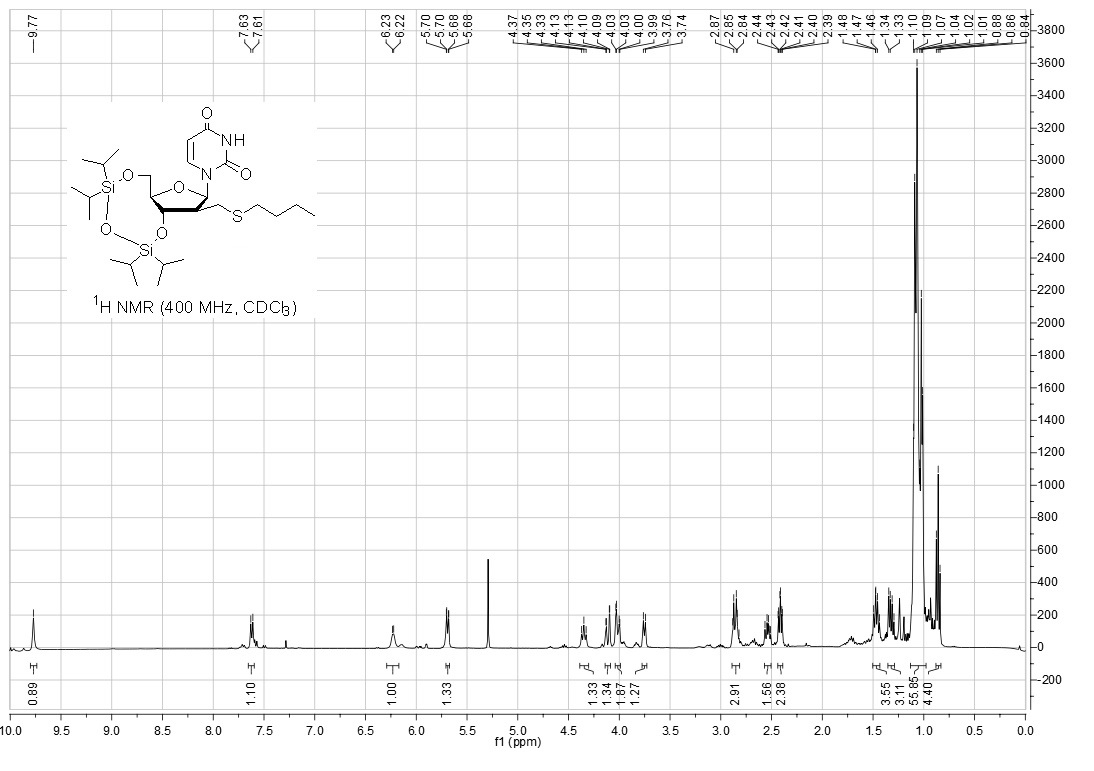


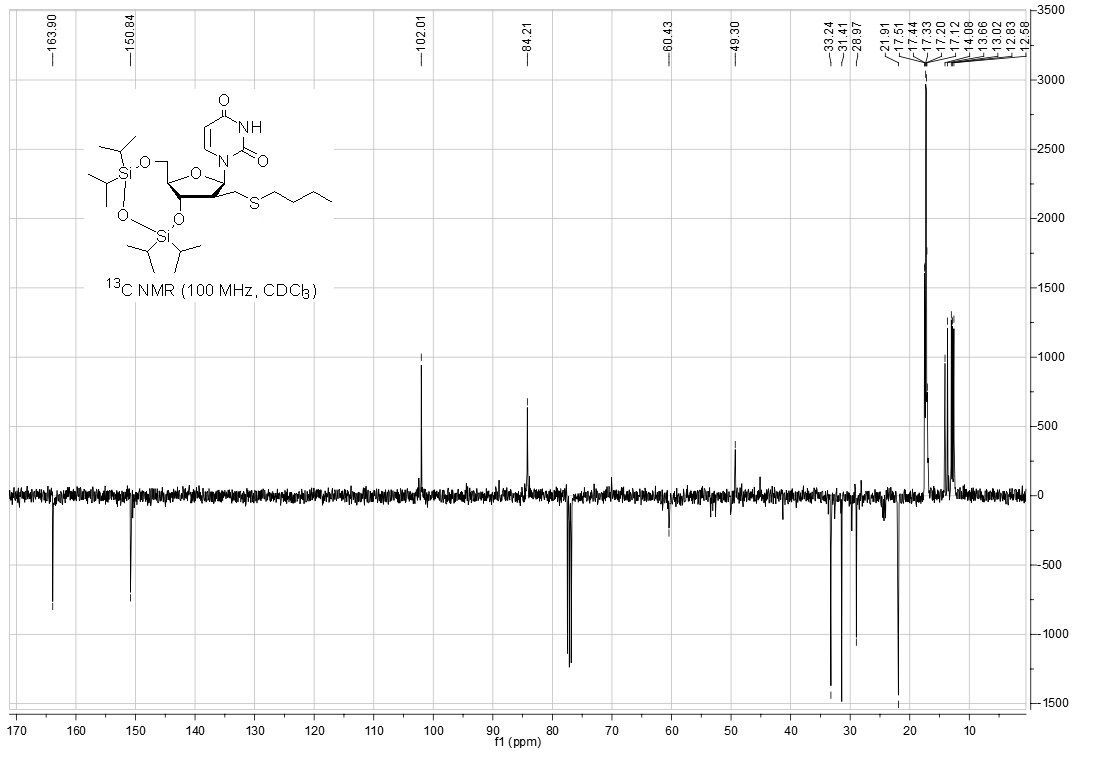


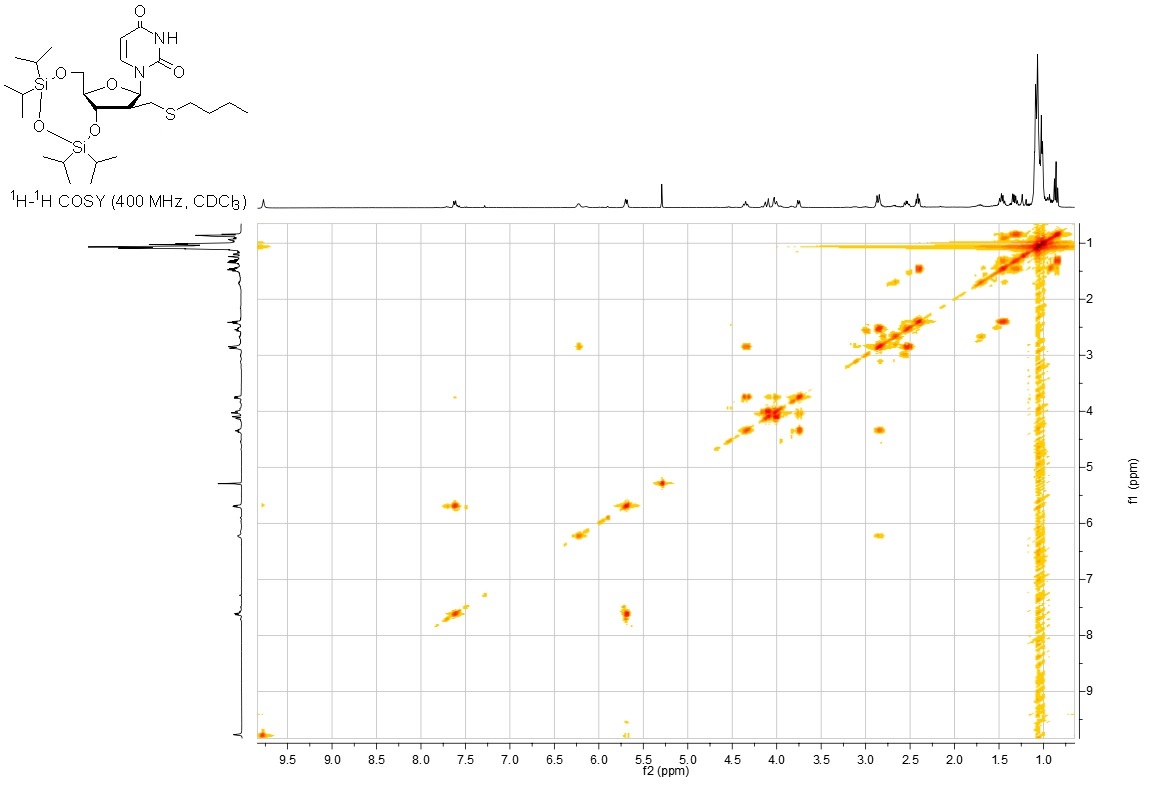


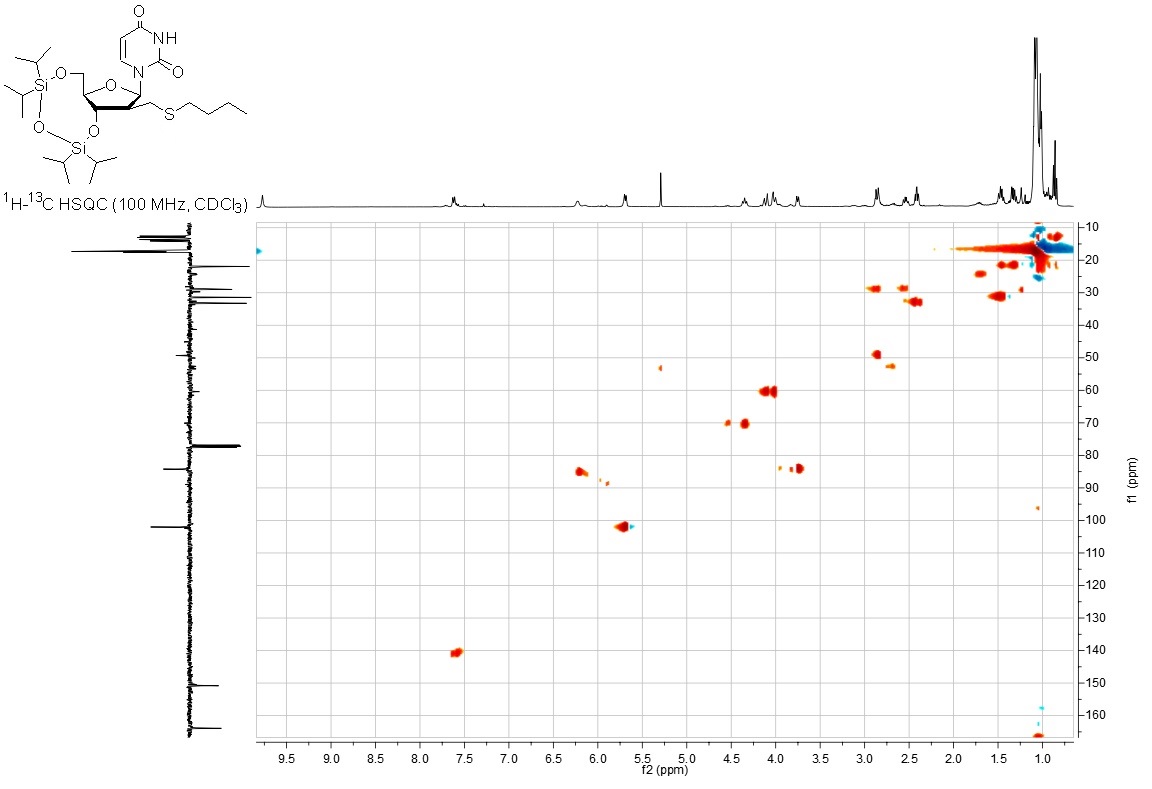

Supplement: Supplementary file 1 — Supplementary Information. [file 41598_2023_39541_MOESM1_ESM.docx]
